# 5^th^ Brazilian Congress of Geriatric Neuropsychiatry: September 5-7, 2013 - Centro Fecomercio de Eventos/São Paulo

**Published:** 2013

## Oral Session

### GERIATRIA/GERONTOLOGIA

**EFEITO DOS PROGRAMAS DE EDUCAÇÃO EM DEMÊNCIA NO CONHECIMENTO E NA ATITUDE DOS PROFISSIONAIS DA SAÚDE: REVISÃO INTEGRATIVA DE LITERATURA** *Maria Cristina Paes Mangieri; Gislaine Desani da Costa*

**RESUMO.** A demência é uma doença degenerativa em que há comprometimento da memória e de pelo menos uma das outras funções cognitivas, tais como linguagem, gnosia e praxia, ou funções executivas. Interfere no desempenho social e profissional do indivíduo de tal modo que as famílias, por não terem conhecimento do tema, ou aprisionem suas vítimas em casa por falta de recursos ou as internem em instituições de longa permanência para serem acompanhadas por profissionais da saúde. Estes, por sua vez, muitas vezes não estão preparados para atender esses pacientes, sendo recomendado que participem de programas em educação em demência. Optou-se por realizar uma revisão integrativa da literatura para verificar os efeitos desses programas no conhecimento e nas atitudes de profissionais da saúde. Os objetivos foram: identificar publicações científicas sobre programas de educação em demência para profissionais da saúde publicados em periódicos nacionais e internacionais dos últimos 10 anos e verificar os efeitos no conhecimento e na atitude de profissionais da saúde em relação ao rastreamento e acompanhamento do portador de demência e seu cuidador. A consulta foi feita nas bases de dados eletrônicas LILACS, IBECS, MEDLINE, Biblioteca Cochrane e no diretório eletrônico SCIELO. A busca ocorreu em fevereiro de 2013 e utilizaram-se os seguintes descritores: “Knowledge”, “Health Personnel”, “Dementia”, “Education”. O levantamento bibliográfico identificou 101 publicações e, destas, foram selecionadas 14 por atenderem aos critérios de inclusão no estudo. Os achados revelaram predomínio de estudos com delineamento qualitativo-quantitativo, publicados entre os anos de 2005 e 2006, desenvolvidos em diversas áreas do conhecimento, em especial na Enfermagem e na Geriatria. Os profissionais avaliados foram em sua maioria médicos, enfermeiros e auxiliares de enfermagem. Um único estudo também avaliou assistente social, terapeuta ocupacional e nutricionista. Os resultados evidenciam melhora do conhecimento desses profissionais, tanto em relação ao diagnóstico, como no acompanhamento e suporte ao portador de demência e seus familiares. Assim, sugere-se que programas de educação em demência sejam estendidos a todos os profissionais de saúde para que melhorem suas práticas no atendimento ao portador de demência e seus familiares, proporcionando assim, uma melhor qualidade de vida a ambos.

**EM BUSCA DA EXCELÊNCIA DE UM CENTRO DIA PARA IDOSOS: A SATISFAÇÃO DOS FAMILIARES** *Mardônio Ferreira da Silva; Beatriz Aparecida Ozello Gutierrez; Maria Helena Silveira*

**RESUMO. Introdução:** Com a transição demográfica e epidemiológica, observamos que idosos fragilizados requerem cada vez mais de familiares e de instituições públicas para a realização de suas atividades básicas da vida diária. Portanto, visando a integralidade do cuidado para manter a qualidade de vida desses idosos, essa assistência pode ser fornecida em Centro Dia para Idosos (CDI). Assim, é essencial o levantamento de dados que mostrem a satisfação dos familiares de idosos fragilizados nessas instituições. **Objetivos:** Analisar a satisfação dos familiares de idosos fragilizados usuários de CDI; avaliar a satisfação dos gerentes familiares e cuidadores familiares desses idosos; comparar os índices de satisfação entre os gerentes e familiares cuidadores desses idosos e sugerir estratégias de melhorias para as chances de alcançar a excelência do CDI. **Método:** A pesquisa é quantitativa de caráter exploratório, descritivo e transversal. Foi realizada em um CDI no município de São Paulo. A amostra contou com 19 participantes familiares de idosos usuários de CDI que responderam ao instrumento intitulado “Questionário de Avaliação do Grau de Satisfação do Cliente”. Salienta-se que dois dos participantes além de familiares também exerciam o papel de gerentes do CDI. Os dados foram inseridos em banco de dados por meio do Programa Excel e os procedimentos relacionados ao gerenciamento foram organizados segundo modelo estratégico Balanced Scorecard (BSC) e pelo modelo teórico das cincos falhas (5 Gaps) e ainda, pelo modelo operacional diagrama de Ishikawa com 6 M. Desta forma, identificamos dados relacionados à infra-estrutura e ao método de trabalho no CDI conforme a visão do cliente. **Resultados:** Os domínios Infra-estrutura e Relação Familiar/Instituição são os itens causadores de maior insatisfação (média de 70% e 71% de satisfação) aos familiares dos idosos. Constatamos discordâncias entre a avaliação dos gerentes e dos familiares. **Conclusão:** Os resultados indicam necessidades de mudanças para o alcance da excelência do serviço, da qualidade assistencial prestada ao idoso/família e ainda, a importância do gestor do CDI ter competências em Gerontologia. Acreditamos que esses resultados desperte o interesse de empreendedores favorecendo a ampliação do número de CDI no Brasil e propiciar a existência de organização que favoreça o desenvolvimento do ensino e pesquisa na área da Gerontologia.

**FORMER TOBACCO EXPOSITION CAN WORSEN THE PERFORMANCE IN A COGNITIVE STIMULATION AND REHABILITATION PROGRAM BASED IN COMPUTERS AND INTERNET USE** *André Junqueira Xavier; Henrique Dircksen Melo; Eleonora d‘Orsi*

**ABSTRACT. Introduction:** There is evidence that Cognitive Stimulation and Rehabilitation (CSR), can reduce cognitive decline, revert Mild Cognitive Impairment and delay dementia. But many factors can impair cognition, such as chronic diseases and lifestyle habits. It's important to evaluate if tobacco exposure can affect the performance of CSR in order to develop better methodologies. **Objective:** To evaluate the influence of tobacco exposure in a program of cognitive stimulation and rehabilitation mediated by computers and internet. **Methods:** Open cohort, controlled and non randomized (quasi experimental), study. Data collected between 2008 and 2012, in two cities of Santa Catarina (Tubarão and Palhoça). Data about chronic morbitidies, sociodemographic profile, lifestyle (tobacco exposure), medications, functional status (initial and final), Mini-Mental Status Examination (initial and final) were collected among participants aged 50 or older of a 12 week cognitive stimulation and rehabilitation program based in computers and internet use. All participants were from the community, with memory complaints, non demented. The outcome was the variation of the Mini-Mental State Examination (MMSE) before and after the program. Final model obtained by multivariate linear regression after univariate and bivariate analysis. This study was approved by Brazilian National Health Council (ethics in research). **Results:** 194 participants, female 166 (85.6%), age 64.67±6.85 years, 8.32±4.66 years of schooling, never smokers 134 (69.1%), former smokers 50 (25.7%), still smoking 10 (5.15%) initial MMSE 26.02±2.76, final MMSE 27.40±2.24. Former smokers had a MMSE positive variation of 0.70±2.03 x 1.63±2.44 from never smokers in (p=0.011). Initial MMSE (p=0.000) and years of study (p=0.025) were also significant and independent variables in relation to a lower variation of the MMSE after CSR. The analysis was controlled by age, years of schooling, marital status, social status, gender, diabetes, hypertension, hypothyroidism, dyslipidemia, stroke, myocardial infarct, peripheral arterial insufficiency, BMI, sedentarism, depression, number of continuous medications, use of benzodiazepinics, functional capacity and time elapsed between initial and final interview. **Conclusion:** Former smokers had a worse performance compared to those who have never smoked in a CSR program, reinforcing the need for preventive education and basic health care's prevention.

**FUNÇÕES EXECUTIVAS, ATIVIDADES DA VIDA DIÁRIA E HABILIDADE MOTORA DE IDOSOS COM DOENÇAS NEURODEGENERATIVAS** *Lilian Assunção Felippe; Renata Terra de Oliveira; Nádia Meneguesso Calheiros; Milena Garcia; José Eduardo Peres; Gustavo Christofoletti*

**RESUMO.** Processos neurodegenerativos exercem grande influência sobre a qualidade de vida e a independência do paciente, levando comumente à situação de exclusão social. **Objetivo:** Analisar as funções executivas de idosos com doença de Parkinson (DP - com e sem quadro demencial) e de Alzheimer (DA), e confrontar os escores dos participantes no que se refere as atividades funcionais da vida diária e a habilidade motora em situações de dupla-tarefa. **Métodos:** Sob um desenho transversal, 54 idosos foram divididas em 4 grupos: G1, composto por 11 sujeitos com DP; G2, formado por 10 sujeitos com demência de Parkinson; G3, composto por 13 participantes com DA, e; G4 formado por 20 idosos saudáveis. Os procedimentos metodológicos envolveram análise das funções cognitivas pré-frontais dos sujeitos, da realização das atividades da vida diária e da habilidade motora em situações de duplas-tarefas. A análise dos dados envolveu a estatística descritiva (média e erro-padrão) e inferencial (teste ANOVA e post hoc de Scheffé), admitindo significância de 5% (p<0,05) e intervalo de confiança de 95%. **Resultados:** As funções cognitivas pré-frontais apresentaram diferença significativa entre os grupos, sobretudo nas comparações envolvendo G2 e G3, em relação a G1 e G4 (p=0,001). Os grupos com déficit cognitivo apresentaram pior rendimento na realização das atividades da vida diária, com pior escore do G2, onde há junção de déficit cognitivo e motor (p=0,001). Em situações de dupla-tarefa, G2 e G3 apresentaram pior desempenho que demais grupos. **Conclusão:** Distúrbios pré-frontais repercutem negativamente nas atividades funcionais e na habilidade psicomotora dos indivíduos. Quando não vinculado a quadro demencial, os pacientes com DP apresentaram escores cognitivos pré-frontais e independência funcional semelhantes a idosos saudáveis.

**MANUAL DEXTERITY ASSESSMENT ON HEALTHY AGING PROCESS** *Ralf Braga Barroso; Elaine Andrade Moura; Thamara Cunha Nascimento Amaral; Cláudia Helena Cerqueira Mármora*

**ABSTRACT. Introduction:** The manual dexterity is definite as the ability of manipulate objects with the fingers skillfully and controlled. According to the Theory of Retrogenesis, the fine motor abilities are the last to be acquired in adult life, so, they are the first to be lost with the aging process. That process happens due, mainly, the atrophy in areas as the pre-frontal cortex, which has execution, organization and planning functions on movements. **Objetive:** To evaluate whether the healthy aging process affects significantly the manual dexterity and whether the sociodemographic variables are process-related. **Methods:** We applied a questionnaire with the following socio-demographic variables: age, scholarity and economic classification. Besides, was applied the Mini Mental State Examination in order to the cognition assessment, and the Nine Hole Peg Test was used in order to evaluate the manual dexterity. This research was realized at the Polo Cultural Enrichment for Third Age of the Federal University of Juiz de Fora (UFJF) and Community Center Dona Itália Franco, Juiz de Fora, Minas Gerais, Brazil. The statistical analyze was done using the software SPSS 20.0 and, to verify the correlation between the variables we used the Pearson correlation coefficient (p<0.05). Every participant signed the consentient term and this study was approved by the Ethics Committee on Human Research of UFJF in the opinion 238,469/2013. **Results:** 97 people aged 60 years or more participated of this study, which 9 are males and 89 females, the mean age was 72.35 years (SD±4.88). We found a good positive correlation between age and manual dexterity (r=0.614; p=0.001). However, the correlation between dexterity, cognition (r= -0.296; p=0.003) and economic classification (r=0.269; p=0.008) presented a very low correlation. The correlation between dexterity and scholarity was not significant statistically (r= -0.289; p=0.289). **Conclusion:** We concluded with these results that the manual dexterity decreases on aging process and this may reinforce the Theory of Retrogenesis in the aging.

**OFICINAS DE ESTIMULAÇÃO E REABILITAÇÃO MEDIADAS POR COMPUTADORES E INTERNET MELHORAM A FUNÇÃO COGNITIVA DE PESSOAS IDOSAS, ESTUDO DE INTERVENÇÃO CONTROLADO** *André Junqueira Xavier; Eleonora d‘Orsi; Rodrigo de Rosso Krug*

**RESUMO.** A manutenção da capacidade cognitiva é fundamental para idosos e fator de risco para mortalidade. O uso da estimulação e reabilitação para retardar o declínio cognitivo se apoia em evidências consistentes advindas de ensaios clínicos controlados e randomizados e de metanálises. **Objetivo:** Estimar o efeito de um programa de estimulação e reabilitação cognitiva mediado por computadores e internet na variação estado cognitivo de pessoas com 60 anos ou mais medida pelo Mini Exame do Estado Mental (MEEM). **Método:** Estudo de intervenção controlado do tipo quasi experimental “Oficina da Lembrança”(OL) de 2008 a 2012 com pacientes não demenciados, acompanhados nos serviços médicos da UNISUL, residentes em Palhoça, Tubarão e Florianópolis-SC. O programa com duração de 20 sessões bissemanais de 1,5 hora cada. A variável desfecho foi o percentual de mudança entre o primeiro e o segundo MEEM levando-se em conta o efeito teto e efeito solo segundo a fórmula: quando MEEM final (MEEM2) >MEEM inicial (MEEM1), ∆Y*=((MEEM2-MEEM1) / (30-MEEM1)) x 100. Quando MEEM2< MEEM1, ∆Y=((MEEM2MEEM1) / (MEEM1)) x 100. Quando MEEM2=MEEM1, ∆Y=0. Foi utilizado o pacote estatístico STATA. Para a identificação dos fatores associados ao efeito do programa na variação percentual do MEEM, usou-se análise bivariada e multivariada com regressão linear, p≤0,05. Variáveis de controle: sociodemográficas, comorbidades, atividade física, exposição ao tabaco, IMC, capacidade cognitiva inicial (MEEM1) e capacidade funcional. Considerou-se o efeito de delineamento do estudo. Aprovação CEP/ UNISUL no 1663.07. **Resultados:** Analisados 293 participantes, 160 casos e 133 controles, escolaridade média de 8,59±0,25. Sem diferença no MEEM1 entre os casos com 25,95±2,95 e controles 26,41±3,58, p=0,24. Os que participaram da OL apresentaram média de idade inferior 67,19±5,68 quando comparados aos controles 69,97±6,30, p=0,001; sem diferença significativa em termos de capacidade funcional e escolaridade entre os grupos. No modelo multivariado final houve variação percentual independente e positiva no MEEM entre aqueles que participaram da OL em relação ao controle de 24,39%; IC95%=14,86/33,91; p=0,000 e negativa em relação ao valor do MEEM1 (inicial) mais alto de -4,28%; IC95%= -5,68/-2,28; p=0,000 provavelmente por efeito teto. Outras variáveis não foram significativas. **Conclusão:** Oficinas de estimulação e reabilitação com computadores estão associadas à melhoria cognitiva de idosos.

**PERFIL COGNITIVO E FUNCIONAL DE IDOSOS ATENDIDOS NA ATENÇÃO PRIMÁRIA NO DISTRITO FEDERAL** *Andréa Mathes Faustino; Juliana Gracielle Rodrigues; Aline Cristina Martins Gratão; Keila Cristianne Trindade da Cruz; Carla Targino Bruno dos Santos*

**RESUMO.** O acolhimento ao idoso e seu acompanhamento pelos profissionais da Atenção Primária, é muito importante e faz a diferença, no que tange a prevenção e a detecção precoce dos agravos à saúde, com vistas a manutenção da integridade funcional e autonomia, principalmente no que diz respeito a funcionalidade cognitiva e de desempenho de funções de autocuidado e de tarefas mais complexas do cotidiano. **Objetivos:** Descrever o perfil cognitivo e funcional de idosos que frequentam uma Sala de Acolhimento ao Idoso, localizada no Centro de Saúde do Paranoá, região administrativa do Distrito Federal (DF). **Métodos:** Trata-se de um estudo transversal de base populacional, de caráter analítico observacional com amostra de conveniência, coletado em fonte de dados secundária em planilha do próprio serviço e prontuário. Pesquisa aprovada sob o protocolo n° 059/2012 CEP/SES-DF. Analisados dados sociais e resultados das avaliações: cognitiva pelo Mini-mental, das Atividades Básicas de Vida Diária (ABVD) por Katz e Atividades Instrumentais de Vida Diária (AIVD) por Lawton. **Resultados:** Fizeram parte da amostra 61 idosos, média de idade foi 71 anos, 60,6% mulheres, 52,4% cor branca, 47,5% casados, 88% moravam acompanhados de algum familiar e todos residiam na região administrativa onde se localiza o serviço de saúde. O diagnóstico mais prevalente nesta população foi a Hipertensão Arterial em 87%, dos casos, nenhum possuía diagnóstico para demências e todos eram independentes para locomoção. Quanto à escolaridade 58% possuíam um ou mais anos de estudo, 12% sabiam assinar nome e ler algumas palavras e 30% eram analfabetos. Pelo Mini-mental, 60% dos avaliados, conforme a escolaridade estava abaixo dos valores de escore proposto para o Brasil. Para este grupo analisado as alterações cognitivas ainda não estão afetando o desempenho nas ABVD tendo 75% dos idosos com a manutenção de total independência para todas as atividades, porém para as AIVD 50% dos idosos já apresentam alguma perda funcional para realizar atividades mais complexas. **Conclusão:** Apesar da maioria dos idosos ter capacidade funcional preservada para as ABVD, o déficit no teste cognitivo e a alterações na escala de AIVD, pode ser um indicativo de comprometimento cognitivo leve, fato que nos chama atenção para que esta população e seus familiares fiquem atentos a outras alterações que possam comprometer estas funcionalidades e vir a desenvolver alterações relacionadas a algum tipo de demência.

**PREVALENCE OF COGNITIVE IMPAIRMENT IN AFRICAN BRAZILIAN ELDERLY LIVING IN THE OLDEST ISOLATED COMMUNITY OF DESCENDENT OF SLAVES IN CENTRAL BRAZIL** *Danielly Bandeira Lopes; Leonardo Caixeta*

**ABSTRACT.** Brazil has one of the richest cultural and ethnic diversities in the world. Paradoxically, relatively little is known about cognitive functioning in aging African Brazilian and there are no studies with isolated communities of descendent of slaves (“quilombos”). The elderly population of ethnic minorities, like Afro-Brazilians, are underrepresented in dementia evaluation and care, especially because no account of cultural differences are done in most studies. The differences between ethnic groups in rates of dementia has important implications, as in health care policies and services. **Objectives:** We aim to estimate the prevalence of cognitive and functional impairment in elderly living in an isolated rural community of descendent of slaves, known as ‘Kalunga', in Central Brazil. **Methods:** A cross-sectional study with noninvasive methods, based on primary data of cognitive and functional elderly aged over 60 years, living in an isolated rural community of descendent of slaves, known as ‘Kalunga', in Central Brazil. Kalunga is the oldest ‘quilombo' in Brazil and is considered by federal law as cultural heritage site of historical value. Data on cognitive and functional assessment were obtained through the application of the Mini-Mental State Examination (MMSE) and the Pfeffer's Questionnaire of Activities of Daily Living (PQADL). **Results:** A total of 65 seniors were evaluated. Most subjects were male (52.3%), married (58.5%), mean age 72 years and four (6.2%) were literate. Mean MMSE and PQADL were respectively 18.83 and 4.03 points. Among these, eight individual (12.3%) had cognitive impairment according to MMSE cutoff point proposed for illiterate individuals. In this sample, women had mean values of MMSE lower and PQAVD higher compared to men (CI=95%). **Conclusions:** Prevalence of cognitive disorder in this isolated African Brazilian community of descendent of slaves was lower than that observed in other Brazilian epidemiological studies with general population. Cultural aspects related to natural food, active exercises even among elderly and a rich cultural social life may explain the low prevalence of dementia and therefore account as a social-cultural factor intervenient in dementia etiology. This community is still invisible to any geriatric health public system, deserving more attention by government assistance.

**REPRESENTAÇÕES SOCIAIS, CULTURA E VELHICE: UM ESTUDO COMPARADO ENTRE AS CIDADES DE NÜRNBERG E MONTES CLAROS** *Marina da Cruz Silva*

**RESUMO.** O presente escrito discute os principais achados da pesquisa sobre as experiências do envelhecer para homens e mulheres velhas no Brasil (Montes Claros) e na Alemanha (Nürnberg), realizada no período compreendido entre 2008 e 2011. O principal objetivo da pesquisa foi investigar a importância da variável sociocultural e das condições socioeconômicas, aliada às categorias classe social, gênero, gerações e raça/etnia no que concerne à representação social da velhice numa perspectiva transcultural. Para tanto, adotou-se abordagem qualitativa e quantitativa (dados secundários sobre as condições de vida na velhice). Ao todo, foram entrevistados 60 pessoas, 30 em cada cidade estudada, sendo desses 15 homens e 15 mulheres acima de 60 anos. Os dados qualitativos revelam semelhanças e diferenças na forma de se conceber e vivenciar a velhice, demonstrando que homens e mulheres refletem e são reflexos das dimensões culturais, sociais e históricas do contexto, sendo notórias as conotações atribuídas às categorias de classe, gênero, etnia e geração no contexto de cada cidade estudada.

**REVERSÃO DO TRANSTORNO COGNITIVO LEVE POR MEIO ESTIMULAÇÃO E REABILITAÇÃO COGNITIVA MEDIADA POR COMPUTADORES E INTERNET, ESTUDO DE INTERVENÇÃO *QUASI* EXPERIMENTAL** *André Junqueira Xavier; Carolina Costa; Eleonora Zacchi; Gabriela Cavalieri; Marianne Briesemeister; Thaís Machado*

**RESUMO.** O Transtorno Cognitivo Leve (TCL) é a perda cognitiva clinicamente observável em relação à idade e escolaridade, mas com manutenção da autonomia e independência. O TCL possui maior risco para demência, porém, é reversível de 4,5% a 53%. A Oficina da Lembrança é um programa de Estimulação e Reabilitação Cognitiva (ERC) voltada para portadores de TCL. **Objetivo:** determinar o grau de reversão do TCL dos participantes do programa de ESR e avaliar a eficácia da intervenção. Métodos Estudo de coorte de intervenção controlado do tipo quasi experimental. Amostra de 40 participantes com 60 anos ou mais, independentes e autônomos, da comunidade, com queixas subjetivas de memória com MMSE inicial<26, não demenciados. O grupo intervenção contou com 20 oficinas bissemanais de uso de computadores e internet, 1,5 hora cada em laboratórios de computadores mais acompanhamento médico ambulatorial o grupo controle teve apenas acompanhamento médico ambulatorial. Modelo final obtido por meio de regressão de Cox, p<0,05. Foi considerado portador de TCL aquele que apresentou MMSE<26 e sem alteração funcional pelo escore BOMFAQ/OARS, coleta e acompanhamento feitos por alunos do internato de medicina e supervisionados por especialista. Aprovação CEP/UNISUL no 1663.07. **Resultados:** A amostra total de 40 pacientes, idade 66,2±5,2 anos, 7 homens, 13 controles. Na análise bivariada, além de pertencer ao grupo intervenção p=0,006, o sedentarismo p=0,033, IMC >27 p=0,040, doença cárdio ou cerebrovascular p=0,007, hipertensão arterial p=0,001 e dislipidemia p=0,014 foram significativos. No mode-lo multivariado apenas ser do grupo intervenção foi significativo para reversão do TCL (RR) de 6,62 (1,54-28,43; 95%IC) p=0,011. Análises controladas por idade, escolaridade, classe social, diabetes, exposição ao tabaco, depressão, hipotireoidismo, polimedicação, uso de benzodiazepínicos, MMSE inicial e tempo decorrido entre a primeira e segunda entrevistas. Do grupo controle 15,1% (n=2) reverteram, enquanto que no grupo tratado pela ERC 81,5% (n=22) reverteram (p=0,000), RAR=66% e NNT=15. Limitações: amostra pequena e não randomizado. Forças: acompanhamento clínico, ponto de corte de alta especificidade no MMSE para determinar pessoas sem alterações cognitivas, avaliação detalhada da capacidade funcional. **Conclusão:** Participar do programa de ERC por meio de computadores e internet teve associação independente com a reversão do quadro de TCL com grande impacto do tratamento.

### OUTROS

**USO DE MEDICAMENTOS EM IDOSOS RESIDENTES NA REGIÃO EXTREMO-OESTE DE SANTA CATARINA** *Vanessa da Silva Corralo; Clodoaldo Antônio de Sá; Clenise Liliane Schmidt; Karine Schwaab Brustolin; Marina Winckler*

**RESUMO.** O uso de medicamentos é uma das principais formas de controlar e prevenir as doenças crônicas mais prevalentes entre os idosos. Entretanto, com frequência isso predispõe a polimedicação ou polifarmácia, incidindo em maiores riscos de efeitos ad-versos e maiores índices de internações hospitalares relacionados aos casos de intoxicação. O propósito deste trabalho foi avaliar o consumo de medicamentos e as principais classes terapêuticas utilizadas por idosos de ambos os sexos residentes na Região Ex-tremo-Oeste de Santa Catarina. Foram entrevistados um total de 543 indivíduos, sendo 35% do sexo masculino (idade: 68,56±7,39 anos) e 65% do sexo feminino (idade: 68,15±7,18 anos), residentes na Região Extremo-Oeste de Santa Catarina. Todos os avaliados trouxeram, no dia da avaliação, todos os medicamentos que faziam uso. A análise dos dados demonstrou que 79,37% dos idosos entrevistados utilizam medicamentos, sendo que a prevalência foi maior no sexo feminino (87,54%) quando comparado ao sexo masculino (64,21%). A classe terapêutica mais utilizada entre os sujeitos do sexo masculino foi a dos antihipertensivos e diuréticos, sendo que os mais utilizados foram captopril (37,70%) e hidroclorotiazida (33,61%), seguido pela classe dos medicamentos que agem no sistema nervoso central (SNC), antidiabéticos, antiulcerosos, antiinflamatórios e hipolipemiantes. Da mesma forma, para o sexo feminino os antihipertensivos e diuréticos foram os medicamentos mais utilizados (captopril e hidroclorotiazida: 28,16% e 46,28%, respectivamente). Diferente do encontrado no sexo masculino, as mulheres utilizam mais fármacos que atuam no SNC e outras classes terapêuticas não citadas pelo sexo masculino. Cabe salientar o alto consumo de ácido acetilsalícilico entre os idosos e idosas (22,13% e 30,10%, respectivamente). O elevado consumo de medicamentos na população estudada constitui um fator preocupante devido a maior vulnerabilidade dos idosos frente aos efeitos adversos e as interações medicamentosas.

### PSICOLOGIA/NEUROPSICOLOGIA

**COGNITIVE PERFORMANCE IN OLDER ADULTS WITH POSITIVE OR NEGATIVE SELF-PERCEPTION OF HEALTH** *Camila Rosa de Oliveira; Cristiane Silva Esteves; Amanda Fernandes; Valéria Gonzatti; Luciane Scheufler; Juliana Colomby Ortiz; Irenio Gomes Filho; Tatiana Quarti Irigaray; Irani Iracema de Lima Argimon*

**ABSTRACT.** Self-perception of health is characterized as a subjective assessment that each individual makes about his health, encompassing physical, cognitive and emotional aspects. It is recognized as a useful and reliable for the evaluation and monitoring of quality of life and health status of the population. However, there are few studies in the Brazilian context to assess the self-perception of health in the elderly and their relationship to cognitive functioning. The aim of this study was to compare the cognitive performance of elderly according to self-perception of health (positive or negative). The sample consisted of 102 elderly from the southern region of the country, aged 60 and 77 years with different levels of education, divided according to the evaluation of self-perception of health: [1] good/excellent (n=71) and [2] bad/very bad (n=31). The elderly responded to a sociodemographic questionnaire, the Mini Mental State Examination (MMSE), the Geriatric Depression Scale of 15 points (GDS-15), the subtests Word List, Copying of Figures and Verbal Naming of the Consortium to Establish a Registry for Alzheimer's Disease (CERAD), tasks of verbal fluency (FAS and Animals) and the Logical Memory subtest of the Wechsler Memory Scale (WMS). We used descriptive analysis, Student t Test for independent sample and analysis of covariance, considering results significant at p≤0.05. The statistical package was SPSS 17. The groups did not differ in age, years of formal study and MMSE score. However, the group that assessed the self-perception of health as bad/ very bad had a mean score on the GDS-15 significantly higher. The GDS-15 score was covariate to compare the cognitive performance between groups due this difference. There were significant differences in performance between groups in scores on the WMS (immediate recall) and Verbal Naming of CERAD, demonstrating that the group with good/excellent self-perception of health obtained higher scores than the group bad/very bad. According to the results, we found that elderly people with positive self-perception of health showed better results in verbal memory skills (immediate recall) and verbal naming compared to the elderly with negative self-perception of health. As a continuation of the study it is suggested to increase the number of participants as well as the investigation of other physical and emotional factors that can influence the self-perception of health and cognitive performance in the elderly.

**COMPONDO O ENVELHECIMENTO SAUDÁVEL COM A INCLUSÃO DIGITAL** *Maria Suzana Souza; Camila Vieira Ribeiro; Vanessa Conceição do Nascimento*

**RESUMO.** O envelhecimento é um processo do desenvolvimento normal, envolvendo alterações neurobiológicas estruturais, funcionais e químicas. Incidem ainda, sobre o organismo fatores ambientais e socioculturais - como qualidade e estilo de vida fortemente ligados ao envelhecimento sadio ou patológico. Este estudo teórico tem como objetivo ressaltar a importância de compor o envelhecimento saudável através da inclusão digital, fundamentados em resultados recentes da pesquisa em neuropsicologia. Conclui-se que o aumento da idade não significa necessariamente adoecer; com medidas preventivas pode-se manter o idoso em condições saudáveis nos domínios cognitivos, físico e social, mantendo a autonomia de vida por extenso período. Porém, na presença de disfunções, o diagnóstico e a intervenção precoces podem propiciar uma melhor qualidade de vida ao paciente e sua família. Embora o ato de “envelhecer” traga consigo em definição, algo que perpasse a incapacidade dos idosos no que diz respeito às suas limitações, a tecnologia e seus adventos os auxilia cada vez mais a estarem inseridos na sociedade, ainda que as atualizações diárias e as novidades tecnológicas sejam dominadas, em sua maioria, por crianças e jovens (Kachar, 2010). Barbosa, Lempke e Mota (2012), afirmam que algumas funções tendem a ficar preservadas, como a inteligência cristalizada, as habilidades motoras, a memória implícita e o aprendizado não-associativo, todavia, outras funções tendem a declinar com a idade, como as habilidades visoespaciais, a memória explicita e de trabalho, as funções atentivas e executivas. Segundo Banhato, Guedes, Magalhães, Mota, Scoralick e Silva (2007), ainda citam que a inclusão digital pode estimular as atividades mentais, promovendo a preservação das habilidades emocionais e cognitivas. Além disso, a interação com outras pessoas da mesma idade, em sala de aula, auxilia o convívio social, o que nesta fase da vida, muitas vezes está prejudicada. Desse modo, a partir da definição de envelhecimento, pode-se destacar que, embora haja interferência no desempenho de determinadas habilidades cognitivas, a interação da terceira idade com a tecnologia auxilia no “envelhecer saudável”. Isso torna essa fase da vida mais leve, o que motiva o idoso a se apresentar de modo positivo para a sociedade (Kachar, 2010).

**EFEITOS COGNITIVOS DA ADMINISTRAÇÃO ORAL DE D-SERINA EM IDOSOS SAUDÁVEIS** *Marcos Avellar; Caroline Madeira; C. Vargas -Lopes; Pedro Henrique Siqueira; Raphaela Machado; Camila Dantas; Priscila do Nascimento; Christiane Miranda; Alex Manhães; Homero Leite; Rogério Panizzutti*

**RESUMO.** Com o crescimento da população mundial de pessoas idosas, o declínio cognitivo associado ao envelhecimento está assumindo importância crescente. A D-serina é um aminoácido endógeno que participa da ativação dos receptores de glutamato do subtipo NMDA no cérebro, que são fundamentais para diversas funções cognitivas. Evidências indicam que uma redução na disponibilidade de D-serina contribui para o declínio cognitivo associado ao envelhecimento. Neste estudo avaliamos o efeito da administração oral de D-serina sobre a cognição de 50 idosos saudáveis, em um protocolo controlado, cruzado e duplo-cego. A cognição foi avaliada por testes computadorizados que avaliaram: atenção, memória de trabalho, função executiva e resolução de problemas espaciais. No início do estudo coletamos amostras de sangue e analisamos o conteúdo de D-serina. Observamos uma redução nos níveis plasmáticos de D-serina com o aumento da idade (r=0,34, p=0,03), confirmando nesta população idosa os resultados que observamos previamente em adultos jovens saudáveis. A administração de D-serina melhorou os testes de função executiva e de resolução de problemas espaciais. Especificamente, a D-serina reduziu significativamente os erros no teste de discriminação visual reverso da tarefa Set-Shifting (W= -437, p=0,03), e na segunda (W= -384, p=0,05) e quinta rodadas do teste do labirinto de Groton (W= -1069, p<0,0001). D-serina não teve efeito significativo sobre os resultados dos testes de atenção e de memória de trabalho. Em conjunto, estes resultados indicam que a administração de D-serina em idosos pode melhorar aspectos da função executiva e da resolução de problemas espaciais nesta população. Sugerimos que a administração de D-serina é uma estratégia para a remediação de aspectos do declínio cognitivo associado ao envelhecimento.

**PERFIL DE FUNCIONAMENTO COGNITIVO EM PACIENTES IDOSOS HIV POSITIVOS** *Pedro Henrique Pinto; Leonardo Ferreira Caixeta; Carlos Alberto Pinto; Emanuela Torreão Brito e Silva*

**RESUMO.** Problemas cognitivos em hospitais de doenças infecciosas são pouco abordados, o que implica em subdiagnóstico de condições como a demência por HIV e outros transtornos cognitivos relativos ao HIV, principalmente nas populações de idosos que inspiram outro diagnóstico de demência. Objetivamos reportar a avaliação neuropsicológica numa população idosa HIV positiva na região central do Brasil a fim de melhor caracterizar perfis de funcionamento neurocognitivo. Idosos soropositivos confirmados para HIV, admitidos ao Hospital de Doenças Tropicais (HDT), no periodo de um ano, foram avaliados usando baterias neuropsicológicas padronizadas, capazes de cobrir todos os domínios cognitivos, acrescidos de medidas clínicas, como avaliação funcional e sintomas depressivos. Os pacientes foram comparados em relação a medidas neuropsicológicas. Os procedimentos neuropsicológicos tiveram sucesso em detectar deficits cognitivos na maioria dos pacientes, tendo em vista variações entre si relativas a parâmetros laboratoriais (contagem CD4, glicemia, trigliceridemia e HAS) e estado nutricional, em que se verificou variabilidade nos resultados neurocognitivos. Mais de 50% apresentou sintomas cognitivos e um quarto apresentou um quadro clínico completo de demência com peculiaridades frontosubcorticais (em especial, atenção e disfunção executiva associadas a apatia e bradicinesia). A aplicação da avaliação neuropsicológica em doenças infecciosas, especialmente aquelas que acometem o SNC, pode trazer importantes insights ao infectologista ou médico assistente no que tange ao esclarecimento etiológico da doença, fisiopatologia, diagnóstico diferencial e fatores clínicos concorrentes que podem influenciar o status cognitivo.

**PERFIL DO PREJUÍZO COGNITIVO DE IDOSOS COM DOENÇA ARTERIAL PERIFÉRICA ASSOCIADO A DÉFICITS EXECUTIVOS E DE LINGUAGEM: UM ESTUDO NEUROPSICOLÓGICO** *Naomi Vidal Ferreira; Rita de Cássia Gengo e Silva; Paulo Januzzi Cunha; Cristiano Teixeira Mostarda; Fernanda Consolim Colombo; Maria Claudia Irigoyen; Danielle Irigoyen da Costa*

**RESUMO.** As funções cognitivas de idosos com doença arterial periférica (DAP) podem estar comprometidas, devido ao prejuízo da circulação sistêmica. Não se sabe ao certo, no entanto, o perfil de comprometimento cognitivo desses pacientes. **Objetivo:** Avaliar as funções cognitivas de idosos com DAP e compará-las às funções cognitivas de idosos saudáveis, para identificar os prejuízos.

**Método:** Foram avaliados 25 idosos com DAP e 47 idosos saudáveis (C). Os participantes foram submetidos ao exame do Índice Tornozelo Braquial (ITB) e a uma avaliação neuropsicológica composta pelos instrumentos: Memória Lógica I e II e Reprodução Visual I e II (WMS-R); Dígitos (WAIS); Stroop Color Word Test (SCWT); Fluência Verbal Semântica (Animais) e Fonêmica (Letras - FAS); Desenho do Relógio; MMSE. **Resultados:** Os grupos apresentaram semelhanças quanto à idade (C=68,13±8,48 vs DAP=67,88±8,32, p>0,05), IMC (C=25,93±4,46 vs DAP=25,45±4,73, p>0,05), PAS (C=136,23±20,43 vs DAP=146,19±22,69, p>0,05), PAD (C=80,11 ±10,62 vs DAP=79,71±12,56, p>0,05), e FC (C=65,82±13,07 vs DAP=64,40±10,45, p>0,05), e diferenças em relação à escolaridade (C=12,73±5,07 vs DAP=7,88±4,72, p<0,05) e ITB (C=1,08±0,08 vs DAP=0,60±0,18, p<0,05). Quanto à cognição, o grupo DAP apresentou comprometimento da atenção (C=10,89±3,28 vs DAP=8,55±1,82, p<0,05), da fluência verbal fonêmica (C=34,52±15,41 vs DAP=26,95±10,44, p<0,05) e da fluência verbal semântica (C=18,52±7,41 vs DAP=15,82±3,51, p<0,05). Não houve diferença significativa na memória visual imediata (C=29,93+5,55 vs DAP=28,28±4,76, p>0,05) e tardia (C=25,11±8,97 vs DAP=23,61±5,99, p>0,05), memória verbal imediata (C=24,02±7,18 vs DAP=21,78±7,24, p>0,05) e praxia construtiva (C=8,47±1,79 vs DAP=8,00±2,11, p>0,05). Quanto à memória verbal tardia (C=18,57±7,19 vs DAP=15,04±7,23, p=0,06), velocidade de processamento (C=23,59s±9,28 vs DAP=30,36s±14,70, p<0,05) e MMSE (C=28,34±1,61 vs DAP= 27,54±1,69, p=0,06), houve apenas tendência. **Conclusão:** Os idosos com DAP apresentaram comprometimento da atenção, fluência verbal fonêmica e fluência verbal semântica, mas não da memória visual, memória verbal e praxia construtiva, sugerindo disfunção executiva e de linguagem, provavelmente por prejuízos pré-frontais. A diferença de escolaridade deve ser investigada, por poder representar tanto um prejuízo funcional quanto anteceder às alterações verificadas. Estudos prospectivos e com neuroimagem podem esclarecer a relação entre DAP, envelhecimento e funções cognitivas.

**QUALIDADE DE VIDA DE CUIDADORES DE PESSOAS COM DEMÊNCIA LEVE E MODERADA E SUA RELAÇÃO COM CARACTERÍSTICAS SOCIODEMOGRÁFICAS E CLÍNICAS DE CUIDADORES E PACIENTES** *Marcia Cristina Nascimento Dourado; Raquel Luiza Santos; Maria Fernanda Barroso de Sousa; José Pedro Simões; Tatiana Belfort; Bianca Torres Mendonça de Melo; Rachel Dias*

**RESUMO.** **Objetivos:** Investigar os fatores relacionados à qualidade de vida (QdV) de cuidadores de demência leve e moderada. **Métodos:** Cuidadores (n=88) estratificados pela gravidade dos pacientes. Avaliamos QdV, sobrecarga, sintomas de depressão e ansiedade dos cuidadores, e gravidade, cognição, funcionalidade, sintomas neuropsiquiátricos, consciência da doença e depressão dos pacientes (n=88). **Resultados:** Não houve diferença significativa na QdV de cuidadores de demência leve e moderada. A regressão demonstrou sobrecarga (p<0,05) e sintomas depressivos (p<0,001) como principais fatores relacionados à QdV dos cuidadores. Sintomas depressivos dos cuidadores (p<0,001) e sintomas neuropsiquiátricos dos pacientes (p<0,001) foram relacionados à sobrecarga de cuidadores de demência leve. Ansiedade de cuidadores e pacientes(p<0,01) e QdV (p<0,01) dos cuidadores foram relacionados à sobrecarga de cuidadores de demência moderada. Sintomas depressivos dos cuidadores de demência leve foram relacionados à atividade motor aberrante (p<0,001) e ansiedade (p<0,001) dos pacientes e aos domínios “amigos” (p<0,001) e “humor” (p<0,05) da QdV dos cuidadores. Sintomas depressivos de cuidadores de demência moderada foram relacionados aos sintomas de ansiedade (p<0,001) e à QdV global (p<0.001) dos cuidadores. **Conclusões:** Embora não haja diferença na QdV de cuidadores pela gravidade da demência, nossos achados sugerem que depressão e sobrecarga de cuidadores de demência leve e moderada estão relacionadas a diferentes fatores. Este estudo possui implicações nas intervenções clínicas.

**SYMPTOMS OF POSTTRAUMATIC STRESS DISORDER AND COGNITIVE FUNCTIONING IN THE ELDERLY** *Tatiana Quarti Irigaray; Silvana Kessler de Oliveira Corrêa Oliveira; Rodrigo Grassi-Oliveira; Rochele Paz Fonseca; Irani Iracema de Lima Argimon; Christian Haag Kristensen*

**ABSTRACT. Introduction:** Posttraumatic Stress Disorder (PTSD) has been associated with neurobiological, structural, and functional impairments in the brain. However, there are controversial results about which cognitive functions are impaired because of the trauma. It is estimated that 60 to 90% of the general population is exposed to at least one stressor that may be potentially traumatic in their lives. **Objective:** To investigate the correlation between current symptoms of PTSD and cognitive functioning in the elderly. **Method:** The study included 124 elderly, aged 60-94 years (M=68.52, SD=9.32). Symptoms of PTSD were investigated using the Screen for Posttraumatic Stress Symptoms (SPTSS) and cognitive functioning was assessed using the Mini-Mental State Examination (MMSE), the D2 Test, the California Verbal Learning Test (adapted), and the “F” Phonemic Fluency Test (adapted). The associations between the PTSD symptoms on the SPTSS and the cognitive variables were measured using Pearson‘s correlation coefficient. **Results:** There was a moderate negative correlation between the total score on the SPTSS and the total number of correct answers in the California Verbal Learning Test (p≤0.001). We found weak but significant negative correlations between the total score on the SPTSS and the total number of words starting with the letter “F” evoked (p=0.008) and the MMSE total score (p=0.003). **Conclusion:** The present study suggests that symptoms of posttraumatic stress are associated with poorer performance in terms of declarative episodic-semantic memory, verbal initiation, inhibition and planning with phonemic restriction and global index of cognitive functioning in the elderly. These findings underscore the need for further studies aiming at an early identification of traumatic events and their associated cognitive changes in the elderly, directly leading to the improvement of intervention planning with more specific and long lasting results.

**THE S-TOFHLA AS A MEASURE OF FUNCTIONAL LITERACY IN PATIENTS WITH MILD ALZHEIMER'S DISEASE OR MILD COGNITIVE IMPAIRMENT** *Maira Okada de Oliveira; Ricardo Nitrini; Sonia Maria Dozzi Brucki*

**ABSTRACT.** In developing countries education levels vary dramatically, especially among the elderly. To be able to accurately assess patient cognition, clinicians must take the level of schooling into account. In some developing countries, such as Brazil, however, the number of years of schooling does not always correlate with the true level of educational competency. This study was designed to verify the accuracy of the Short-Test of Functional Health Literacy in Adults (S-TOFHLA) in individuals with mild cognitive impairment (MCI) and mild Alzheimer's Disease (AD), as compared with healthy controls (HC), in order to assess its utility as a measure of functional literacy. We performed an observational, cross-sectional and descriptive study structured as an interview with a neuropsychologist that included basic demographic information, socioeconomic status and co-morbidity, a health literacy assessment and a cognitive battery in order to measure overall cognitive status, crystallized and fluid intelligence, memory, visuoconstructive task aptitude and to estimate IQ. One hundred forty-eight subjects were divided into three groups: HC (n=61), MCI patients (n=42) and AD patients (n=45). Health literacy was assessed with the S-TOFHLA. Cognitive evaluation was assessed by Brief Cognitive Screening Battery (learning and recall of drawings), Dementia Rating Scale, Rey Auditory Verbal Learning Test, Raven's Colored Matrices, Clock Drawing, Verbal Fluency and estimated IQ, calculated by adding the weighted scores of subtests block design and vocabulary on the Wechsler Adult Intelligence Scale-III (WAIS-III). While the S-TOFHLA was not useful in measuring literacy in patients with AD, it proved to be adequate in assessing scores when the control group was compared with patients with MCI. In S-TOFHLA, nonparametric multiple comparisons indicated that a statistically significant difference in reading comprehension and the overall score occurred among the three groups (HC, MCI and AD). In numeracy, the statistical difference occurred only between the AD and HC groups. The S-TOFHLA does not seem to be suitable as an instrument to measure functional literacy for patients with advanced cognitive impairment, but proved to be appropriate in both the control group and MCI patients in numeracy and prove to be useful as an adjuvant to estimate IQ, reading ability and premorbid IQ, as an indicator of cognitive reserve.

### PSIQUIATRIA

**IS THE ASSOCIATION BETWEEN DEPRESSION AND SELF-REPORTED PARKINSON'S DISEASE INFLUENCED BY COGNITIVE IMPAIRMENT? EVIDENCE FROM A POPULATION-BASED SURVEY** *Marcos Hortes N. Chagas; Tais S. Moriyama; Rodrigo A. Bressan; André C. Felício; Ana Luisa Sosa; Cleusa P. Ferri*

**ABSTRACT. Introduction:** Depression and dementia are common non-motor manifestations of Parkinson's disease (PD). Prevalence estimates vary widely, but most studies have suggested that 40% to 50% of PD patients present clinically relevant depression and 30% of them present dementia. **Objectives:** To test the hypothesis that severity of cognitive impairment modifies the effect size of the association between depression and self reported Parkinson's disease. Design: One-phase population-based door-to-door surveys. Setting: Middle-income countries (India, China, Cuba, Dominican Republic, Venezuela, Mexico, and Peru). Participants: 14,960 residents from defined catchment areas aged 65 years and older. This study is restricted to those with cognitive impairment (n=1,474). Measurements: Subjects were assessed for depressive symptoms with the Geriatric Mental State and diagnosis of depression determined according to ICD-10. PD diagnosis was determined through self-report. The 32 item cognitive test Community Screening Instrument for Dementia was used to measure cognitive status. Disability was evaluated with the World Health Organization Disability Assessment Schedule II. **Results:** The mean age was 79.3 years old and most (69%) were women. Depression was significantly higher among participants with self-reported PD (adjusted OR 2.19, 95%CI 1.08-4.45). Despite the fact that the test for interaction was not statistically significant (p=0.28), there was a clear increase on the ORs of the association between depression and self-reported PD with decreased scores in the cognitive test (from 0.98 (0.24-4.02) in the highest quartile to 8.04 (1.57-41.15) the lowest quartile) **Conclusion:** We show that the strength of the association between depression and self-reported PD increases with the severity of the cognitive impairment. Prevalence of depression among people with PD might be underestimated if people with impaired cognition are excluded

### TERAPIA OCUPACIONAL

**PERFIL DE PESSOAS IDOSAS ATENDIDAS EM UM CENTRO DE ATENÇÃO PSICOSSOCIAL E SUA VISÃO SOBRE OS ATENDIMENTOS OFERECIDOS NA INSTITUIÇÃO** *Tiago Rodrigo Biasoli; Leonardo José Costa de Lima*

**RESUMO.** A maior quantidade de pessoas idosas a nível mundial, apesar de ser uma conquista traz novos desafios á sociedade, como a demanda por serviços de saúde tanto na Rede Básica como no Atendimento Especializado, incluindo os Centros de Atenção Psicossocial, CAPS. Considerando um aumento significativo dos usuários com idade superior a 60 anos atendidos por este serviço, esta pesquisa, com lócus qualitativo descritivo, baseado em dados secundários do acervo documental e de entrevistas semi estruturadas, teve como o objetivo principal a caracterização dessa população, com o intuito de identificar a realidade desses usuários quanto a sua situação pessoal, social, de tratamento e da forma pela qual eles compreendem a atuação da Terapia Ocupacional na instituição e, secundariamente, verificar se os tratamentos empregados na unidade abrangem também as características e necessidades do processo de envelhecimento, conforme relatado na literatura. A amostra foi composta por 36 usuários com idade superior a 60 anos, que realizam tratamento no CAPS Esperança, localizado na cidade de Campinas, que correspondem a 12,2% de todos os atendidos no referido serviço. Esses indivíduos caracterizaram-se por serem predominantemente do sexo feminino; a média de idade foi 66 anos; com baixo nível de escolaridade; aposentados e solteiros. O diagnóstico mais frequente foi de esquizofrenia 52,7%, sendo que grande parte desses sujeitos foram inseridos no serviço já sexta década de vida (53%). Apesar de morarem sozinhos e apresentarem outras comorbidades, a maioria não conta com o auxílio de um cuidador informal. Estes dados retratam o crescente aumento da população idosa com transtornos mentais que consequentemente gera uma elevação da inserção desses indivíduos em equipamentos de saúde mental, tais como o CAPS. A importância do levantamento do perfil dessa população está no fato de que os fatores relacionados ao processo de envelhecimento deveriam influenciar o planejamento e implantação desses serviços, pois devido a suas características peculiares também é frequente o subdiagnóstico e consequentemente um subtratamento. Descritores: Saúde do Idoso, Saúde Mental, CAPS, Terapia Ocupacional, Envelhecimento.

### FISIOTERAPIA

**EFEITO DO EXERCÍCIO DE FORTALECIMENTO MUSCULAR E AERÓBICO SOBRE OS NÍVEIS PLASMÁTICOS DE BDNF E SINTOMAS DEPRESSIVOS EM IDOSAS** *Daniele Sirineu Pereira; Bárbara Zille de Queiroz; Diogo Carvalho Felício; Natália Reinaldo Sampaio; Danielle Aparecida Gomes Pereira; Antonio Lucio Teixeira; Leani Souza Máximo Pereira*

**RESUMO.** Com o envelhecimento ocorre uma diminuição dos níveis de fatores neurotróficos, o que pode contribuir para o risco de depressão em idosos. A depressão é um dos transtornos psiquiátricos mais comuns na população idosa, sendo responsável pela perda de autonomia e agravamento de quadros patológicos preexistentes. Estudos relataram uma redução significativa dos níveis do fator neurotrófico derivado do cérebro (BDNF) em idosos deprimidos. O exercício físico apresenta efeitos benéficos sobre a depressão e induz um aumento dos níveis de BDNF. Entretanto, pouco é conhecido sobre o padrão de produção do BDNF em resposta ao exercício no idoso. **Objetivo:** investigar o efeito de dois programas de exercícios físicos, fortalecimento muscular (EFM) e aeróbico (EA), sobre os níveis plasmáticos de BDNF e sintomas depressivos em idosas da comunidade. **Métodos:** Este estudo faz parte de um ensaio clínico (ReBEC:RBR9v9cwf), onde participaram do 451 idosas comunitárias. As concentrações plasmáticas de BDNF foram mensuradas pelo método de ELISA. A Escala de Depressão Geriátrica (GDS) foi usada para rastreamento de transtornos depressivos, versão com 15 itens, sendo adotados como pontos de corte 5/6 para não caso/ caso. ANOVA two-way foi usada para investigar o efeito do treinamento nas mudanças nos níveis plasmáticos de BDNF, escores e classificação caso/não caso na GDS e identificar diferenças entre os grupos EFM e EA (p<0,05). (COEP: ETIC 038/2010). **Resultados:** Quanto aos sintomas depressivos, 24% e 19,5% das idosas foram consideradas casos nos grupos EFM e EA respectivamente (p=0,252). O EFM aumentou significativamente os níveis plasmáticos de BDNF. Contudo, não houve diferença nas dosagens de BDNF após o EA. Houve uma diminuição dos escores da GDS, assim como na classificação caso/ não caso para depressão após o treinamento para ambos os programas de exercício físico. **Conclusão:** Na presente amostra, tanto

o EFM quanto o EA apresentaram um efeito positivo sobre os sintomas depressivos em idosas da comunidade. Entretanto, o efeito do exercício físico sobre os sintomas depressivos possivelmente não foram influenciados pela ação do BDNF, uma vez que apenas o EFM aumentou os níveis de BDNF. Em conjunto com informações atualmente disponíveis, os resultados do presente estudo sugerem um padrão diferenciado de produção e liberação do BDNF na população idosa quando comparado aos indivíduos jovens. Apoio: CNPq, CAPES, FAPEMIG.

### GENÉTICA

**CORRELATION BETWEEN ATTENTION AND MEMORY AND PHENOTYPIC VARIATION OF BUTYRYLCHOLINESTERASE IN PATIENTS WITH ALZHEIMER'S DISEASE** *Gleyse Freire Bono; Daiane Priscila Simão Silva; Meire Silva Batistela; Nalini Drieli Josviak; Leandro Ribeiro Cordeiro; Mauro Roberto Piovezan; Ricardo Krause Martinez de Souza; Lupe Furtado Alle*

**ABSTRACT.** Alzheimer's disease (AD) is a neurodegenerative disorder in which there is a decline of cholinergic function. The human butyrylcholinesterase (BChE) is an enzyme encoded by BCHE gene (3q26.1-q26.2) that has been associated with cognitive decline in AD. The interaction between the products of BCHE gene and CHE2 locus results in a complex named C5 band identified in electrophoresis. The CHE2 C5+ and CHE2 C5- phenotypes correspond to the presence and absence of the band, respectively. BChE activity is influenced by several factors, including CHE2 C5+ phenotype which is related to increased BChE activity (≅ 30%) and also treatment with cholinesterase inhibitors (ChEIs). Studies have reported that treatment with ChEIs improve cognition, particularly regarding attention because memory consolidation seems to require low cortical cholinergic activity. The aim of this study was to identify the CHE2 loco phenotypes and correlate them with the preservation of attention and memory, through the analysis of the mini-mental state examination (MMSE) in subjects CHE2 C5+ and CHE2 C5- in a sample of 79 patients diagnosed according to the criteria NIA-AA 2010 for probable AD. In the present study, we also evaluated the preservation of attention and memory in patients CHE2 C5- (n=44) treated and not treated with ChEIs. Phenotypic frequencies of CHE2 C5+ was 10.13%, similar to that previously described for the population of Curitiba (10.26%). Patients with CHE2 C5- phenotype showed greater preservation of attention (47.16%) in comparison with patients CHE2 C5+ phenotype (34.37%). Patients CHE2 C5- treated with ChEIs showed better preservation of memory and attention (31.53% and 8.3% respectively) compared to those without the use of ChEIs (14.8% and 3% respectively). This result corroborates the expected effect of these drugs on maintaining or improving the cognitive control of attention in patients DA. The fact that individuals with CHE2 C5- phenotype showed higher preservation of memory and attention than those with CHE2 C5+ phenotype can be explained by the increased BChE activity that the CHE2 C5+ phenotype confers which may act on cholinergic transmission by decreasing extracellular ACh levels. Taken together, these results highlight the importance of the pharmacogenetic analysis, as the loco CHE2, for the indication of the drug and evaluation of cognitive domains for which the ChEIs are indicated.

**GENETIC INFLUENCES ON MEMORY SCORES IN AGING: INTERACTION BETWEEN GENDER AND POLYMORPHISM TAQIA IN GENEDRD2/ ANKK1** *Cláudia Justin Blehm; Camila Korb; Daiani de Fatima Pi-res da Silva Bamberg; Luciana Alves Tisser; Fabiana Michelsen de Andrade*

**ABSTRACT.** Memory is considered one of the most important of all cognitive functions. With aging, there is a naturally decrease in memory performance, even in the absence of dementia, or any other clinical condition that may be related. This decreasingis multifactorial, and one of the candidate genes is DRD2, which encodes subtype D2 of dopamine receptors. The polymorphism in this gene classically named TaqIA (rs1800497), is a SNP originally described in the 3‘ region of the DRD2, whose location has been discovered also correspond to the ANKK1 gene. This SNP refers to the exchange of cytosine by thymine bases (C/T, corresponding to the A2/A1 alleles), leading to Glu713Lys substitution in the gene exon8 ANKK1. This study aims to analyze the interaction between this polymorphism and gender on memory scores in volunteersstarting from 50 years without any kind of dementia declared. For memory scores determination were used Wechsler tests forlogical and visual memories, both immediate and delayed. Verbal learning ability was tested through Rey Auditory Verbal Learning Test. The initial sample of 367 volunteers was decreased after exclusion criteria application (use of psychotropic medication, or owned estimated IQ below 70, anxiety, depression or stress, assessed by psychological tests). The analysis of the variant investigated is being done by PCR-RFLP with genotypes available for 193 volunteers so far (mean age 63.6±7.8 years, 24.3% men). Using ANCOVA, memory scores were adjusted for gender and education level, and compared between genotypes. For the analysis of interaction with gender, an interaction term gender x gene was inserted, and the scores adjusted only by level of education.To date 44.5% of the volunteers had the genotype A2/A2, 4.2% was A1/A1 and 32.4% were heterozygous. As single variable, DRD2 was not significantly associated with any memory score. However, interactions with gender were detected: men with A2/A2 genotype had a mean visual memory immediate superior compared to individuals carrying the A1 allele, whereas women with A2/A2 genotype had an average lower than A1 allele carriers (p=0.07). The same interaction was observed for delayed visual memory, and it was much more pronounced (p=0.007). Our data demonstrate the modulation of genetic influences on memory by the hormonal status. The study is ongoing, which could bring new results for other types of memory.

**INTERACTION BETWEEN ACE INSERTION/DELETION POLYMORPHISM AND THE DIVERSITY OF DAILY ACTIVITIES ABOUT MEMORY SCORES IN AGING** *Camila Korb; Cláudia Justin Blehm; Daiani de Fatima Pires da Silva Bamberg; Luciana Alves Tisser; Fabiana Michelsen de Andrade*

**ABSTRACT.** The renin angiotensin system has been studied regarding its role on brain, and could have interference in memory modulation. The key element of this system is the angiotensin converting enzyme (ACE), and an insertion/deletion polymorphism in the gene that encodes this enzyme might alter its activity, therefore influencing memory. Besides, the mental stimulation through different activities types during life could also beneficially influence memory. The present study examined the effects of ACE gene insertion/deletion polymorphism on memory, together with the diversity of daily activities on memory scores. Volunteers had a minimum age of 50 years, and no diagnosis of neurological disease. Five memory classes were tested, using the Wechsler Memory Scale-Revised and The Rey Auditory-Verbal Learning Test. Initially 367 volunteers were tested, but 123 were excluded for using any kind of psychotropic medication, or owned estimated IQ below 70, anxiety, depression or stress, assessed by psychological tests. This exclusion criteria totalized 244 participants, and 157 of them were already genotyped for ACE variant (mean age of 62.7±7.6 years, and 26.8% of men). The genetic analyze was done by PCR. The diversity of daily activities was evalued through a questionnaire about the practice of 25 differents activities, asked both in current period and also before 40 years, through which each volunteer received a score 0 or 1, indicating the proportion of the total activities practiced on the total activities searched. The interaction analysis was accomplished through multiple linear regression, where were inserted the genotype variant for ACE (del/del versus ins allele carriers), the diversity activities scores, besides the interaction term between these variants. Significant interactions were interpreted using the linear regression equation. All analyses were performed using the software SPSS 20.0. Regarding the ACE gene variant, 28% of the sample was del/del, 22.3% was ins/ins, and 49.7% were heterozygotes. In regression models, homozygotes for the ins allele were grouped with heterozygotes. It was possible to detect a trend of interaction between ACE genotype and diversity of activities in the current period (p=0.079), demonstrating that the beneficial influence of the diversity of activities is more pronounced in carriers of del/del genotype. No other significant interaction was detected, but the project is ongoing, and new influences can still be found.

**DIGITAL LITERACY CAN REDUCE COGNITIVE DECLINE AND ATTENUATE HEALTH INEQUALITIES: FINDINGS FROM THE ENGLISH LONGITUDINAL STUDY OF AGEING (ELSA)** *Andre Junqueira Xavier; Eleonora d'Orsi; Cesar de Oliveira; Martin Orrell; Panayotes Demakakos; Jane Biddulph; Michael G Marmot*

**ABSTRACT. Introduction:** Cognitive decline is a major risk factor for disability, dementia and death. Digital Literacy (DL) is a pervasive social phenomenon that requires new skills and supports many activities. Previous studies indicate that stimulating mental activities may lead to a slower rate of cognitive decline. This work aims to study whether DL can reduce cognitive decline and inequalities in health. **Methods:** The study included 6442 participants aged 50 to 89 years followed up for 8 years from the English Longitudinal Study of Ageing (ELSA). DL (current user of internet/email, no user, new user or stopped using) was investigated as a predictor of decline in immediate recall, delayed recall and verbal fluency. Unadjusted and adjusted beta regression coefficients and their 95% confidence intervals were calculated through Generalized Estimating Equations, controlling for age, gender, wealth, education, physical disability, diabetes, cardiovascular diseases, baseline cognitive function, depressive symptoms and time. **Results:** Those who had never used or stopped using internet showed a greater decline of -7.12% (CI95%: -7.69 to -6.55), those who were new users of internet/email had stability and those who were current users of internet/email had an increase in delayed recall of 1.58% (CI95%: 1.06 to 2.10), the difference between the first and the last group was almost 9% over the follow up. Also, the higher on the wealth quartile (wealthier group), the lower the decline. The use of internet/email influences this gradient, the poorer groups who reported to be new users of internet/email, had a lower decline than the higher wealth groups who reported never using internet/email. This effect was stronger in the older group (65-89 years old). In the 65-89 years age group this effect was accentuated for the poorest groups so they were comparable with the higher wealth quartiles. This pattern persisted after stratification by physical disability. Participants who were current users of internet/email during the follow-up had better performance in all 3 cognitive outcomes relative to those who were not users. Lower wealth, age, diabetes, physical disability, lower previous cognitive function and lower educational level worsened cognitive outcomes. **Conclusions:** Digital Literacy is likely to reduce cognitive decline and attenuate inequalities in health.

## Poster Session

### ENFERMAGEM

**A VIVÊNCIA DO CUIDADOR INFORMAL PRIMÁRIO NO CUIDADO A IDOSOS PORTADORES DE DOENÇA DE ALZHEIMER** *Flávio Augusto dos Santos; Gislaine Desani da Costa; Rosely Almeida Souza*

**RESUMO.** A doença de Alzheimer pode ser definida como uma doença neurodegenerativa que leva ao declínio das funções intelectuais, alterações de comportamento e personalidade, reduzindo a capacidade de convívio social. Inicialmente, o paciente sofre perdas da memória recente, mas a memória remota mantém-se preservada. Com a evolução da doença, há comprometimento da capacidade de aprendizado, atenção, orientação, compreensão e linguagem e seu portador torna-se cada vez mais dependente de auxílio para a realização de suas atividades da vida diária, até mesmo para rotinas básicas. Além do mais, ocorrem os períodos de agressividade, depressão, desconfiança, e até mesmo paranoias, tornando o cuidado uma tarefa nada fácil para os familiares. Realizou-se uma investigação qualitativa, que teve como objetivo compreender a vivência do cuidador informal primário no cuidado a idosos portadores da doença de Alzheimer. A coleta de dados deu-se por meio de entrevista semi-estruturada, previamente agendada, e gravada, em uma associação de atendimento a idosos portadores da doença de Alzheimer localizada no município de São Paulo. Para a análise dos discursos utilizou-se a técnica de análise de conteúdo proposta por Bardin. Participaram do estudo 7 cuidadores, todos do sexo feminino (2 filhas e 5 esposas), entre 52 e 62 anos. A análise do material obtido dos depoimentos resultou em duas categorias empíricas: a convivência com a sintomatologia, com as subcategorias a doença e as emoções do cuidador; a convivência com as limitações, com as subcategorias limitações do paciente e limitações do familiar cuidador. A vivência do cuidador é carregada de situações estressantes e desgastantes, pois referem não saber como agir nos períodos de agitação, teimosia e agressividade, são sobrecarregados pelas limitações apresentadas pelo doente e ainda enfrentam dificuldades financeiras. Houve relatos de exaustão e tristeza. Considera-se que a doença de Alzheimer é uma doença familiar, portanto o enfermeiro deve assumir o papel de educador permitindo maior acessibilidade de informação no que se refere à doença de Alzheimer e principalmente elaborando formas de intervenção para melhor qualidade de vida dos cuidadores familiares.

**A VIVÊNCIA ENFERMEIROS E TÉCNICOS DE ENFERMAGEM NO CUIDADO A IDOSOS PORTADORES DE DOENÇA DE ALZHEIMER** *Vanusa Maria dos Santos; Gislaine Desani da Costa*

**RESUMO.** A doença de Alzheimer (DA) é uma doença neurológica degenerativa, irreversível e progressiva que acomete particularmente indivíduos da terceira idade. Seu início é insidioso caracterizando-se por perdas graduais da função cognitiva e distúrbios comportamentais e afetivos. Nos estágios iniciais da doença ocorrem o esquecimento e a perda da memória sutil, bem como, alterações da personalidade. Com a evolução da doença, o idoso vivencia uma situação de dependência total que requer cuidados cada vez mais complexos, ocasionando uma sobrecarga física e psíquica ao cuidador, levando-o a uma má qualidade de vida. Tais dificuldades faz com que os familiares procurem a ajuda de profissionais de saúde, também caracterizado como cuidador formal, para auxiliá-los no cuidado a esse doente. Realizou-se uma investigação qualitativa, que teve como objetivo compreender a vivência de enfermeiros e técnicos de enfermagem no cuidado a idosos portadores de DA. A coleta de dados deu-se por meio de entrevista semi-estruturada, previamente agendada, com uso de gravador, em uma Instituição de Home Care localizada na zona norte do município de São Paulo. Para a análise dos discursos utilizou-se a técnica de análise de conteúdo proposta por Bardin. Participaram do estudo 9 enfermeiros e 11 técnicos de enfermagem, sendo todos do sexo feminino e com faixa etária entre 25 e 41 anos. A partir dos depoimentos obtidos e da exploração do material, emergiram as seguintes categorias empíricas: a convivência do familiar com o idoso portador de DA sob a óptica do cuidador formal; as dificuldades vivenciadas pelos familiares sob a óptica do cuidador formal; as barreiras perante a adesão ao tratamento farmacológico; a vivência dos cuidadores formais no cuidado ao idoso com DA e lidando com as limitações do idoso. Na maioria dos depoimentos obtidos foi observado que a vivência do cuidador formal no cuidado a idosos portadores de demência vem carregada de situações estressantes e desgastantes, pois estes referiram sentir-se sem saber o que fazer perante os conflitos familiares, ora relacionados à parte financeira, comprar ou não a medicação, já que a doença não tem cura, ora relacionados às limitações do doente. Considera-se que a doença de Alzheimer é uma doença familiar que traz perdas avassaladoras, portanto para que estes profissionais prestem assistência adequada a essas famílias faz-se necessário que sejam capacitados para tal.

**DESEMPENHO DAS ATIVIDADES DE VIDA DIÁRIA E CUIDADOS COM O CORPO DURANTE A PRIMEIRA CONSULTA DE ENFERMAGEM A IDOSOS COM DEMÊNCIA NO DISTRITO FEDERAL** *Andrea Mathes Faustino; Luiza Rosa Bezerra Callado; Dilza Holland Martins Silva; Aline Cristina Martins Gratão; Carla Targino Bruno dos Santos; Keila Cristianne Trindade da Cruz*

**RESUMO. Introdução:** O atendimento ao idoso com Alzheimer deve ser realizado no contexto da equipe multiprofissional no qual o enfermeiro integra os múltiplos olhares. Por meio da entrevista no qual identifica o cuidador principal e pelo exame físico, é que permitem ao enfermeiro detectar diferentes problemas de saúde que resultarão em orientações direcionadas as necessidades do idoso no contexto do cuidado familiar. **Objetivos:** identificar por meio da análise do desempenho das Atividades Básicas de Vida Diária durante a primeira consulta de enfermagem ao idoso com demência as necessidades de cuidados com o corpo. **Métodos:** Trata-se de um estudo descritivo com caráter observacional por meio de levantamento de dados na primeira consulta de enfermagem realizada durante o acolhimento no Centro de Medicina do Idoso do Hospital Universitário de Brasília, Distrito Federal. **Resultados:** Amostra de 47 idosos, idade média 75,6 anos, 74,6% mulheres, sendo que têm como cuidador principal 46% os filhos e 31% o cônjuge. O diagnóstico principal dos idosos é a demência do tipo Alzheimer em fase leve ou moderada. Quanto a condição de dependência para realização das atividades básicas de vida diária as mais comprometidas são as relacionadas a higiene corporal e pessoal que em ambas apareceu como dependentes em 46% dos idosos, seguidas das atividades de supervisão para manter continência (44,7%) e de assistência para vestir (42%). A necessidade de auxílio para as transferências foi observada em apenas 29% dos idosos. Ao exame físico todos os idosos apresentaram ressecamento e desidratação da pele. Assim o direcionamento nas orientações de enfermagem foi feito no sentido de manter a integridade da pele, no cuidado durante o banho com o uso de água morna e sabonetes suaves, hidratação da pele diariamente, manutenção da umidade adequada da pele após trocas de fraldas e higiene íntima. **Conclusão:** É importante que os cuidadores tenham estas orientações tão logo seja possível, mesmo tendo ainda um idoso em condições de realizar transferência e com mobilidade preservada, pois pode-se prevenir complicações na pele quando este idoso vir a estar em fase mais avançada da demência e ficar com a mobilidade física prejudicada.

**QUEIXA SUBJETIVA DE COMPROMETIMENTO DA MEMÓRIA E ATIVIDADE NEUROENDÓCRINA DO ESTRESSE EM IDOSOS SAUDÁVEIS** *Marinete Esteves Franco; Juliana Nery de Souza-Talarico*

**RESUMO.** Dada a ampla variabilidade do desempenho cognitivo observada em idosos, diferentes autores têm demonstrado que o estresse, mais especificamente, os hormônios do estresse podem estar associados com prejuízo da memória. Neste sentido, o objetivo deste estudo foi analisar a relação entre queixa subjetiva de memória e padrão diurno de secreção de cortisol em idosos saudáveis. O presente estudo foi desenvolvido na cidade de São Paulo, foram incluídos 107 indivíduos com entre 50 e 82 anos (média=66,6±7,6) de ambos os sexos, com função cognitiva e funcional preservados. 41% (n=42), apresentaram queixa subjetiva de perda de memória detectada a partir do instrumento Memory Assessment Complaint Questionnarie - MAC-Q. Foram elegíveis para estudo indivíduos que assinaram o termo de consentimento informado pelo participante, com escolaridade ≥03 anos; com função cognitiva e funcional preservados, conforme a associação dos instrumentos de avaliação cognitiva MEEM (escore médio=27,6±1,5) e avaliação funcional IQCODE (escore médio=2,6±0,5). Foram excluídos indivíduos com diagnóstico de doença neurológica, em uso de glicocorticóides, de medicações psicoativas ou beta-bloqueadores; álcool ou drogas e fumantes ou história prévia há menos de 10 anos. Para avaliação do ritmo diurno de secreção de cortisol foram coletadas amostras de saliva em dois dias consecutivos, obtendo-se, então, a média de concentração de cortisol ao acordar, 30 minutos após acordar, à tarde (14h00 e 16h00) e ao dormir. Foi utilizado modelo de regressão multivariada tendo escores da escala de depressão geriátrica, escala de percepção de estresse e concentrações de cortisol como variáveis independentes e os escores do MAC-Q como variável dependente evidenciou efeito preditor do cortisol ao longo do dia (área sob a curva=AUC) na intensidade da queixa subjetiva de memória (β= -0,210; p=0,033). Aproximadamente 4,4% da variação na intensidade da queixa de memória ocorre em função do cortisol. Os resultados evidenciaram associação significativa entre padrão diurno de secreção de cortisol e queixa subjetiva de memória. Quanto maior a intensidade da queixa de memória menor a concentração cortisol ao longo do dia, sugerindo hipoatividade do eixo hipotálamo-pituitária-adrenal (HPA), regulador da secreção de cortisol, nos indivíduos com maior queixa de memória. Este estudo contribui para ampliar o conhecimento e auxiliar os profissionais da saúde no cuidado com o idoso.

### FISIOTERAPIA

**ANÁLISE DA QUALIDADE DE VIDA DE PACIENTES COM ACIDENTE VASCULAR ENCEFÁLICO APÓS TRATAMENTO FISIOTERAPÊUTICO** *Sheila Cristina Cecagno-Zanini; Felipe Rangel Medina; Cristina Cristovão Ribeiro da Silva; Camila Pereira Leguisamo; Luana Taís Hartmann Backes; Marcela Geisa Becegatto; Taís Romeu Ximendes Vaz da Silva*

**RESUMO. Introdução:** O acidente Vascular Encefálico (AVE) é uma das maiores causas de seqüelas permanentes que geram incapacidades funcionais. O aumento da população idosa no mundo aumenta o número de incapacidades advindas de doenças crônicas relacionadas com a idade, dentre elas, destaca-se as doenças cardiovasculares. O AVE é a terceira causa de morte nos países desenvolvidos, no Brasil corresponde a primeira causa de óbito. A qualidade de vida (QV) influencia na vida do ser humano. Muitos estudos tem se voltado para a medição de QV relacionado com a saúde, provavelmente pelos avanços terapêuticos, o que aumenta a expectativa de vida e oferece melhores resultados. **Objetivo:** Avaliar a QV de pacientes que sofreram AVE, antes e após tratamento fisioterapêutico, através da Escala de Qualidade de Vida Especifica para AVE (EQVE-AVE). Delineamento e métodos: Este estudo é do tipo quantitativo, observacional, longitudinal, prospectivo, coorte. O qual será utilizado o questionário - Escala de Qualidade de Vida Especifica (EQVE-AVE) para pacientes com AVE, antes e após o acompanhamento do tratamento fisioterapêutico. Foram selecionados 20 pacientes em Postos de Saúde com diagnóstico de AVE de no máximo três meses. Foi aplicado questionário EQVE-AVE no domicílio de cada paciente pelos pesquisadores, realizado em duas etapas, a primeira antes de iniciar o tratamento fisioterapêutico e a segunda após um ano do tratamento. Foram realizadas análises descritivas e de freqüência. **Resultados:** Média de idade 47 anos. Observou-se uma melhora na qualidade de vida dos pacientes após a intervenção da fisioterapia, com a redução dos escores de ajuda total de 42% para 2%, muita ajuda de 23% para 7%, alguma ajuda de 11% para 10%, também ocorreu um aumento em: um pouco de ajuda de 9% para 25%, e nenhuma ajuda necessária de 15% para 56%. **Conclusão:** O mecanismo de avaliação da qualidade de vida relacionada à saúde em indivíduos com seqüelas de AVE forneceu um perfil mais global das condições funcionais, psicossociais do paciente e de suas expectativas em relação á vida, direcionando assim a reabilitação. A grande maioria dos pacientes estudados se tornou independente ou semi-independente nas atividades cotidianas, indicando que o programa da fisioterapia tem um papel importante no quadro funcional destes indivíduos. Palavras-chave: Acidente vascular cerebral. Qualidade de vida. Fisioterapia.

**AVALIAÇÃO DA DEPRESSÃO E DO RISCO DE QUEDAS EM IDOSOS INSTITUCIONALIZADOS** *Roberta Bolzani de Miranda Dias; Marilene Rodrigues Portella; Jorge Augusto Barbosa de Sales Dias*

**RESUMO. Introdução:** A depressão é a doença psiquiátrica mais comum entre os idosos, frequentemente sem diagnóstico e sem tratamento. A depressão é um dos fatores intrínsecos de risco de quedas em idosos. A pessoa idosa com depressão tem 2,2 vezes mais possibilidade de cair se comparado a idosos que não têm depressão (RUBSTEIN E JOSEPHSON, 2002). **Objetivo:** Estudar a associação entre depressão e o risco de quedas em idosos institucionalizados. **Método:** Estudo transversal descritivo. A população foi composta por idosos institucionalizados de sete instituições para idosos do município. Os critérios de inclusão foram: ter 60 anos ou mais; deambular, mesmo que com dispositivo de auxílio à marcha e/ou ajuda de terceiros. Os critérios de exclusão foram: apresentar déficit cognitivo que impossibilite a compreensão/ imitação de ordens verbais e/ou atividades motoras simples; ser acamado (estar restrito ao leito) ou cadeirante; apresentar déficit visual e/ou auditivo severamente limitante e não compensado por uso de óculos ou aparelho de amplificação sonora. Foram usados como instrumentos de coleta de dados: Questionário com dados sócio-demográficos e clínicos; Mini Exame do Estado Mental (MEEM); Escala de Depressão Geriátrica (GDS) e o Timed Up and Go Test (TUGT). Os dados foram analisados através da estatística descritiva. Para comparar as frequências das variáveis qualitativas, empregou-se a estatística da razão de verossimilhança. A medida de correlação de Sperman foi calculada para medir o grau de associação da variável Time Up and Go Test com a depressão. O nível de significância considerado foi de 5%. **Resultados:** Participaram 40 idosos (27 mulheres e 13 homens), com média de idade de 78,8 anos, destes (50%) tinham depressão leve, 14 (35%) tinham depressão grave e 6 (15%) não apresentavam depressão. A partir do TUG avaliou-se o risco de quedas dos participantes, sendo que (45%) tinham um baixo risco, (22,5%) apresentavam um risco moderado e (32,5%) possuíam um alto risco. Não houve associação entre depressão e o risco de quedas, diferentemente de outros estudos (LOJUDICE, 2005; ANDRESEN et al., 2006; GONÇALVES et al., 2008). **Conclusão:** Apesar de não termos encontrado associação entre a depressão nos idosos institucionalizados e o risco de quedas, é de fundamental importância avaliar os fatores de risco para quedas, pois as estratégias de intervenção só terão eficácia quando os fatores forem identificados, minimizados ou até mesmo eliminados.

**EFEITOS DO TREINO DE TAREFAS FUNCIONAIS DE MEMBRO SUPERIOR REALIZADOS EM CONDIÇÕES DE TAREFAS SIMPLES E DUPLA EM PACIENTES COM DOENÇA DE PARKINSON** *José Eduardo Pompeu; Sandra Maria Alvarenga Anti Pompeu; Vanessa Silva Pereira; Caroline Kaori Tomo*

**ABSTRACT.** The clinical signs of Parkinson's disease are well known, but the cognitive changes, including the management of attention during motor learning processes, need to be investigated. Several studies have shown that patients with Parkinson's disease have worse performance in dual task condition, in which-they have to perform two tasks simultaneously. However, there are no studies about the best strategy to improve this deficiency. The aim of this study was to compare the effects of the training of functions of the upper limb of patients with Parkinson's disease carried out under single and dual task conditions. **Method:** This study was a case series of four patients with Parkinson's disease, with a mean age of 69.7 (4.8) years, in stages 2 and 3 of the Hoehn and Yahr scale. Patients were divided into two groups: group training in simple task (GTS) who performed four functionaltasks of the upper limb in simple task condition, and group training on dual task (GTD) that trained the same functions, but simultaneously with the performance of verbal fluency tasks. The trained tasks were: (1) comb hair, (2) answer the phone, (3) put on a coat and (4) take a cup to the mouth. The performance of the patients was assessed before and after training in two conditions:

(1) performance in single task condition and (2) performance in dual-task condition. In addition, patients were evaluated before and after the training with the Box and Block Test (B & B). **Results:** The patients‘ performance were assessed before and after training in two conditions: single task and dual task. The GTS showed improvement of 20% in the performance in simple task, while the GTD improved 156%. The GTS improved 61% in dual task and GTD improved 81%. Regarding the B & B, the GTS improved 114% after training and GTD improved only 6%. **Conclusion:** Both training, in simple and dual task condition, promoted improvement in the performance of manual tasks in patients with Parkinson's disease. In general, training on dual task condition promoted higher improvement than the training in simple task. However, the GTS promoted superior improvement in the B&B test.

**EFFECT OF A PHYSICAL THERAPY PROGRAM BASED ON THE GUIDELINES FOR THE REHABILITATION OF PARKINSON‘S DISEASE** *Bárbara Cristina Pedro; Jaqueline Lima da Silva; Juliana Thomé Gasparin; Sandra Maria Anti Pompeu; José Eduardo Pompeu*

**ABSTRACT.** Parkinson's disease (PD) is a progressive disease associated with motor and cognitive manifestations. Physiotherapy has as main objective to improve the basic activities like walking or performing transfers in order to improve the functionality, thus exerting an important role in the patient's rehabilitation and reintegration into their social living. Recently, the number of publications about the best practice of physiotherapy in PD has increased. However, most studies used isolated interventions such as muscle strengthening, balance training, training with external cues and cognitive strategy. Thus, this study aimed to develop and implement a protocol for physical therapy based on guidelines for physical therapy on Parkinson's disease and to assess its effects on balance, quality of life and cognition. **Method:** This is a case report study conducted at the Clinic of São Camilo University Center (PROMOVE). The study included nine patients with PD, six men and three women, with a mean age of 65.9 (8.5) in stages II and III of the Hoehn and Yahr. Patients were assessed before and after the intervention by the following instruments: Mini-BESTest, PDQ-39 and MoCA. Patients performed 14 sessions of physical therapy based on seven protocols that contemplate exercises performed in simple and in dual task conditions: (1) balance training on stable and unstable surfaces; (2) gait training with changes in direction and speed; (3) resistance exercises; (4) mobility exercises; (5) functional training with the use of external cues; (6) mental training; (7) observation of action and verbal instruction and (8) training of manual functions. During the data collection two patients drop out of the program due personal reasons. After the application of tests of normality and homoscedasticity, the results were analyzed by paired t tests, one for each variable assessed before and after the intervention, considering as statistical significance level alpha of 0.05. This study was approved by the Research Ethics Committee of the University Center, No. 229/08. **Results:** There was improvement of Mini-BEST and PDQ-39 after the intervention (t test; P<0.05). There was no significant improvement in the MoCA. **Conclusion:** The present study demonstrated that the physical therapy based on the guidelines for PARKINSON'S disease was effective for improving balance, gait, and consequently the quality of life of patients with PD, but did not have effect over the cognition.

**GAIT SPEED OF ELDERLY COMMUNITY: ASSOCIATION WITH COGNITIVE FUNCTION** *Daniel Henrique Ribeiro Costa; Lygia Paccini Lustosa; Rosangela Correa Dias; João Marcos Domingues Dias; Adriana Netto Parentoni; Juliana Magalhães Machado Barbosa; Rita de Cássia Guedes; Leani Souza Máximo Pereira*

**ABSTRACT.** The gait has been considered an important functional parameter, used to evaluate and estimate various health outcomes in the elders. Cognitive changes may compromise the performance on the gait, as well as the association of tasks, during the pacing, may compromise the shift, putting the elder at risk for falls. One of the instruments that has been used to evaluate cognitive function is the Addenbrooke's Cognitive Examination-Revised (ACE-R), consisted of five domains (Attention and Orientation, Memory, Fluency, Language and Visio-Spatial Abilities). After translation for the Brazilian population, the cutoff<78 points on the total score, showed a sensitivity of 100% and specificity of 82.3% for the diagnosis of mild Alzheimer's disease. **Objective:** To verify the association between cognitive function and the usual gait speed and gait with dual task in elderly community. **Methodology:** Participated 24 women (>60 years) living in the community who had a score on the Mini-exam of the mental state above the established cutoff point for scholarity. It was excluded those with neurological disorders, dependent gait and any orthopedic or rheumatic acute condition that could prevent the accomplishment of the gait. All the elders responded to the ACE-R for assessment of cognitive function. The gait speed was evaluated by the 10 meters test. The participants were instructed to walk on their usual speed (VMH) and subsequently to perform the same route doing subtraction calculations, named the gait speed with dual task (VMTD). The correlations were analyzed using the Spearman test. Significance level of 5%. **Results:** The average age was 72.0 (±5.6) years, body mass index of 29.3 (±5.3) kg/ m2, total score on the ACE-R 69.3 (±17.7). The average usual gait speed was 1.1 (±0.2) m/s while the average gait with dual task was

1.0 (±0.3) m/s. Cognitive function showed moderate correlation, affirmative, significant with the gait speed in dual task (r=0.52, p=0.01). Other associations were not significant (p>0.05).] **Conclusion:** The results showed an average of the total score of the ACE-R below the described in the literature, even in elders with scores compatible with scholarity on the Mini-exam of the mental state. Worse cognitive function demonstrated to be associated with worse performance on the gait speed during the execution of another activity. These data suggest a greater difficulty in the motor task performance, due to cognitive impairment.

**IMPACTO DAS ALTERAÇÕES POSTURAIS E INCIDÊNCIA DE QUEDAS NA QUALIDADE DE VIDA DE PACIENTES PORTADORES DE DOENÇA DE PARKINSON** *Renata Firpo R. Medeiros; Mercia Gomes Rodrigues; Iracelia Munhoz Moreira; Gisele Monaco Dias; Ana Lúcia Alves; Audrey Andrade Bertolini*

**RESUMO.** A doença de Parkinson (DP) é uma doença crônica e degenerativa do sistema nervoso central que acomete os núcleos da base. As principais manifestações da DP são: tremor de repouso, rigidez, bradicinesia, alterações posturais e marcha festinada. Tais sintomas interferem diretamente na funcionalidade, independência e qualidade de vida desses indivíduos. O objetivo desse estudo foi analisar a correlação entre as alterações posturais nos pacientes com DP, com a incidência de quedas, e correlacionar as quedas com a qualidade de vida. Foram sujeitos da pesquisa 10 indivíduos de ambos os sexos com idade média de 70,2 anos, com tempo de doença com média de 6,6 anos. Para realização da mesma foram utilizados: Avaliação geral do paciente, Questionário PDQ-39, Escala de Hoehn e Yahr, Escala de Schwab & England, Escala de equilíbrio de Berg, Software de análise postural - Fisio-Logic. Os dados colhidos passaram por uma análise estatística utilizando o coeficiente de correlação de Pearson (r). Com os resultados pôde-se observar que houve uma forte correlação encontrada entre o número de quedas sofridas e a alterações posturais dos indivíduos da amostra (r= -0,74). Também se constatou que os indivíduos que apresentaram episódios de quedas referiram uma pior percepção na sua QV, havendo forte correlação entre eles (r=0,85). Com esta pesquisa pode-se concluir que as alterações posturais têm intima relação com os eventos de quedas nesta população e também que os indivíduos da amostra que apresentaram um alto risco de quedas apresentaram um impacto negativo na qualidade de vida.

**MUSCULAR AND FUNCTIONAL PERFORMANCE IN ELDERS WITH COGNITIVE ALTERATIONS** *Daniel Henrique Ribeiro Costa; Lygia Paccini Lustosa; Rosangela Correa Dias; João Marcos Domingues Dias; Adriana Netto Parentoni; Juliana Magalhães Machado Barbosa; Rita de Cássia Guedes; Leani Souza Máximo Pereira*

**ABSTRACT.** Dementia stands out as one of the major causes of morbi-mortality in the elders, beyond the great impact on the functionality and independence. The Addenbrooke's Cognitive Examination-Revised (ACE-R) proposed to assess five domains (Attention and Orientation, Memory, Fluency, Language and Visio-Spatial Abilities). After adjustment for the Brazilian population, the cutoff score<78 points, on the total score, showed a sensitivity of 100% and specificity of 82.3% for the diagnosis of mild Alzheimer's disease. Gait speed and handgrip strength have been considered important functional parameters and has shown an association with various health outcomes in the elders. **Objective:** To compare the functional and muscular performance among elders classified above and below the cutoff point 78 in the ACE

R. **Methodology:** Participated 24 women (>60 yrs), living in the community and who had a score on the Mini-exam of the Mental State above the established cutoff point for scholarity. It was excluded those with neurological disorders, dependent gait and any orthopedic or rheumatic acute condition that could prevent the accomplishment of the tests. All the elders responded to the ACER and were divided into two groups according to the result of the total score (cutoff 78). The functional performance was evaluated by the 10m walking test. The muscular performance was estimated through the handgrip strength test (Jamar^®^ dynamometer). It was used the Mann-Whitney test for comparison between groups (α=5%). **Results:** Fourteen elders were allocated in group I (<78 in the ACE-R) and 10 in group II (>78 in the ACE-R). The group I had an average age of 73.5 (±4.3) yrs, body mass index (BMI)

29.6 (4.4) kg/m2 and total ACE-R of 57.4 (±13, 3). In group II, the average age was 69.9 (±6.8) years, BMI 28.8 (6.6) kg/m2 and total ACE-R of 85.5 (±5.9). The handgrip strength in group I was 21.3 (±6.8) kg / f and in group II 20.1 (±3.0) kg / f (p=0.611). The group I developed gait speed of 1.05 (±0.2) m/s and group II of 1.27 (±0.2) m/s. There was difference between groups (p=0.008), demonstrating that the group ranked below the cutoff point in the ACE-R was slower. **Conclusion:** It was observed that elders with possible cognitive alterations detected by the ACE-R had worse gait performance, but no differences in muscular strength. These results confirm the assumption that the gait speed is an important parameter in the clinical evaluation of the elders.

**TREINO DE EQUILIBRIO EM IDOSAS DEMENCIADAS INSTITUCIONALIZADAS COM A INTERVENÇÃO DO WII** *Renata Firpo R. Medeiros; Mercia Gomes Rodrigues; Iracélia Munhoz Moreira; Ana Lúcia Alves; Gisele Monaco Dias; Audrey Andrade Bertoloni*

**RESUMO. Introdução:** A população idosa está crescendo consideravelmente a cada ano. O envelhecimento é um processo inevitável que aparece gradualmente com a passagem do tempo, surgindo alterações biológicas e fisiológicas. O equilíbrio demanda a permanência do centro de gravidade sobre a base de suporte em condições estáticas e dinâmicas, este é necessário para a manutenção da postura, que depende dos sistemas visual, vestibular e proprioceptivo. **Objetivo:** avaliar a capacidade de equilíbrio em idosos com a intervenção da realidade virtual. **Métodos:** A amostra é constituída por 6 idosas, foi utilizado o vídeo Wii Fit da marca Nintendo, plataforma balance board e o Jogo Wii Fit Plus, para a avaliação pré e pós intervenção utilizamos a escala de equilíbrio de Berg, Time Up and Go, mini exame do estado mental (MEEM), com duração de 30 minutos cada, sendo 10 sessões, duas vezes por semana. **Resultados:** com uma análise comparativa pré e pós intervenção, as participantes deste estudo apresentaram uma melhora significativa na escala de equilíbrio de Berg e no Time Up and Go. Os valores de frequência cardíaca, frequência respiratória e pressão arterial sistólica e diastólica não apresentaram significância. **Conclusão:** Um treinamento especifico de equilíbrio com a intervenção da realidade virtual, em idosas institucionalizadas com déficit de equilíbrio de leve á moderado, mostrou ser um recurso com alto grau de motivação e efetivo na melhora do equilíbrio estático e dinâmico.

### FONOAUDIOLOGIA

**ANALYSIS OF SEMANTIC-LEXICAL ERRORS IN THE DISCOURSE OF PATIENTS WITH ALZHEIMER'S DISEASE, COMPARED TO APHASICS AND HEALTHY INDIVIDUALS** *Paola Darbello da Silva; Juliana Onofre de Lira; Karin Zazo Ortiz*

**ABSTRACT.** Aphasia is defined as a language disorder stemming from a focal lesion in the central nervous system. Alzheimer's disease (AD) is the most frequent type of dementia and can affect all aspects of language. The semantic-lexical impairments found in AD patients can also be seen in the various aphasias after stroke. One of the proposed methods for assessing utterances involves eliciting spontaneous speech or discourse. **Objective:** To compare the discourse produced by AD patients, aphasics and healthy subjects based on a picture description task. **Method:** A cross-sectional study was conducted analyzing speech produced during the Cookie Theft task in 31 control subjects, 26 aphasics and 33 AD patients. Parameters assessed included total words uttered, information units as well as semantic-lexical and phonologic errors. Analysis of variance (ANOVA) was applied to determine differences among means for the variables described. **Results:** No statistically significant difference in schooling was detected among the groups. Mean age in the control group was higher than in the aphasic group but lower than in the AD group. For gender, there was a higher proportion of women in the control group compared to the other groups. No difference was observed between the aphasic and AD groups in number of words and information units produced, although both these frequencies were lower than in the control group. Regarding errors, only the phonemic type showed a statistical difference, with a higher frequency observed in the aphasic group. For total number of errors, results were based on the proportion of words, and the error rate among AD patients was significantly higher than in controls but similar to aphasics. **Conclusion:** The performance of aphasic individuals was similar to that of AD patients in the quantitative aspect, while both these groups performed worse than the control group for content and number of errors.

### GENÉTICA

**ACE POLYMORPHISM AND USE OF ACE INHIBITORS: EFFECTS ON MEMORY PERFORMANCE** *Fabiana Michelsen de Andrade; Jaqueline Bohrer Schuch; Pamela Camini Constantin; Vanessa Kappel da Silva; Tatiane Jacobsen da Rocha; Marilu Fiegenbaum; Alcyr de Oliveira Jr,; Camila Korb; Claudia Justin Blehm; Daiani Pires Bamberg; Luciana Alves Tisser*

**ABSTRACT.** Memory is an important cognition function, being fundamental to the development and independence of individuals. The renin angiotensin system has been studied regarding its role on brain, and could have interference in memory modulation. The key element of this system is the angiotensin converting enzyme (ACE), and an insertion/deletion polymorphism (I/D) in the gene that encodes this enzyme might alter its activity, therefore influencing memory. Our aim was to investigate the influence of ACE polymorphism and ACE inhibitors use, besides their interaction on memory performance of healthy subjects over 50 years. Initially 367 volunteers were tested, but 162 were excluded for using any kind of psychotropic medication, or owned estimated IQ below 70, anxiety, depression or stress, assessed by psychological tests. Final sample consisted of 205 subjects (64.0±8.08 years, 24% of men) assessed for five types of episodic memory, using Wechsler Memory Scale-Revised (WMS-R), who answered a questionnaire about drug use, and were assessed for the ACE insertion/deletion polymorphism trough PCR and elefctrophoresis. ANCOVA was used to accesses influences of ACE inhibitors and polymorphisms, both as single variables as interactions. All statistical analyses were done through SPSS v. 20.0. The use of ACE inhibitors beneficially influenced learning ability scores (p=0.02). Besides, I allele carriers of ACE polymorphism showed higher verbal memory scores compared with homozygous DD. Also we observed an interaction influencing learning ability between the ACE polymorphism and the use of inhibitors, the beneficial influence of the I allele was present only in individuals who make use of ACE inhibitors. We conclude that the ACE gene has influence on memory performance, and that this influence is modulated by ACE inhibitors use. This is the first time this pharmacogenetic interaction is detected, but if these data could be replicated by other authors, it should be used in the future in memory deficit prevention.

**ANALYSIS OF INTERACTION BETWEEN GENE GSTP1 AND EXPOSURE TO PESTICIDES IN SUSCEPTIBILITY FOR PARKINSON‘S DISEASE** *Bruna Bellini; Juliana Foresti Caprara; Jéssica Brasil Figueiredo Meyer; Malisia Balestrin Lazzari; Artur Schuch; Carlor Rieder; Mara Helena Hutz; Fabiana Michelsen de Andrade*

**ABSTRACT.** Parkinson's disease (PD) is the second most common neurodegenerative disorder in the world, with multifactorial etiology involving complex interactions between genetic and environmental factors. Several risk factors have been identified to PD, including occupational exposure to pesticides. In this contest, the accumulation of toxic substances associated with polymorphisms in genes of detoxification enzymes family could lead to a higher risk to develop PD. Among then, the glutathione S-transferase P1 gene (GSTP1) is a candidate gene, once the Ile105Val polymorphism is related to lower enzyme activity. The aim of this study is to analyze if there is interactions between the GSTP1 gene polymorphism and environmental exposure on the DP. To date were collected DNA samples from 113 patients previously diagnosed with PD and compared with 184 controls. The analysis of the GSTP1 gene was made by PCR / RFLP (83 patients and 94 controls genotyped), and exposure to pesticides was evaluated by using a retrospective questionnaire, in which participants answered questions related to previous occupation, exposure to pesticides at work, what type of pesticide, among others. Answers about environmental exposure and genotype frequencies were compared by chi-square test using SPSS software, version 20.0. When the GSTP1 genotype frequencies were compared between patients and controls, no significant difference was detected. We observed a trend that more patients than controls were farmers (21.2% patients vs. 13% of controls, p=0.065). The number of patients who used pesticides at work was higher when compared to the control group (15.9% patients vs. 6.5% controls, p=0.009), especially regarding the use of herbicides and insecticides. These data indicate that there is a significant relation between exposure to pesticides and the risk of developing PD with a trend of increased risk for farmers compared to other occupations. No significant interaction between carriers of the G allele (in principle the risk allele) and previous occupation as farmer or pesticide use at work was found, which may be caused by the small sample size analyzed so far. The study is ongoing in order to increase the statistical power and to conduct further analysis to determine relevant data on possible risk factors for PD.

GENE X ENVIRONMENT INTERACTIONS: EVIDENCE OF ADDITIVE EFFECT BETWEEN APOE*2 ALLELE AND COFFEE CONSUMPTION IN PROTECTION FOR PARKINSON‘S DISEASE *Juliana Foresti Caprara; Bruna Bellini; Jéssica B.F. Meyer; Malisia B. Lazzari; Artur Schuch; Carlos Rieder; Mara H. Hutz; Fabiana M. de Andrade*

**ABSTRACT.** Parkinson's disease (PD) is one of the most common neurodegenerative disease in the world, caused by complex interactions between genetic and environmental factors. Several risk factors were found for PD, and between them can be E*4 allele of the apolipoprotein E (APOE) gene, wich was associated with increased risk by a few authors. On the other hand, some data show that protective factors may exist, such as coffee consumption, and the presence of E*2 allele of the same gene. Substances in coffee originated from the metabolism of caffeine as paraxanthine, theophylline and theobromine appear to act as antagonists of adenosine A receptor, bringing a neuroprotective effect of PD. However, it is not known how genetic risk factors interact with the possible protection of coffee consumption. The objective of this study is to analyze if there is interaction between the APOE gene and coffee consumption on the development of Parkinson's disease. So far, were collected DNA samples from 113 patients previously diagnosed with PD and compared with 139 controls. The analysis of the APOE gene were performed by PCR / RFLP (107 patients and all controls genotyped), and previous coffee consumption was evaluated by using a retrospective questionnaire, in which participants answered whether they had consumed coffee in the past, and chose any option related to the number of cups of coffee consumed per day in most part of life. When APOE genotype frequencies were compared between patients and controls, no significant difference was detected. Never have consumed coffee was more frequent in patients than in controls (26.7% vs 4.3%, P=0.00004). No significant interaction between the presence of the E*4 allele (at first, the risk allele) and no coffee consumption could be detected (P=0.364). However, when the protective influence of E*2 allele was tested together with the presence of coffee consumption, a significant interaction was possible: don't have this allele together with never have consumed coffe were more often in patients (27, 5% vs. 4.1%, P=0.000011). These data indicate that the coffee consumption could act as protectors for PD, especially in the presence of E*2 allele, while the presence of E*4 allele appears to act not as a risk factor, either singly or together with the lack of coffee consumption. However, the increasing the sample size may allow the determination of both new data on risk factors and protective factors for Parkinson's disease.

**INTERACTION OF NULL GENOTYPES IN TWO GST GENES AND PESTICIDE EXPOSURE ON SUSCEPTIBILITY TO PARKINSON‘S DISEASE** *Malisia Balestrin Lazzari; Jéssica B. F. Meyer; Juliana F. Caprara; Bruna Bellini; Artur Schuch; Carlos Roberto de Mello Rieder; Mara Helena Hutz; Fabiana Michelsen de Andrade*

**ABSTRACT.** Brazil is taking the place of largest consumer of pesticides in the world and Rio Grande do Sul is the 4th largest consumer in the country. The growing increase of pesticides can bring negative impacts on health, including the development of chronic diseases, such as Parkinson's disease (PD). PD is the second most common neurodegenerative disorder in the world, resulting from the degeneration of dopaminergic neurons, which causes the motor dysfunctions features. PD has a multifactorial etiology resulting from the interaction of environmental factors and genetic susceptibility, with possible influence of glutathione S-transferases (GSTs), enzymes involved in xenobiotics metabolism. Our aim was to evaluate the interaction of GSTT1 and GSTM1 and pesticide exposure on the risk of Parkinson's disease. The sample includes 146 PD patients and 202 controls, subjects with more than 50 years, with no neurological disease diagnosed. In total, comprises 28.7% of men with an average age of 62±9.48 years. Both groups answered questionnaires regarding retrospective environmental exposure and had the null genotype of selected genes analyzed by PCR and electrophoresis. Among the patients, 21.2% reported being farmers, compared with 13.9% of controls (p=0.099). Also, 15.9% of patients reported have used pesticides at work, compared to 7.4% controls (p=0.019). The frequency of null genotypes for GSTM1 and GSTT1 genes did not differ between groups. Due to the sample size, analysis of gene x environment interaction was performed only with GSTM1 and previous use of pesticides. A larger number of patients possessed the null genotype together with reporting of work with pesticides in agriculture, compared to controls (7.9% vs 3.0%, p=0.196), although this difference has not reached statistical significance. This study provides evidence that environmental factors such as occupational contact with pesticides are related to increased risk of Parkinson's disease, although it was not possible to detect any influence of gene x environment interaction with this sample size. Thus, sample collection is still in progress to enable the increasing in the statistical power. Keywords: Parkinson's disease, pesticide use, gene x environment interaction

**INTERACTIONS ANALYSIS BETWEEN GSTM1 GENE AND SMOKING ON SUSCEPTIBILITY FOR PARKINSON'S DISEASE** *Jéssica Brasil Figueredo Meyer; Bruna Bellini; Malisia B Lazzari; Juliana Foresti Caprara; Artur Schuch; Carlos Rieder; Mara H. Hutz; Fabiana M. de Andrade*

**ABSTRACT.** Parkinson's disease (PD) has a multifactorial etiology, which includes the participation of both environmental and genetic factors, not yet fully understood. Several studies report that substances present in cigarettes can bring beneficial effects for PD, acting in the neuroprotection. Furthermore, some of the candidate genes include xenobiotic metabolizing genes such as GSTM1, which deficiency could be related to increased or decreased PD risk, depending on what kind of substances the individual is in contact (if neuroprotective or damaging substances). Individuals with null GSTM1 genotype are deficient in this enzyme and have a reduced capacity of conjugation of xenobiotics, such as substances present in cigarettes. Therefore, if smoking is protective, it should be more effective in subjects with null genotype, but this hypothesis were never tested before. This study aims to investigate interactions between exposure to smoking and the presence of the null gene GSTM1 genotype on susceptibility to Parkinson's disease. We evaluated 113 patients with Parkinson's disease and 164 control subjects. The presence of a null genotype for GSTM1 was evaluated by PCR. Both groups answered a retrospective questionnaire with questions related to smoking. When investigated in isolation, smoking was not significantly associated with PD, as it was not possible to detect the influence of the GSTM1 gene. However, using a multivariated analysis with the inclusion of prior exposure to pesticides as a cofactor, it was possible to detect a protective role of smoking in relation to the presence of PD (OR=0.5, p=0.049). However, it was not possible to detect any interaction between smoking and GSTM1 gene, although the presence of GSTM1 has had a borderline protective effect (OR=57, p=0.076). Therefore, the data from this study confirm the smoking habit and the presence of the GSTM1 gene as protective factors for PD. The sample size is being increased, what can turn possible to find new and/or stronger influences.

**MUTAÇÕES NO GENE DA PROGRANULINA EM CASUÍSTICA BRASILEIRA DE DEGENERAÇÃO LOBAR FRONTOTEMPORAL** *Leonel Tadao Takada; Valéria Santoro Bahia; Thais V M M Costa; Jessica Ruivo Maximino; Roberta Diehl Rodriguez; Karolina Gouveia Cesar; Jerusa Smid; Fabio H G Porto; Sonia M D Brucki; Gerson Chadi; Ricardo Nitrini*

**RESUMO. Introdução:** Mutações no gene de progranulina (GRN) causam apresentações clínicas do espectro das degenerações lo-bares frontotemporais (DLFTs). Em casuísticas internacionais, mutações de GRN foram encontradas em 4-24% dos casos de DLFTs familiais. **Objetivo:** determinar a frequência de mutações de GRN em casuística brasileira de DLFT. **Métodos:** Incluimos pacientes com diagnóstico clinico de doenças do espectro das DLFTS: variante comportamental da demência frontotemporal (vcDFT), demência semântica (DS) e afasia progressiva não fluente (APNF). DNA foi extraído de sangue periférico e a pesquisa de mutações foi feita por sequenciamento direto dos exons 1-12 do gene. **Resultados:** O estudo está em andamento, e até o momento foram incluidos 58 probandos (41 com vcDFT, 9 com APNF e 8 com DS). A mediana da idade de início de cada grupo foi de: 52 anos para vcDFT, 64 anos para APNF e 61 anos para DS. Quatro probandos (3 com vcDFT e 1 com DS) também apresentavam sinais de doença do neurônio motor. Foram sequenciados os genes de 7 probandos, e foi encontrada uma mutação Q300X em uma paciente com diagnóstico de APNF. **Conclusão:** mutações no gene da progranulina também são encontradas em casuística brasileira de DLFT. A frequência de mutacões ainda necessita ser investigada com estudo genético da casuística completa.

### GERIATRIA/GERONTOLOGIA

**A UNIVERSIDADE COMO ESPAÇO DE TROCA DE SABERES ENTRE PROFISSIONAIS E CUIDADORES DE PACIENTES COM ALZHEIMER** *Mário Roberto Agostinho da Silva; Márcia Carréra Campos Leal; Ana Paula de Oliveira Marques; Jaime Roberto Tavares de Lima*

**RESUMO. Introdução:** A doença de Alzheimer requer atenção especializada, envolvendo ações multidisciplinares e a família. Além das alterações funcionais a mudança e instabilidade no comportamento do paciente requer maior atenção do cuidador. As relações com o paciente no seio familiar desencadeiam sentimentos os mais diversos, o cuidar necessita de um olhar diferenciado, evitando o adoecimento do cuidador. **Objetivo:** Apresentar experiência de projeto de extensão universitária com cuidadores de pacientes com Alzheimer. **Métodos:** Relato de projeto de extensão com Equipe multiprofissional desenvolvido no Núcleo de Atenção ao Idoso da UFPE, Serviço especializado Geronto-Geriátrico, iniciado em 2005, cadastrado em fluxo contínuo, periodicidade semestral, envolvendo profissionais de Psicologia, Enfermagem, Nutrição, Terapia Floral, Medicina, Fisioterapia, Instituições parceiras (ONGs e Governamentais) docentes e discentes universitários. Realizado em encontros quinzenais de duas horas. Os cuidadores foram inscritos a partir dos ambulatórios do Serviço onde são acompanhados os pacientes com Alzheimer e de outros serviços. Após a composição do grupo a Equipe de trabalho, através de dinâmica dialogada, levanta a demanda dos cuidadores, possibilitando o desenvolvimento das oficinas atendendo as necessidades do grupo. Em cada encontro é realizada avaliação verbal entre os envolvidos para averiguar o alcance dos objetivos. O saber individual e coletivo é valorizado e o autocuidado é ressaltado como meio para a manutenção do estado de bem-estar. **Resultados:** Grupos com média de 15 participantes, com predominância familiar e feminina. Entre os temas solicitados os cuidadores apresentam maior dificuldade quanto ao diagnóstico, a incerteza é referida como propulsora de ansiedade. O lidar com as alterações do comportamento do paciente e a falta de assistência na divisão de tarefas com os demais familiares foram referidos como geradores de sobrecarga e estresse, desencadeando sintomas depressivos como sentimentos de culpa e exacerbação da agressividade. **Conclusão:** O trabalho em grupo possibilita ao cuidador a verbalização dialogado de seus sentimentos e a troca de saberes no cuidado com o paciente, onde a escuta, acolhimento e orientação do cuidador pela Equipe no manejo do comportamento do paciente e do autocuidado propiciam um espaço acolhedor possibilitando a ressignificação do modo de lidar com o paciente, a doença e consigo mesmo, melhorando sua qualidade de vida.

**A VISÃO DO IDOSO ONCOLÓGICO FRENTE À MORTE: UMA ANÁLISE GERONTOLOGICA** *Marjore Grace Luizon de Brito; Vivian Ramos Melhado*

**RESUMO.** O aumento no número de idosos na população e a sua maior expectativa de vida tem como consequência, uma maior incidência e prevalência de doenças crônicas, entre elas as neoplasias. O desenvolvimento de uma neoplasia representa uma ameaça a vida do individuo, e traz consigo uma ampla gama de sentimos, visto que os desdobramentos são, muitas vezes, não esperados e modificam e interrompem as atividades cotidianas. Com o intuito de compreender a vivencia de idosos dentro desta problemática, realizou-se uma pesquisa por meio da Revisão Sistemática Integrativa para identificar, analisar e sintetizar as evidências disponíveis na literatura e responder a seguinte pergunta: Qual é a visão que o idoso oncológico tem sobre a morte? A análise dos dados foi feita com o auxílio do programa START - UFSCar. Os resultados foram apresentados em quatro grandes eixos que emergem dos artigos selecionados: medo e aceitação da morte, religiosidade, ressignificação do câncer e esperança. Espera-se que diante desses dados seja possível proporcionar conhecimentos para uma assistência de melhor qualidade neste momento especial da vida do idoso e seus familiares.

**AÇÃO EXTENSIONISTA COM EQUIPE MULTIDISCIPLINAR NO CONTROLE DO EXCESSO DE PESO EM IDOSOS** *Mario Roberto Agostinho Silva; Maria da Conceiçao Chaves Lemos; Tanea Campos Fell Amado; Jaime Roberto Tavares de Lima*

**RESUMO.** Intrudução: Ansiedade e depressão são transtornos afetivos associados à obesidade, doença crônica, inter-relacionada direta ou indiretamente com algumas outras situações patológicas contribuintes da morbimortalidade como as doenças cardiovasculares, osteomusculares e neoplásicas, atingindo cerca de 1/3 da população adulta, com tendência crescente nas ultimas décadas, inclusive entre os idosos. **Objetivo:** Identificar os principais fatores referidos pelos idosos associados ao sobrepeso e obesidade. Orientar sobre hábitos alimentares saudáveis, estimular educação alimentar, incentivar a prática de atividade física e sensibilizar para redução de 5% do peso corporal, minimizando risco a saúde. **Método:** Relato de projeto de extensão desenvolvido em unidade geronto-geriátrica, de maio a dezembro de 2012, por Equipe multidisciplinar: nutricionistas, psicólogo, educador físico e discentes de nutrição. Foram inscritos 66 idosos de ambos os sexos, com idade igual e superior a 60 anos, com excesso de peso e obesos. Participaram de palestras semanais de forma dialogada com entrega de material didático e submetidos a atendimento/avaliação do estado nutricional: antropométrica e bioquímica. **Resultados:** Do total, apenas 22 idosos finalizaram o projeto e referiram como fatores associados ao sobrepeso e obesidade: hábitos alimentares incorretos, problemas endócrinos/fisiológicos, ausência de orientação nutricional adequada, sedentarismo, ansiedade, depressão, conflitos interpessoais, isolamento social, pouco recurso financeiro para adesão a dieta e a influencia de terceiros. Dos concluintes, 12 apresentaram perda de peso, destes 4 perderam 5% do peso corporal e 10 mantiveram o peso, mas revelaram mudança nos hábitos alimentares. A verificação da redução de 5% do peso e da mudança de hábitos alimentares foram obtidas através da comparação dos dados antropométricos da primeira e última avaliação nutricional e teste de fixação de conhecimento pós-palestras. Aprender a lidar com as emoções e as adversidades foi referido como fator para elevação da autoestima e conquista dos objetivos propostos. **Conclusão:** A mudança no padrão de comportamento alimentar depende de vários fatores, principalmente da adesão do paciente e orientação adequada. Melhorar o estilo de vida e o comportamento alimentar do idoso requer maior atenção, pois estes possuem hábitos alimentares já sedimentados ao longo da vida e apresentam maior dificuldade em lidar com os fatores biopsicossociais do envelhecer.

**ALTERAÇÃO NO PERÍMETRO CRANIANO DURANTE O ENVELHECIMENTO** *Juliana Oliveira Martins; Natália Pugliessa Isídio da Silva; Ricardo Caires Neves; Daniela de Souza Farias; Carlos Augusto Pasqualucci; Ricardo Nitrini; Wilson Jacob-Filho; Renata Eloah de Lucena Ferretti-Rebustini*

**RESUMO. Introdução:** O envelhecimento está associado às alterações morfométricas crânio-encefálicas. É necessário saber se o perímetro craniano diminui no envelhecimento em indivíduos livres de comprometimento cognitivo. **Objetivo:** Verificar se existe diminuição do perímetro craniano durante o envelhecimento, segundo gênero e faixa etária. **Metodologia:** No Serviço de Verificação de óbitos da Capital, 100 indivíduos sem comprometimento cognitivo foram submetidos à aferição do perímetro craniano, por meio de uma fita métrica inelástica, após rebaixamento de todo o couro cabeludo. A amostra foi estratificada para gênero e grupo etário (idosos e não idosos). O nível de comprometimento cognitivo foi verificado pelo Escore Clínico de Demências (CDR 0) por meio da entrevista clínica com os familiares do sujeito, após consentimento informado. Para as correlações, foi utilizado o teste de correlação de Spearman's. Foi considerado significante o p-valor <0,05, para um α de 5%. **Resultados:** Amostra composta por 50 casos de cada gênero e 61% de idosos. A idade média foi de 67,61±12,3, maior entre as mulheres (70,04±12,0). O perímetro craniano médio entre os não idosos foi de 51,3±1,9cm e nos idosos foi de 50,4±1,5cm. O perímetro craniano se mostrou me-nor com o aumento da idade (r= -0,240; p=0,016), e menor entre as mulheres (p<0,00), tanto em idosos quanto em não idosos. Quando ajustados para a altura, a redução permanece. **Conclusão:** Existe diminuição da circunferência craniana durante o envelhecimento em indivíduos idosos, sem comprometimento cognitivo, em ambos os gêneros.

**ANÁLISE COMPARATIVA DAS CARACTERÍSTICAS CLÍNICAS ENTRE PACIENTES COM COGNIÇÃO NORMAL E COM DEMÊNCIA CDR 1** *Gabriella Polastri Stiilpen; Leonardo Vinícius de Andrade; Clarissa de Angelis Vieira dos Santos; Sandra Cristina Maciel de Lacerda; Deborah Mendonça Lima; Marco Túlio Gualberto Cintra; Maria Aparecida Camargos Bicalho; Flávia Lanna de Moraes; Érica Olive*

**RESUMO. Introdução:** Os fatores de risco associados com demência são alvos de diversos estudos, entretanto são encontrados dados conflitantes. Os mecanismos pelos quais cada um dos fatores de risco contribui para o desenvolvimento da doença não são bem definidos. **Objetivo:** Avaliar se existe associação entre variáveis sócio demográficas e clínicas e a presença de demência CDR1 atendidos por um serviço de referência em geriatria. **Métodos:** Estudo transversal abrangendo uma amostra de 359 idosos atendidos pelo Programa Mais Vida em Belo Horizonte, sendo 284 com cognição normal e 75 portadores de demência CDR1. Realizamos análise comparativa dos fatores sócio demográficos e clínicos possivelmente associados a demência entre os dois grupos. Foram utilizados os testes Qui-quadrado e Fisher para variáveis categóricas e os testes t e Mann-Whitney para variáveis contínuas. **Resultados:** Ao compararmos as duas amostras observamos que o sexo masculino elevou o risco de demência em quase três vezes (OR 2,93; p< 0,001). Como esperado, as atividades instrumentais de vida diária associam-se com demência (OR 18,36; p<0,001). Acidente vascular encefálico (AVE) aumentou o risco em três vezes (OR=2,99; IC95% 1,27-7,02; p=0,005). Deficiência de vitamina B12 (valor menor que 350pg/ml) aumentou o risco de demência em 2,68 vezes (OR: 2,68; IC95% 1,44-5,03; p=0,001). hipoacusia aumentou o risco em 71% (OR: 1,71; IC95% 1,00-2,95; p=0,038). A dislipidemia (OR 0,47; IC95% 0,23-0,94; p=0,021) e osteoartrite (OR: 0,46; IC95% 0,20-1,01; p=0,035) foram considerados como fatores de proteção Não observamos diferença estatística na análise das seguintes variáveis: tabagismo, etilismo, depressão, hipertensão arterial, diabetes, infarto agudo do miocárdio, incontinência urinaria, hipoacuidade visual, instabilidade postural e insuficiência familiar. A análise multivariada mostrou que os pacientes com demência são mais velhos (p<0,001), apresentam menor clearence de creatinina (p=0,004), menor IMC (p<0,001) e menor circunferência de panturrilha (p=0,001). **Conclusão:** Observamos associação entre demência e idade avançada, valores reduzidos de IMC e circunferência de panturrilhas e com redução do clearance de creatinina na amostra estudada.

**APLICAÇÃO DO VULNERABLE ELDERS SURVEY (VES-13) EM AMBULATÓRIO DE GERIATRIA** *Cristiana Ceotto Deslandes; Marco Túlio Gualberto Cintra; Flávia Lanna de Moraes; Edgar Nunes de Moraes; Avelina Cristina Conceição*

**RESUMO. Introdução:** O padrão ouro para determinar o declínio funcional, marcador de estado de saúde em geriatria, é a avaliação geriátrica ampla (AGA). No entanto, a AGA é uma avaliação extensa e que exige Know-how, sendo interessante a aplicação de instrumentos para determinar quais idosos poderão ser favorecidos pela aplicação da AGA. Um instrumento interessante e validado em nosso meio é o Vulnerable Elders Survey (VES13). **Objetivo:** Estudar a efetividade do VES-13 para determinar os pacientes que necessitam de atendimento prolongado em serviço de geriatria comparando com os resultados obtidos pela AGA. **Metodologia:** Estudo transversal abrangendo amostra de 109 pacientes atendidos no período de março e abril de 2013 em ambulatório de geriatria de Belo Horizonte-MG. Foram utilizados os testes Qui-quadrado e Fisher para variáveis categóricas e os testes t e Mann-Whitney para variáveis contínuas, além da curva ROC e teste de regressão logística binário. A análise estatística foi realizada pelo pacote estatístico SPSS 19.0. **Resultados:** Os 109 pacientes apresentavam média de 75,3±7,7 anos, sendo 75,2% do sexo feminino, 18,3% dependentes para atividades de vida diárias (AVD's) básicas e 56% dependentes para AVD's instrumentais, 26,6% com demência, 45,9% com depressão, 47,7% com instabilidade postural e 39,4% com incontinência urinária. 26,6% apresentavam indicação de atendimento prolongado em serviço de geriatria. Após análise univariada, observou-se relação do VES-13 com os pacientes encaminhados ao serviço de geriatria de referência (p<0,001), com demência (p<0,001), com instabilidade postural (p<0,016), com acometimento de AVD's básicas (p<0,001) e instrumentais (p<0,001) e com incontinência urinária (p=0,010). O VES-13 não foi influenciado pelo fator gênero (p=0,817), mas é pela idade (p<0,001), pela circunferência de panturrilha (p=0,017) e pelo índice de massa corpórea (p=0,006). A análise multivariada indicou que são determinantes no resultado do VES-13 a dependência para as AVD's instrumentais (p<0,001), a idade (p<0,001) e a dependência para AVD's básicas (p=0,031). A curva ROC indicou área sob a curva de 0,725 em relação a AGA para detectar o paciente que necessita de acompanhamento geriátrico. **Conclusão:** O VES-13 é uma ferramenta com resultados determinados pela funcionalidade, mas influenciados pela idade. Representa um teste regular em relação a AGA na detecção de idosos frágeis com necessidade de assistência especializada de geriatria.

**ASSOCIAÇÃO ENTRE O ESCORE CAIDE E O MOCA TESTE NO AMBULATÓRIO DA MEMÓRIA UNISUL (AMEMO)** *André Junqueira Xavier; Céline Yasmine Schweri; Felipe Tibúrcio Alves Vieira Perez; Isadora Weltercpioresan; Mayara Fernanda Pacovska; Pâmela Nogueira Da Silva Vilela*

**RESUMO. Introdução:** O Cardiovascular risk factors, Aging and Dementia (CAIDE) avalia idade, escolaridade, genero, pressão arterial sistólica, IMC, colesterol total e atividade física, o escore máximo é de 15 pontos. Escore entre 0-5 pontos indica risco estimado em 1% de evoluir com demência em 20 anos, risco de 1,9% para escore de 6-7, 4,2% para 8-9, 7,4% para 10-11 e 16,4% para escore entre 12 e 15. O Montreal Cognitive Assessement (MoCA) é capaz de detectar Transtorno Cognitivo Leve (TCL) e demências. O MoCA avalia função executiva, capacidade visuo-espacial, atenção, concentração e memória de trabalho, linguagem e orientação, varia de 0 e 30, a pontuação abaixo de 26 indica casos suspeitos de perda cognitiva. **Objetivo:** Analisar a associação entre o CAIDE e o MoCA na avaliação dos pacientes do Ambulatório da Memória da Unisul (AMemo) e estabelecer o melhor ponto de corte do CAIDE em relação ao déficit cognitivo (Moca<26). **Métodos:** Estudo transversal, análise por teste não paramétrico para duas amostras relacionadas, (Teste de McNemar, de Cochran Q, Wilcoxon Mat-ched-Pair Signed-Rank, ou Friedman 2-Way ANOVA by Ranks) e análise ROC para obtenção do ponto de corte. Participaram pacientes do AMU (censo) com 50 anos ou mais avaliados pelo CAIDE e o MoCA. Testes aplicados por alunos da 11ª fase do internato médico da UNISUL, previamente treinados, supervisionados por professores especialistas, de 09/2012 a 06/2013. Aprovação CEP/UNISUL, n° 1663.07. **Resultados:** População: 133 pessoas após exclusão de 4 pacientes por déficit sensório motor grave e 13 sem prontuários completos, 77,0% mulheres; idade 68,8±9,38 anos, média do MoCA 19,74±5,57, mediana 21,00 e moda 21,00. Média do CAIDE 9,06±1,99, mediana 9,00 e moda de 10,00. a média de risco pelo CAIDE foi de 5,86±3,60 dos quais 8 (6,0%) apresentaram risco de 1,0%, 18 (13,5%) de 1,9%, 42 (31,6%) de 4,2%, 56 (42,1%) de 7,4% e 9 (6,8%) de 16,4%. A associação do MoCA com o CAIDE foi estatisticamente significativa (p=0,000) com coeficiente beta de -0,394 (-0,162 a -0,688 95%IC). O melhor ponto de corte do CAIDE foi 8 (sensibilidade=65,8% e especificidade=63,2%, área sob a curva ROC de 68,4% (55,7 - 81,0; 95%IC) p=0,010 para identificar a população suspeita de perda cognitiva (Moca<26). **Conclusão:** Houve associação inversa significante entre os valores do MoCA e do CAIDE, indicando uma influencia de fatores modificáveis (educacionais e cardiovasculares) no estado cognitivo desta população.

**ATTENTION HEALTH OF ELDERLY NURSING** *Evani Marques Pereria; Maria Cristina Umpierrez; Maria Emilia Marcondes Barbosa*

**ABSTRACT.** This paper aims to broadly assess the physical, social and mental health in a group of elderly population, to identify risk factors for the welfare, health and safety of the elderly. The target population of this study consisted of 344 elderly residents in Guarapuava - Paraná, in four areas covered by the family health strategy. Data collection was conducted through assessment tools recommended in elderly Notebooks primary: aging and health of the elderly from the Ministry of Health (2006) which are: Geriatric Depression Scale of Yesavage, 1983, neurological examination, evaluation Nutritional Balance Scale and march Tinnet, Mini-Mental State Examination (MEM), assessment of activities of daily living (ADL) scale index karts, Apgar family. The development of this project allowed the elder care three quarters of Guarapuava-PR, as well as raising their psychosocial needs. Allowed the participation of nursing students of Scientific Initiation UNICENTRO. Contributed to the development of research in nursing, to reflect on these life stages. The identification of risk factors for the welfare, health and safety of the elderly. Planning actions to enable the elderly to participate more actively in the community, family and society, is simply work activities or through social integration. And yet the knowledge of socio-demographic, economic and health of the elderly. In the overall assessment of the elderly was detected some disorders or disabilities, it was not possible to route through the basic units for specialized service, indicating no linkage between research and health care. Demonstrated that there is need for intervention extension by the project proponents. This project was supported by the Araucaria Foundation of the State of Paraná

**AVALIAÇÃO DA FUNÇÃO COGNITIVA EM IDOSOS EM HEMODIÁLISE** *Julianna Ferreira Lima Apolinário; Mayara Maria Souza de Oliveira; Anna Carolina de Castro Araújo Lessa; Jéssika Melo Leão Bezerra; Saulo Barbosa Vasconcelos de Alencar; Kátia Cristina Lima de Petribú; Fábia Maria de Lima*

**RESUMO. Introdução:** O Envelhecimento populacional está associado ao aumento de doenças crônico-degenerativas como hipertensão arterial sistêmica (HAS) e diabetes mellitus (DM), sendo estas, fatores de risco para doença renal crônica (DRC). Este grupo populacional apresenta frequentemente declínio cognitivo, muitas vezes, rápido e pouco diagnosticado. O nefropata crônico possui alto risco de perda da função cognitiva, visto que além de apresenta comorbidades, faz uso simultâneo de vários medicamentos que podem afetar a cognição. Objetivo Geral: Avaliar a função cognitiva em pacientes idosos em hemodiálise na cidade do Recife. Material e **Método:** Trata-se de um estudo descritivo realizado com pacientes idosos em clínicas de hemodiálise da cidade do Recife vinculadas SUS. A amostra foi calculada em 53 pacientes de duas clinicas de Hemodiálise. Participaram da pesquisa indivíduos com 60 anos ou mais e em hemodiálise há mais de 3 meses, foram excluídos os que não tivessem rebaixamento do nível de consciência por delirium ou outra causa clínica que comprometesse a entrevista. Foram aplicados aos idosos, durante a sessão de hemodiálise, questionários com o perfil sociodemográfico e realizado avaliação cognitiva com MEEM. O projeto foi submetido ao CEP da Universidade de Pernambuco. **Resultados:** Os 53 pacientes idosos são em sua maioria indivíduos do sexo masculino (55%), casados ou em união estável (60%), possuem entre 4 e 7 anos de estudo (35%), com renda familiar de 1 a 5 salários (92%). Em relação ao estilo de vida, a grande maioria negou uso de tabaco (98%) e/ou álcool (90%). A doença crônica mais prevalente entre a população em estudo foi a HAS (77%) seguida de DM (39%) sendo a HAS (34%). A Diabetes Mellitus em 19% apresentou como sendo a principal doença de base que levou o idoso a nefropatia. Quanto doença psiquiátricas, 26% relatam ter tido o diagnostico de depressão. Quanto ao estado cognitivo através do MEEM, demonstraram que 59% dos idosos apresentaram declínio cognitivo.**Conclusão:** Este estudo aponta que mais da metade dos idosos em procedimento hemodialítico apresentaram declínio cognitivo e tem a DM como causa principal como doença de base.

**CAPACIDADE FUNCIONAL DE IDOSOS: ATIVIDADES BÁSICAS E INSTRUMENTAIS DE VIDA DIÁRIA** *Beatriz Regina Lara dos Santos; Marion Creutzberg; Elisandra Herr Soares; Adriana Aparecida Paz; Cristhiane De Matos Vieira*

**RESUMO.** A capacidade funcional em sua expressão máxima significa poder sobreviver sem ajuda para a realização das atividades básicas (ABVDs) e instrumentais da vida diária (AIVDs). A incapacidade funcional é a inabilidade de manter as atividades físicas e/ou mentais necessárias a uma vida independente e autônoma. Estudos populacionais mostram que cerca de 40% dos idosos, de 65 anos ou mais, necessitam de algum tipo de ajuda para realizar tais atividades. A perda da capacidade funcional está associada à predição de fragilidade, dependência, institucionalização, risco aumentado de quedas, morte e problemas de mobilidade. Essa perda gera cuidados de longa permanência e, conseqüentemente, alto custo em virtude da necessidade de assistência médica e risco de hospitalização, contribuindo significativamente para a atual crise no sistema de saúde. Quando ocorre comprometimento da capacidade funcional a ponto de impedir o cuidado de si, a carga sobre a família e sobre o sistema de saúde pode ser muito grande. È fundamental que os profissionais de saúde identifiquem o nível de capacidade funcional do idoso e os fatores relacionados a este evento, com a finalidade de propor e desenvolver possíveis intervenções. Tal princípio foi reforçado pela Política Nacional de Saúde da Pessoa Idosa (PNSPI), na qual a capacidade funcional é assumida como um novo paradigma em saúde, orientando as ações voltadas ao idoso em todos os níveis de prevenção e, de forma especial, na atenção básica, que podem ser subsidiadas pela produção de conhecimento. Estudo transversal com o objetivo de analisar a capacidade funcional dos idosos moradores da área adstrita a uma unidade básica do município de Porto Alegre. A amostra foi constituída por 197 idosos. A coleta de dados foi realizada por meio de inquérito domiciliar. Os dados foram analisados por meio de estatística descritiva e inferencial. Dos 197 integrantes da amostra, 66,5% são do sexo feminino, com idade média de 73,34 anos. Dos idosos, 52,8% são casados ou têm companheiro; 87,8% têm filhos; 28,1% possuem um cuidador. No que se refere à capacidade para realizar as atividades básicas da vida diária (AVD'S), 76,1% dos entrevistados são independentes. No que se refere às atividades instrumentais da vida diária (AIVD'S), 141 (71,6%) apresentaram independência. Os resultados mostram que os idosos, em sua maioria, têm sua capacidade funcional preservada, o que se assemelha com outras pesquisas e denota um perfil de liberdade para o idoso.

**CARING FOR AN OLDER ADULT WITH BIPOLAR DISORDER IS SUBJECT TO GREATER BURDEN THAN CARING FOR DEMENTIA** *Glenda Dias dos Santos; Rodolfo Braga Ladeira; Ivan Aprahamian; Orestes Vicente Forlenza; Paula Villela Nunes*

**ABSTRACT.** Background: Caregivers are an essential element in health care, especially in situations of chronic and disabling disorders such as Alzheimer's disease (AD). AD and Bipolar Disorder (BD) patients often need help and support in care. There are fewer studies of caregivers of elderly patients with affective disorders, especially those with BD. Even though BD represents only 1% of the population, BD elders are disproportionately affected by illness complexity and comorbidity. Therefore, studies that identify demands of care in BD elders are necessary. **Objective:** to compare burden and factors associated with it in caregivers of patients with mild to moderate AD and BD through a blind examiner. **Methods:** The study was cross-sectional and evaluated the global health (through Cumulative Illnes Rating Scale), quality of life (Whoqol), cognitive (Mini-mental State Evaluation, Verbal Fluency, Clock Drawing Test), functional (CDR and Pffefer) and psychiatric aspects (Neuropsychiatric Inventory, Geriatric Depression Scale, Beck Anxiety) of the patients. Caregivers were evaluated through the same scales in global health, quality of life, psychiatric aspects and also with the Zarit Burden Scale. **Results:** 29 elderly mild to moderate AD patients and 24 elderly BD patients and their main caregiver (who spent more time in care) were evaluated. Patients with BD had more anxious (p=0.001), depressive symptoms (p=0.002) and behavioral symptoms (p=0.003) than patients with AD. No differences were found in global health, cognition, functionality and quality of life. Caregivers of patients with BD had more burden than those with AD (p=0.004). Burden was related to patients behavioral symptoms (p=0.002) and caregiver‘s depressive symptoms (p=0.001). In caregivers depressive symptoms were associated with anxiety (p<0.001) and worse quality of life (p<0.001) and inversely related to physical activity and leisure (p=0.024; p=0.001). **Conclusions:** BD in elderly is also associated with great impact and burden in caregivers. Factors associated to burden are related mainly to neuropsychiatric symptoms of both patients and caregivers. Physical activity and leisure of caregivers seem to be protective. Ours results point toward the benefit of potentially treatable problems of caregivers through psychosocial interventions and proper medical and psychological care of depression and anxiety.

**CORO TERAPÊUTICO: A MÚSICA ATUANDO NO CAMPO COGNITIVO SOCIAL DA MATURIDADE** *Celina Amalia Vettore Maydana; Maria de Fátima Machado Brasil*

**RESUMO.** Envelhecer com qualidade é objetivo primordial do ser humano. A questão cognitiva tem sido preocupação constante, e seus mecanismos como a memória, a linguagem, a atenção e as funções executivas, afetados pelo desenvolvimento da vida, vem sendo pesquisados. Este desenvolvimento está associado a mudanças e a todos os processos adaptativos decorrentes. Para que isto aconteça plenamente e de forma natural, aptidões físicas e emocionais devem ser cultivadas a tal ponto que seu decréscimo não seja abrupto nem provoque incapacidade. Dentro destas aptidões, focamos a memória como base para estudo. Áreas cerebrais foram pesquisadas a fim de relacioná-las com diversos tipos de memórias. A música, utilizada como elemento terapêutico (musicoterapia) estimula operações físicas e mentais, com melhora significativa nos aspectos cognitivos. Este trabalho tem por objetivo, avaliar até que ponto o canto (atividade da técnica de recriação, onde o indivíduo reproduz, interpreta ou executa uma canção existente), pode influenciar positivamente a memória, e o que esta pode significar para o idoso como um sujeito ativo na sociedade. Este trabalho foi desenvolvido numa oficina de música (faixa etária: 52/90), transformada posteriormente em coral (terapêutico), no projeto USI-VIDA/USIMED, Petrópolis, RJ. A participação não inclui qualquer tipo de seleção. Os encontros semanais de noventa minutos incluem além das atividades de re-criação (musicoterapia), noções musicais de teoria/harmonia, musicalidade corporal, atividades cênicas e vocais, concentração, memorização, músicas em diversas línguas. Utilizamos também técnicas de composição, audição e improvisação (musicoterapia) através de atividades como dançar, tocar, desenvolver ritmo, história sonoro-musical. O grupo foi criado há dez anos, e a avaliação é um processo contínuo. Com base nestas atividades verificou-se uma melhora considerável no que se refere à memória, e conseqüentemente em outros aspectos cognitivos. Estas conquistas se estenderam para o âmbito familiar e social, e foram reconhecidas como fatos reais. Para os participantes representou a descoberta de que não há limites para novos conhecimentos. Com base nos pressupostos estudados, verificou-se que o canto no contexto musicoterápico, não tem por objetivo formar cantores, mas, sim, é utilizado de forma mais abrangente, atuando sobre mecanismos cognitivos (memória) como ferramenta poderosa na integração/ manutenção do indivíduo à sociedade de forma prazerosa.

**CRISES EPILÉPTICAS EM IDOSOS COM DEMÊNCIA** *André Lana Pimenta; Ana Luiza Figueiredo Campos; Danilo Ferreira Maia; Marina Buldrini Filogônio Seraidarian; Marcus Renato Castro Ribeiro; Lalleinny Franthiesca da Costa Alves; Vítor Eugênio Ribeiro; Victor Manoel Cintra*

**RESUMO. Introdução:** Uma crise epiléptica é o resultado de uma descarga elétrica excessiva, súbita e geralmente rápida de um grupo de neurônios, localizados em qualquer uma das regiões do cérebro. Sabe-se que crises epilépticas ocorrem em pacientes com demência em uma prevalência 5 a 10 vezes maior que em idosos saudáveis, e a frequência de epilepsia varia conforme a etiologia da síndrome demencial. **Objetivos:** A epilepsia e a demência são comuns em pacientes idosos e, além disso, a sobreposição entre as duas condições é comum. O presente estudo tem o intuito de abordar as crises epilépticas em idosos com demência e a problemática relacionada a seu tratamento. **Métodos:** A pesquisa bibliográfica foi realizada na base de dados Medline/Pubmed, restringindo-se aos últimos 10 anos e aos idiomas inglês e português. As palavras-chaves utilizadas foram: “Epilepsy” “Eldery”, “Dementia” e “Seizures”, limitadas ao título e ao resumo. **Resultados:** Um problema inicial em idosos com demência é a dificuldade em diagnosticar precisamente a presença e a classificação das crises epilépticas. Isso se deve em parte pela não confiabilidade dos relatos fornecidos pelo idoso e também pelo fato de os eletroencefalogramas desses pacientes raramente apresentarem descargas epileptiformes. O envelhecimento está associado à diminuição da albumina e das proteínas séricas, alteração da absorção e da distribuição das drogas, mudanças no metabolismo hepático e diminuição da excreção renal, o que pode afetar a eficácia e os efeitos adversos das drogas antiepilépticas (DAEs). Nesse aspecto, distúrbios cognitivos e comportamentais são os mais frequentes efeitos adversos vivenciados pelos idosos tratados com DAEs. Além disso, os pacientes que apresentam demência são particularmente vulneráveis a esses efeitos. Há também o risco de status epilepticus, que é mais frequente e duradouro em idosos, especialmente nos que apresentam a Doença de Alzheimer. **Conclusão:** Devido à falta de pesquisa, é limitada a informação específica do uso de DAEs em pacientes idosos com demência. DAEs com o mínimo de efeitos adversos cognitivos ou sedativos devem ser as preconizadas iniciando a terapia com doses baixas, monitorando

o nível plasmático da droga e, sempre que possível, fazendo uso da monoterapia. Cada vez mais pacientes idosos serão diagnosticados e receberão tratamento para epilepsia. Por isso, pesquisas futuras são necessárias para melhor elucidação do controle das crises em pacientes idosos com demência.

**DEPRESSION IN ELDERLY RESIDENT IN LONG PERMANENCE INSTITUTION** *Ana Luiza Pereira Rosso; Melissa Agostini Lampert*

**ABSTRACT.** Depression is the most common psychiatric illness amongst the elderly, having an estimated diagnosis in 23 to 40% of that population, reaching 54% in institutionalized ones. Regarding gender, depressive symptoms are more prevalent in women due to increased demand for health services, greater vulnerability to stressful events and a longer period of life. Thus, this retrospective-descriptive study, developed from October/2012 to January/2013, consists in an epidemiological analysis of the incidence and of the profile of depressed elderly residing in Long Permanence Institution (LPI) “Lar das Vovozinhas”, in Santa Maria, RS. It were analyzed the following variables of 142 elderly: age; environment (the place which they stay most of the time); confirmed depression; other psychiatric disorders, such as bipolar affective disorder-BAD, schizophrenia, mental retardation, epilepsy and psychosis; organic diseases (hypothyroidism, stroke, inability to communicate, Parkinson, Alzheimer and other dementias). After that analysis, it was made statistics with the use of the program SPSS13.0. The results were: average age of the sample, 74 years (SD=9.365); 59.1% of all elderly women have some psychiatric disorder, considering the most common as depression (32.3%, with an average age of 75.3 years; SD=9.57), BAD (15.4%) and mental retardation (15.4%). In the LPI, the place with the larger number of elder with depression is ward 4 (41.3% of total), followed by ward 2 (26%), destined to women requiring intensive care; ward 1 (23,9%), to women without need for intensive care); and ward 3 (8.6%), in which stay women with behavior change and need vigilance. It was concluded that 52,1% of depressed elderly have other psychiatric or organic disorder that may have some relation with depression; the most frequent, hypothyroidism (13%) and mental retardation (10.8%). Those data are the first stage of a project that aims to check initial depression in elder living in a LPI and their relationship with other psychosocial factors. From the results, it will be developed a second step, which involves the application of questions related to psychosocial factors. With this, it will be possible to suggest measures that may help in prevention and health promotion involved with the Mental Health of institutionalized elderly providing them quality of life.

**DEPRESSIVE SYMPTOMS AND NUTRITIONAL STATUS IN LONG TERM CARE OLDER ADULTS** *Patrick Alexander Wachholz; Flávia Cristina Severo Grando*

**ABSTRACT.** Depression is highly prevalent in institutionalized older adults, but little is known about its risk factors in long term care facility (LTCF). In some studies, age greater than 85 years and hearing impairment appeared to be protective, and dementia emerged as an independent risk factor. **Objectives:** This study aims to investigate associated risk factor for depressive symptoms in a sample of Brazilian aged living in a LTCF in Curitiba, PR. **Methods:** This is a cross-sectional study. Out of 27 elderly living in the facility in 2010, 13 were included, since they were able to consent with the participation in the study (Cognitive Dementia Rating≤1) and to answer the Yesavage Geriatric Depression Scale (GDS-15). Anthropometric measures, Charlson score, Frailty score, drugs in use, functional abilities (Barthel index) and time since institutionalization, along with age, were analyzed as risk factors. Bivariate analyzes were applied to identify the most significant variables. Kolmogorov-Smirnov test confirmed the normal distribution of the variables, and only the normal ones were included in a Linear Regression model, GDS-15 as the dependent variable, significance set at p≤0.05. **Results:** The mean age of the sample was 85.15 (±8.25) years, 61.5% were female. Four older adults scored 6 or more at GDS-15. The mean MAN score was

21.54 (±4.58), 46.2% were at risk of malnutrition and 15.4% were malnourished. Mean living period in the LTCF was 4.73(±2.68) years. The majority of the sample (72.17%) was functional independent. Bivariate analyzes identified MAN as the only variable significantly correlated to GDS-15 (p=0.024). The linear regression model confirmed the dependency of this unique variable (p=0.038). **Conclusion:** In a small sample of institutionalized older adults, better nutritional status independently correlates to less depressive symptoms. Although other important risk factors, as cognitive function, were not analyzed, is significant to identify this correlation.

**DOENÇA DE PARKINSON E ALFA-SINUCLEINA: UMA REVISÃO BIBLIOGRÁFICA** *Marcela Geisa Becegatto; Sheila Cristina Cecagno Zanini; Luana Taís Hartmann Backes; Taís Romeu Ximendes; Telma Elita Bertolin*

**RESUMO.** A pesquisa bibliográfica foi realizada a partir da busca nos periódicos Capes e no PubMed o qual abrange mais de 22 milhões de citações para a literatura biomédica do MEDLINE, periódicos de ciências naturais e livros on-line. As doenças neurodegenerativas geram dúvidas a respeito de sua origem, o papel das vias conservadoras, o envelhecimento do cérebro e o estresse oxidativo. A doença de Parkinson (DP) é neurodegenerativa, crônica e progressiva, associada a uma disfunção do movimento e envolve fatores ambientais e genéticos. Por isso uma caracterização específica dos fenótipos clínicos e a sua correlação com as manifestações pré-diagnósticas podem ser pontuais. Caracteriza-se por uma perda seletiva de neurônios dopaminérgicos na substância nigra e pela presença de corpos de Lewy. Quando diagnosticada a DP já pode ser considerada irreversível, pois quando começam a surgir os sintomas a substância nigra já perdeu 60% dos neurônios dopaminérgicos e o conteúdo de dopamina transmitida é 80% inferior. A DP não pode ser simplesmente considerada uma doença nigroestriatal, muito embora os neurônios dopaminérgicos sejam alvos preferências. É caracterizada pelo acúmulo e agregação da alfa-Sinucleina (a-sin) que é uma proteína neuronal, pré-sináptica que participa como modulador negativo na neurotransmissão da dopamina. A a-sin aparece como molécula-chave na patogênese das sinucleinopatias, todavia sua relação com a disfunção mitocondrial não está clara. A a-sin e os sistemas proteolíticos intracelulares interagem numa cascata de autoalimentação que leva a neurodegeneração, estratégias que atuem na segmentação das vias de degradação específicas podem ser alternativa. Sobre o papel da glia nas sinucleinopatias há controvérsias entre o clínico e os resultados de pesquisas acerca das terapias alvo da disfunção glial. A produção, distribuição, modificação e degradação da a-sin são essenciais para as funções neuronais. A DP é comumente associada com a exposição a substâncias tóxicas. Acredita-se que a morte dos neurônios ocorre devido ao estresse oxidativo, danos em moléculas sinalizadoras, mudanças na regulação da expressão das proteínas pró-apoptóticas e mau funcionamento dos caminhos neuronais. O envelhecimento e a DP apresentam características comuns interconectadas com a geração de radicais livres. Os níveis de lipídeos e as atividades metabólicas são vulneráveis aos efeitos deletérios dos radicais livres, devido ao alto consumo de oxigênio pelos neurônios.

**EFEITOS DA TERAPIA NÃO FARMACOLÓGICA NO TRATAMENTO DE IDOSOS PORTADORES DE DEMÊNCIA: REVISÃO INTEGRATIVA DA LITERATURA** *Delvanita de Souza Santos; Gislaine Desani da Costa; Rosely Almeida Souza*

**RESUMO.** A demência é caracterizada por múltiplos comprometimentos das funções cognitivas. Os tratamentos utilizados para tratar os sintomas das demências são a terapia farmacológica e a não farmacológica. Para melhor compreensão dos efeitos das terapias não farmacológicas, realizou-se uma revisão integrativa com o objetivo de identificar as publicações científicas que abordam a terapia não farmacológica no tratamento de idosos portadores de demência e apresentar um panorama dos efeitos daquelas no tratamento desses idosos. A busca ocorreu em julho de 2012. Consultaram-se as bases eletrônicas de dados: LILACS; IBECS; MEDLINE; Biblioteca Cochrane; e o diretório eletrônico SCIELO. Utilizaram-se os seguintes descritores: “Demência”, “Idoso”, “Reabilitação” e “Equipe de Assistência ao Paciente”. Os critérios de inclusão foram: estudos que abordaram como idosos os indivíduos com idade igual e superior a 60 anos; estudos em que os idosos com demência foram submetidos a qualquer tipo de tratamento não farmacológico; estudos publicados entre 2002 e 2012; e, por fim, estudos em qualquer idioma. O levantamento bibliográfico identificou 124 publicações e, destas, foram selecionadas seis, por atenderem aos critérios de inclusão no estudo. Os achados revelaram predomínio de estudos com delineamento experimental, publicados no ano de 2011, em sua maioria na área da Neurologia. Entre os principais tratamentos não farmacológicos utilizados destacaram-se: reabilitação cognitiva, treinamento cognitivo, programas de exercícios físicos, orientação e suporte psicológico aos familiares e cuidadores, terapia ocupacional, terapia para fala, orientação para realidade e terapia de reminiscência. Quanto aos efeitos das terapias não farmacológicas utilizadas no tratamento dos idosos portadores de demência, todos os estudos evidenciaram benefícios, tais como: melhora nos aspectos cognitivos, funcionais, psicológicos e comportamentais; diminuição e melhora dos sintomas depressivos e redução da ansiedade. Houve ainda, redução do estresse, ansiedade e depressão; redução da sobrecarga e melhora na qualidade de vida do cuidador e dos familiares. Conclui-se que a terapia não farmacológica traz benefícios não só ao idoso portador de demência, mas também, aos seus respectivos cuidadores e familiares.

**ESTRUTURA DE FUNCIONAMENTO DO AMBULATÓRIO DA MEMÓRIA DA UNIVERSIDADE DO SUL DE SANTA CATARINA** *André Junqueira Xavier; Gabriela Coutinho Cavalieri; Leonardo Da Silva Lima; Pâmela Nogueira Da Silva Vilela; Rafael Balestreri Trevisol; Vinicius Carriero Lima*

**RESUMO. Introdução:** O Ambulatório da Memória (AMemo) foi criado em setembro de 2012 dentro do Ambulatório Médico de Especialidades da UNISUL, Pedra Branca, para atender a crescente demanda em virtude da alta incidência e prevalência de doenças que afetam a capacidade cognitiva. **Objetivos:** diagnóstico, triagem, prevenção, acompanhamento de déficit cognitivo e demências em adultos e idosos. **Métodos:** Equipe composta por alunos da 11ª fase do Internato Médico supervisionados por geriatra. Atendimento: via encaminhamento da atenção básica, geriatria, neurologia, psiquiatria ou por demanda livre de pessoas com queixa de memória. Serviço conveniado com o SUS/Prefeitura de Palhoça - SC. Referência e contra referência para cardiologia, endocrinologia, neurocirurgia e reumatologia, psicologia e fisioterapia. Acoplada ao AMemo, a “Oficina da Lembrança” (OL) funciona desde 2006 na UNISUL como extensão e pesquisa e visa estimular e reabilitar a função cognitiva de adultos e idosos hígidos, com Transtorno Cognitivo Leve e até quadros leves e moderados de demências. Este trabalho é realizado por meio do uso de computadores (laboratórios com 20 a 30 PCs), internet e atividade física (pista de caminhada coberta) e equipamento de realidade virtual (Xbox^®^, Kinect^®^). A OL conta com três horários semanais, 1,5 hora/oficina. **Resultados:** De set/12 a jun/13, foram acompanhados 198 pacientes (348 consultas). Sendo 13% até 59 anos, 68% de 60 a 79 anos, 19% 80 anos ou mais. Sem alteração cognitiva 21,9%, diagnóstico clínico de TCL 58,2%, e 15,8% diagnóstico clínico de demência. Passaram pelo AMemo, 50 internos, receberam formação quanto ao atendimento, acompanhamento e tratamento medicamentoso de problemas cognitivos, bem como aplicação e interpretação dos instrumentos de rastreio GDS15, CDR, Diabetes Risk, Risco Nutricional, Índice tornozelo-braquial, MEEM, BOMFAQ/OARS, MOCA e CAIDE. Também realizaram treinamento em orientação de pacientes com diagnóstico clínico de demência, cuidadores e familiares individualmente e por meio de grupo de apoio à pessoa dementada (cada 5 semanas). Na OL, desde 2006 foram atendidos 289 pacientes (acompanhados no estudo de coorte) por 120 monitores, atualmente 31 pacientes frequentam a OL atendidos por 33 monitores, estudantes treinados de primeira a oitava fase do curso de Medicina. **Conclusão:** O Ambulatório da Memória é uma nova alternativa para saúde cognitiva da população, formação médica e produção acadêmica.

**GRUPO DA MEMÓRIA: O IMPACTO DAS ESTRATÉGIAS MNEMÔMICAS NA QUALIDADE DE VIDA DO IDOSO** *Maria Tércia Barroso Pereira Malta; Regina Celia Marques de Melo; Maria Lucia Carneiro dos Rios Ferreira; Marta Cristina Ayres Neves Porto; Marcia Lima; Helayne da Costa Coelho; Claudiane Monsores de Sá; Renata Soares dos Santos*

**RESUMO. Introdução:** Estudos epidemiológicos mostram que idosos hipertensos, diabéticos e deprimidos apresentam maior risco de desenvolver demências e declínios cognitivos; intervenções sobre a memória podem contribuir para a promoção da saúde e autonomia dos mesmos. O bom funcionamento da memória é vital para que estes possam continuar a viver de forma independente. **Objetivo:** Apresentar a vivência da estimulação cognitiva desenvolvida em um grupo de terceira idade, em um Hospital no Rio de Janeiro. **Metodologia:** O Grupo da Memória é uma iniciativa do Programa Interdisciplinar de Promoção à Saúde e Qualidade de Vida do Idoso - Grupo Renascer, do Hospital Universitário Gaffrée e Guinle/UNIRIO. É composto por 69 idosos, sem diagnóstico de demência e que apresentam queixas subjetivas de déficit de memória. Quanto às patologias: 75,9% são hipertensos; 25,9% diabéticos e 17,2% apresentam queixas de depressão. Os encontros são semanais sendo a programação preventiva e lúdica. A equipe é formada por profissionais e alunos das áreas da psicologia, fisioterapia, arte-educação, enfermagem e nutrição. São utilizadas técnicas e exercícios individuais e em grupo para estimular a memória, percepção, atenção, linguagem e funções executivas. **Resultados:** O grupo em sua maioria é composto por mulheres 94,2%; 46,4% com idade entre 70 e 79 anos; 47,8% têm

o ensino fundamental incompleto; 58% avaliaram sua memória como ruim antes do ingresso no grupo e 94,2% avaliaram a memória como boa após ingressarem no mesmo; 94,2% utilizam estratégias aprendidas no grupo que auxiliam no desenvolvimento de suas atividades de vida diárias - AVD: lembretes na geladeira, associações de nomes, lista de compras, uso de agenda, anotações de recados e reserva de lugar especifico para objetos; 85% verificaram que estas estratégias contribuíram bastante para melhorar a realização de suas atividades diárias. **Conclusão:** O trabalho desenvolve não só as funções cognitivas, mas também estimula o idoso a procurar novas estratégias para a vida diária, eleva a autoestima, desenvolve habilidades e o ajuda a manter sua independência social e pessoal. O grupo oferece subsídios para que o idoso possa entender e desenvolver os processos envolvidos na memória, contribuindo para a melhora e/ou manutenção de sua reserva cognitiva. O Grupo da Memória além de ser um espaço de treinamento é também um espaço de encontro, onde são formadas as redes sociais, tão importantes para a vida do idoso quanto o aprendizado adquirido.

**HABITS OF LIFE OF CAREGIVERS OF INSTITUTIONALIZED ELDERLY** *Rosane Paula Nierotka; Camila Malesza; Samila Livinalli; Marilene Rodrigues Portella*

**ABSTRACT.** The longevity exposes the onset of chronic degenerative diseases, making the elderly increasingly dependent care. A family support ineffective care refers to the condition of long stay institutions for the elderly (LTCF). Care professionals, along a trajectory can change their lifestyle habits due to the workday. **Objective:** Identify the habits of life of caregivers of institutionalized elderly. **Method:** A descriptive exploratory eighteen caregivers nurses, fourteen ILPI a municipality north of the state of Rio Grande do Sul We used a self-administered questionnaire and analyzed using the statistical package, this study was approved by the Committee of Ethics Committee of the University of Passo Fundo Protocol No. 393/2011. **Results:** The sociodemographic characteristics, the majority were female, single (a), with the predominant age thirty to thirty-nine, and twelve nursing technicians and six nurses. Mostly the work performed in the LTCF, was less than a year, with a weekly schedule forty hours or more and a fee of two minimum wages. About the habits of life, half physical exercises, physical activities among the predominant is the walking, weight training, dancing, cycling, swimming and football. The majority (72.2%) of caregivers reveals occupation with leisure and has no contact with smoke or drink alcohol, however (16.7%) were smokers and another equal portion makes use of alcoholic beverages. How to contact with harmful agents in the workplace, the majority (61.1%) of the participants signaled positive, and 37.8% are attributable to sharps, 33.3% blood and fluids and the same percentage for repetitive tasks and repetitive and biological also toxic and 16.7% sharp. The highlight significant (44.4%) was for exposure to violence and aggression. **Conclusion:** The findings of this research show how professional caregivers, as in the case of working in LTCF need attention. Measures to encourage improvements in lifestyle, during or after the care activity, such as greater adherence to physical activity. Recommend to continuing education courses, with a view to the preparation of professionals to work due to the conditions of exposure to triggers for occupational injuries.

**HEALTH BEHAVIORS AND SOCIOECONOMIC CHARACTERISTICS OF ELDERLY LIVING IN RURAL AREAS OF MINAS GERAIS** *Nayara Paula Fernandes Martins; Darlene Mara dos Santos Tavares*

**ABSTRACT.** Adoption of health behaviors such as physical activity, not smoking and not drinking, prevents or postpones comorbidities onset and make it possible to achieve a healthier aging process. **Objective:** To characterize elderly according to socioeconomic characteristics, health behaviors and number of comorbidities; to check socioeconomic factors and number of morbidities associated with health behaviors among the elderly. **Method:** Eight hundred fifty elderly residents in rural areas of Uberaba-MG have participated of the present study. We have used the Brazilian Functional Evaluation Questionnaire and also, for health behavior, we have used a questionnaire developed by the group of researchers. We have conducted Simple Frequency Distribution for categorical variables and Bivariate Analysis was conducted preliminary to identify associated factors; tests were considered significant when p<0.1. Associations with p<0.05 were included in the logistic regression model. We used the 17 version of Statistical Package for the Social Sciences (SPSS) for analysis. The project was approved by the Ethics Committee of the Federal University of Triangulo Mineiro, protocol No. 1477. **Results:** Prevalence of male elderly with 60-70 years, living with a partner, with 4-8 years of schooling, income of monthly individual minimum wage and 1-4 morbidities. Regarding health-related behaviors, most seniors reported no physical activity, not smoking nor drinking. The physical activity showed no statistically significant association. Being a female (β=2.106) and not having a partner (β=1.681) remained as a predictor of smoking and being a female (β=0.368) as a predictor of alcoholism. **Conclusion:** It is up to health professionals to develop actions that encourage physical activity and prevention of comorbidities and complications.

**HISTORY OF FALL IN THE ELDERLY: TRACKING COGNITIVE** *Gilson de Oliveira Carol Júnior; Marília Gino Gonçalves; Alexsandra Maria Siqueira Campos de Carvalho; Fabia Maria Lima; Marilia Siqueira Campos*

**ABSTRACT.** Cognitive impairment can affect social or occupational functioning and represent a delay in relation to a previously higher level of cognitive functioning, and this can compromise the functional capacity of the elderly. Within the functional capacity, fragility is considered one of the greatest challenges for health professionals in aging societies, as the fragility makes the elderly more vulnerable to decline and consequently the other adverse events such as hospitalizations, complications, dependency, institutionalization and death. **Objective:** To correlate with cognitive impairment in elderly people with and without a history of falling living in the city of Recife. **Method:** This is an observational study, cross-sectional, descriptive quantitative approach, data were collected through interviews sociodemographic, variable drop, Mini Mental State Examination (MMSE) and GDS 5, after the signing of the informed consent, conducted from July 2012 to October/2012. The sample consisted of 126 individuals over 60 years of Political-Administrative Region three (AOR-6) in Recife, chosen as the study site. This study was approved by the Ethics Committee on Human Research of the University Hospital Oswaldo Cruz by and filed under number 04273412.9.0000.5192. **Results:** From the analysis of the interviews, there are females (76.8%) was more prevalent, age 70-79 years (43.9%), education that prevailed was illiterate (36%). As for the morbidities and medications, the elderly had to drop the prevalence (33.3%) higher morbidity /=3 (64.5%) and took 0-3 medications and multiple drugs was 35.5% (≥4 ). Regarding individuals who fell (63%) had depressive symptoms with scores >2, it was also observed that the elderly in total (70.4%) had MMSE changed. **Conclusion:** on the foregoing, it is identified that the factors, number of comorbidities, number of medications, education, Mini altered examination and depressive symptoms, contribute to cognitive impairment, making the elderly more vulnerable occurrence of falls. Key words: Elderly frail elderly; cognition.

**INVESTIGAÇÃO DO RISCO DE DISFAGIA E SUA RELAÇÃO COM O ESTADO NUTRICIONAL E SÍNDROME DEMENCIAL DE IDOSOS INSTITUCIONALIZADOS** *Fabiana Cordeiro Juliani; Vera Silvia Frangella*

**RESUMO.** Distúrbios da deglutição, decorrentes ou não da presença de neuropatias, são observados frequentemente na população idosa, especificamente a disfagia. **Objetivo:** Investigar a ocorrência do risco de disfagia em idosos institucionalizados e correlacioná -la ao risco nutricional nesta população. Correlacionar o estado nutricional da amostra com os valores da espessura do músculo adutor do polegar (EMAP) e da força de preensão palmar (FPP). **Metodologia:** Após aprovação pelo Comitê de Ética em Pesquisa, avaliaram-se idosos institucionalizados de ambos os sexos com idade ≥80 anos. Para a detecção do risco nutricional aplicou-se a Mini Avaliação Nutricional (MAN). Para o diagnóstico nutricional, consideraram-se as variáveis antropométricas: IMC (índice de massa corporal), DCT (dobra cutânea do tríceps), CMB (circunferência muscular do braço) e AMB (área muscular do braço), e a CP (circunferência da panturrilha). Também foram mensuradas a EMAP e a FPP. Por meio de formulário específico verificou-se o risco de disfagia. A análise dos dados deu-se pelo teste exato de Fisher (p<0,05) e de Correlação de Pearson (r). **Resultados:** A amostra compôs-se por 31 idosos com média de idade de 87,5±5,65 anos, sendo a maior parte mulheres (90,3%). A MAN revelou que 37,8% da amostra apresentavam-se em risco de desnutrição. O risco de disfagia foi identificado em 87,1% dos idosos e sua relação com o risco nutricional não se mostrou significativa (p=0,27). Entretanto, o risco de disfagia apresentou significativa relação com o diagnóstico de síndrome demencial de origem primária (p=0,0003). O IMC indicou maior frequência de eutrofia (58,1%), seguida pelo excesso de peso (29%) e pelo baixo peso (12,9%). A avaliação da DCT indicou que 83,9% dos idosos possuíam excesso de reserva adiposa. Não se observou comprometimento nas medidas de CMB e AMB, mas se detectou depleção na CP de 48,4% da amostra. A média da EMAP foi de 10,5±2,05mm e sua correlação com o IMC mostrou-se negativamente fraca (r= -0,12). Dentre os idosos que se mostraram aptos para a realização do teste de FPP (85,9%), a média obtida foi de 4,1±4,97kg/f e esta medida apresentou fraca correlação positiva com o IMC (r=0,29). **Conclusões:** O risco de disfagia esteve presente independentemente do estado de risco nutricional, relacionando-se mais com o diagnóstico de síndrome demencial de origem primária. As medidas de EMAP e a FPP apresentaram fraca correlação com o estado nutricional dos idosos definido por meio do IMC.

**JOGO PATOLÓGICO -RELATO DE CASO** *Clarissa De Angelis dos Santos; Gabriella Polastri Stiilpen; Leonardo Vinicius de Andrade; Fabiana Souza Máximo Pereira; Marco Túlio Gualberto Cintra; Edgar Nunes de Moraes; Leonardo França Antunes*

**RESUMO. Introdução:** O jogo patológico é caracterizado pela perda do controle de jogar, que pode levar a consequencias negativas como problemas na família, no trabalho, relações sociais e financeiras. Este quadro é pouco prevalente em nosso meio, mas relativamente comum nos EUA, principalmente em jovens. Estudos em grupos demográficos identificaram alguns fatores de risco para desenvolvimento do jogo patológico como idade, sexo (masculino), descendentes de africanos, pacientes em tratamento de doenças mentais, uso abusivo de tabaco, álcool e drogas ilícitas, história familiar positiva e baixo nível sócio-econômico. Objetivo e **Métodos:** Descrever 2 casos de jogo patológico em idosos atendidos em um serviço de geriatria de Minas Gerais e realizar revisão bibliográfica sobre o tema. **Resultados:** Caso 1: paciente de 72 anos, sexo feminino, feoderma, viúva, analfabeta, independente para atividades de vida diárias e com cognição preservada, que evoluiu com compulsão por jogos de azar, perdas financeiras graves e desajuste familiar. Caso 2: paciente de 75 anos, sexo masculino, 7 anos de escolaridade, divorciado, independente para atividades de vida diárias com comprometimento cognitivo leve, apresenta compulsão por jogos de azar com conseqüente perda financeira há 30 anos. Em ambos os casos o diagnóstico foi estabelecido após preenchimento de 6 critérios dos 10 descritos pelo Diagnostic and Statistical Manual of Mental Disorders (DSM - IV). A paciente encontra-se em tratamento psicoterápico, já o segundo idoso foi encaminhado a psiquiatria, mas se recusou iniciar tratamento medicamentoso proposto. **Conclusão:** O jogo patológico é uma doença com importante impacto sócio-familiar, entretanto é frequentemente subdiagnosticado. Até o momento, existem poucos estudos que demonstram tratamento medicamentoso eficaz, principalmente com fluoxetina. Preconiza-se a psicoterapia, mas geralmente os pacientes são pouco aderentes.

**LEVEL OF FUNCTIONAL INDEPENDENCE IN ELDERLY PATIENTS WITH ALZHEIMER'S DISEASE** *Rosane Paula Nierotka; Fátima Ferretti; Clodoaldo Antônio de Sa; Vanessa Corralo Borges; Ana Paula Gauer; Maite Fiabani*

**ABSTRACT.** Alzheimer's disease (AD) is a neurodegenerative disease of character and deleterious evolution that affects 9% of people over 65 years. The DA agrees to physical, mental and social, cognitive and neuropsychiatric manifestations causing that result in progressive disability and disability of the individual. Cognitive decline inherent to the disease is related to loss of functional capacity, ie, to perform instrumental activities of daily living (IADL) and basic activities of daily living (BADL) **Objectives:** To evaluate the functional independence of older adults diagnosed with Alzheimer's and indicative relate this level with years of symptoms according to sex and age. **Methods:** Study participants were 50 individuals diagnosed with Alzheimer's callsign, comprising 36 females and 14 males (age 80.0±7.21 and 77.35±8.89 years, respectively). The data collection instruments used were the Mini Mental State Examination (MMSE), Barthel protocol, Lawton and Time UpandGo (TUG). **Results:** The results showed a higher MMSE cognitive impairment in the group with more than nine years of symptoms in the group with up to four years (p=0.013). As for functional evaluation protocol from Barthel and evaluation of mobility (TUG), there were no differences (p>0.05) between the groups analyzed. Regarding the analysis of functional capacity as assessed by protocol Lawton, it was demonstrated that the groups 5-8 years and with over 9 years of symptoms, functional impairment had significantly higher (p<0.05) than the group with 4 years of sintomas.O time symptoms correlated positively (p<0.05) with the cognitive ability and funcional. **Conclusion:** The study hypothesis was confirmed, as the results showed that the level of functional independence of older people with Alzheimer's diagnostic indicator associated with disease progression, whereas the years symptoms. Those who had more than nine years of symptoms were the ones with the lowest score in the protocols used.

**LIFE SATISFACTION AND BODY MOBILITY IN ELDERLY** *Ralf Braga Barroso; Elaine Andrade Moura; Thamara Cunha Nascimento Amaral; Cláudia Helena Cerqueira Mármora*

**ABSTRACT.** The aging process causes some cellular modifications, principally in muscular tissue, that may cause decrease in muscular tonus, protect reflex alterations and equilibrium problems, harming the body mobility. Thus, the inferences of the aspects of mobility are directly aimed to the health of bone structures, neural and muscle, which in third age presents itself in a more compromised. The elderly who practice regular physical activity reduce the developing of chronic diseases, that implies in a lower sensitivity towards the problems involving mobility. In community centers, seniors remain active through various activities and social engagement, aiming to maintain their functional capacity, by providing a more positive internal sense of the aging process. **Objective:** The objective of this study was to verify the existence of correlation between life satisfaction and body mobility in elderly who attends community centers in Juiz de Fora, Minas Gerais, Brazil. **Methods:** We apply two instruments, the Life Satisfaction Index for Third Age (LSITA) and the Timed Up and Go Test, in order to assessment the life satisfaction and body mobility, respectively. The elderly were recruited from Community Centers in Juiz de Fora, Minas Gerais, Brazil. The statistical analyze was done using the software SPSS 20.0 and, in order to verify the presence of correlation, was used the Pearson coefficient correlation (p<0.05). We used the Kolmogorov-Smirnov test as well to verify the variables' normality pattern. Every participant signed the Consents Term and this research was approved by Ethic Committee in Human Research of Federal University of Juiz de Fora in the opinion 238,469/2013. **Results:** We assessment 97 elderly with a mean age 72.35 years (SD±4.88), which 8 was male and 89 female. The average values of life satisfaction was 102.07 (SD±17.82) and physical mobility was 7.79 (SD±1.73). The study variables were normally distributed according to the Kolmogorov-Smirnov test. By Pearson coefficient correlation was not observed a correlation statistically significant (r=0.247; p=0.119). **Conclusion:** In general, the elderly of community centers are satisfied with life and have good body mobility, providing them a healthier aging. However, body mobility seems not to interfere in the life satisfaction of these elderly.

**MEDICATION ADHERENCE IN FRAIL ELDERLY OUTPATIENTS WITH DEMENTIA** *Thiago Vinícius Nadaleto Didone; Eliane Ribeiro*

**ABSTRACT.** Since drug therapy in the elderly is complex and long-term and aged people commonly present some level of impairment and disability, medication adherence tend to decrease with age. Cognitive function is a key factor associated with medication adherence and professional or caregiver assistance may be necessary to maintain correct drug use. This study aims to analyze frail elderly outpatients aged 80 years or over diagnosed with dementia. The study is cross-sectional and is being conducted at the Ambulatory of Frailty of the University Hospital of the University of São Paulo (AF-UH). It is being based on information collected through an interview conducted with the patient or its caregiver. Medication adherence is assessed by the proportion of the prescribed drugs used in concordance with the prescription. Here it is presented the results of a pilot study. Thirty patients were included in the pilot study of which 23 (76.7%) were female and 7 (23.3%) males. The mean(SD) age, number of dwelling relatives, living children and prescribed drugs was, respectively, 86(5) years, 3(2), 3(2) and 6(3). The AF-UH consultation is the only regular physician encounter for 60.7% of the patients. Out of 30 patients, 5 (16.7%) live alone. Medication is a caregiver responsibility in 22 (73.4%) patients; the others (26.6%) self-administer their medicines. 13 (43.3%) of patients regularly use at least one drug not prescribed. Dementia was present in 8 patients all of which have a caregiver responsible for the management and,or the administration of the medicines; on the other hand, only 4 of the 22 nondemented patients (18.2%) have assistance of a caregiver (p<.001). The mean(SD) number of prescribed drugs was higher in nondemented patients [6.5(2.4)] than in those with dementia[3.5(2.3)] (p=.004). Educational level was similar between caregivers and patients (p=.503) as well as between caregivers of demented and non demented patients (p=.582). Among patients without dementia, those with caregiver assistance presented the same mean(SD) medication adherence [0.93(0.14)] than those without it [0.78(0.28)] (p=.305). When compared to nondemented patients without caregivers, demented patients showed higher medication adherence [1.00(0.00)] (p=.013) since all of them used their drugs as recommended. The lower number of prescribed drugs and caregiver assistance seem to play an important role in the adherence of pharmacotherapy of demented patients in the studied population.

**MÉTODO INTERMED: COMPLEXIDADE ASSISTENCIAL E A RELAÇÃO COM DECLÍNIO COGNITIVO, HUMOR E INDEPENDÊNCIA DO IDOSO** *Henrique Salmazo da Silva; Maria Helena Silveira; Beatriz Aparecida O. Gutierrez*

**RESUMO.** **Objetivo:** Avaliar a associação entre declínio cognitivo, sintomas depressivos e o grau de complexidade assistencial biopsicossocial e no sistema de saúde de idosos hospitalizados. **Métodos:** Este estudo foi quantitativo, descritivo, exploratório e transversal, com amostra de 202 hospitalizados na Clínica Médica do Hospital Universitário (HU). Foi aplicada a Escala INTERMED, que classifica a complexidade assistencial considerando domínios biopsicossociais e de saúde. Aplicou-se a escala Katz para classificar o idoso como independente, semi-dependente ou dependente. A avaliação de humor foi realizada por meio da Escala de Depressão Geriátrica (EDG), com ponto de corte >6 pontos. O rastreio cognitivo foi realizado com o Mini Exame do Estado Mental (MEEM) utilizando pontuação equivalente a um desvio padrão abaixo das medianas propostas por Brucki et al (2003). **Resultados:** 85,1% dos entrevistados apresentaram alguma relevância na complexidade assistencial, demandando uma rede de cuidados em saúde. Elevada pontuação na EDG (p.0,00) e menor pontuação no MEEM (p.0,00) foram associadas à maior complexidade assistencial. Menor complexidade e pontuação no INTERMED foram evidenciadas em idosos independentes. Houve diferenças entre o desempenho no MEEM e o grau de dependência, diferenciando-se entre independentes, semidependentes e dependentes. **Conclusão:** O INTERMED apresentou elevada associação com o estado cognitivo global, grau de independência e humor, indicando possibilidades favoráveis de sua utilização na prática com idosos hospitalizados.

**MORBIDADE DEPRESSIVA AUTORREFERIDA EM IDOSOS DE UMA UNIDADE GERONTO-GERIÁTRICA** *Rita de Cássia Hoffmann Leão; Mário Roberto Agostinho da Silva; Catarina Magalhães Porto; Jéssica Patricia Sales do Nascimento; Mariana Amorim Amaral Menezes; Jaime Roberto Tavares de Lima*

**RESUMO. Introdução:** Diferentes estudos descrevem a sintomatologia depressiva como uma das principais queixas em unidades de atenção ao idoso. Enquanto doença afetiva, a depressão pode ser confundida com um sentimento de tristeza decorrente de uma “perda” ou ser desencadeada por outras morbidades dificultando o diagnóstico, como nas demências de Alzheimer, prolongando o sofrimento do paciente. **Objetivo:** Levantar percentual de idosos com queixa de humor depressivo referido na primeira consulta em unidade geronto-geriátrica. **Metodologia:** Trata-se de estudo descritivo, quantitativo de corte transversal, a partir de dados secundários registrados em prontuário multiprofissional do atendimento de 227 idosos, com faixa etária entre 60 e 95 anos, cadastrados e acompanhados no Núcleo de Atenção ao Idoso - NAI/PROIDOSO/PROEXT/UFPE, no período de janeiro de 2010 a junho de 2013. Os atendimentos são realizados a partir de agendamento prévio, com duração mínima de 45 minutos, garantindo ao paciente retorno e encaminhamento a Equipe da Unidade. **Resultados:** Dos 227 idosos avaliados, 68 idosos (30%) referiram, entre outras queixas, problemas relacionados a fatores psicológicos e psiquiátricos. Quanto ao gênero, 32% eram do sexo masculino e 68% feminino. A faixa etária foi distribuída em 53% entre 60 e 69 anos, 31% entre 70 e 79 anos e 11% com 80 e mais anos. As queixas mais frequentes foram: humor depressivo 49%, ansiedade 18%, ansiedade e depressão 25%, fobias 7% e outras 1%. Apenas 46% viviam com companheiro(a), os demais estavam solteiros, separados ou viúvos. Menos de 10% possuía assistência privada à saúde. Do ponto de vista socioeconômico não apresentaram similaridades, indo de pessoas sem renda àquelas com rendimentos superiores a cinco salários mínimos. Os casos em que houve confirmação do diagnostico foram reencaminhados para a Equipe especializada em transtornos afetivos do NAI ou a unidades de referência para orientação e tratamento. **Conclusão:** Os dados apresentados indicam que as doenças da esfera psicológica e psiquiátrica nestes idosos aparecem como uma das principais causas de morbidade nesta unidade de saúde, corroborando com estudos anteriores. Durante o envelhecimento, o indivíduo enfrenta várias situações que podem alterar seu humor e, consequentemente, sua qualidade de vida, o diagnóstico diferencial e o tratamento adequado das doenças afetivas, principalmente da depressão, requerem treinamento da equipe possibilitando a identificação desses transtornos.

**O CONHECIMENTO DE MÉDICOS E ENFERMEIROS REFERENTE À ASSISTÊNCIA AO PORTADOR DE DEMÊNCIA E SEUS FAMILIARES: REVISÃO INTEGRATIVA DE LITERATURA** *Daniel Francisco Antão; Gislaine Desani da Costa; Rosely Almeida Souza*

**RESUMO.** A demência é uma disfunção cerebral caracterizada por deterioração intelectual adquirida e persistente, que gradativamente compromete funções em três esferas: cognição, função e comportamento. Cuidar de pessoas com demência representa uma sobrecarga para as famílias. O reconhecimento dos sintomas da doença pode diminuir a tensão decorrente do cuidado e contribuir para a qualidade de vida do paciente e seus familiares. Realizou-se uma revisão integrativa da literatura orientada pela indagação: “como está o conhecimento de médicos e enfermeiros que concerne ao atendimento do portador de demência e seus familiares”? Os objetivos foram: identificar publicações científicas dos últimos 10 anos, nacionais e internacionais, sobre o conhecimento de médicos e enfermeiros no atendimento ao portador de demência e seus familiares e apresentar um panorama desse conhecimento. A consulta foi realizada em fevereiro de 2013 na base de dados eletrônica MEDLINE, usando os descritores: “Knowledge”, “Health Personnel”, “Dementia”. O levantamento bibliográfico identificou 523 publicações e, destas, foram selecionadas 13 por atenderem aos critérios de inclusão no estudo. Os achados revelaram predomínio de estudos de delineamento qualitativo publicados no ano de 2010, desenvolvidos em diversas áreas do conhecimento, com foco especial para Enfermagem e Geriatria. Os resultados evidenciaram déficit de conhecimento de médicos e enfermeiros, tanto em relação ao diagnóstico, como de acompanhamento/suporte ao portador de demência e seus familiares. No entanto, verificou-se que quando se tratava de médicos e enfermeiros especialistas na área, estes demonstraram conhecimento em relação à fisiopatologia e tratamento farmacológico, mas referiram falta de autonomia e treinamentos específicos sobre o tema. Conclui-se que muito precisa ser feito para melhorar o processo de trabalho desses profissionais. Sugere-se, em primeiro momento, programas de capacitação para a melhoria de suas práticas, o que será um ganho para os pacientes portadores de demência e seus familiares.

**OFICINA SAÚDE NO ENVELHECER: UM RELATO DE EXPERIÊNCIA** *Eminy Winer Araujo; Maria Margarida de Vasconcelos Oliveira*

**RESUMO.** O aumento da população idosa e da expectativa de vida em nível nacional e local, reflexo das transformações demográficas ocorridas nas últimas décadas, suscita o planejamento e implementação de ações que objetivem a inclusão social e uma melhor qualidade de vida para esta população. Com base em tais considerações, realizamos a oficina “Saúde no Envelhecer”, através da Universidade Aberta à Terceira Idade (UATI), programa de extensão da Universidade Estadual de Feira de Santana, buscando promover o desenvolvimento de práticas educativas e a melhoria do estilo de vida e das relações interpessoais dos idosos, bem como favorecer as adaptações aos problemas da vida diária. Neste trabalho visamos relatar a experiência vivenciada com a execução de tal oficina. Foram realizados encontros semanais durante o período de fevereiro a dezembro de 2011, junto a um grupo de 17 alunos da UATI com faixa etária de 50 a 80 anos, destes 16 foram do sexo feminino e 02 do sexo masculino. As atividades realizadas na oficina possibilitaram maior integração do grupo, valorização da própria imagem, da identidade e do ser, estímulo para leitura e escrita, elevação da autoestima, a reflexão de ações para prevenção de agravos à saúde, contribuindo para promoção de uma longevidade saudável. Com a aplicação da Avaliação Multidimensional do Idoso, assim como o desenvolvimento de atividades como leitura e reflexão de textos e discussão de temas, permitiram identificar nos participantes alguns aspectos físicos, psíquicos, sociais e espirituais, assim como adaptações cognitivas e emocionais. Ressaltamos também que a execução de jogos e atividades, como quebra-cabeça e caça-palavras, estimula a memorização, atenção e raciocínio lógico dos participantes e que os trabalhos manuais e artesanais também permitem o desenvolvimento cognitivo e intelectual e a autoexpressão dos alunos e a prática de alguns exercícios estimula a manutenção do equilíbrio. Concluímos que as ações desenvolvidas com a realização da oficina e dos demais projetos e eventos da UATI, além de serem positivas para a formação acadêmica dos facilitadores, permitem a valorização e crescimento pessoal do idoso e dos adultos que se encontram rumo à velhice, favorecendo a valorização da identidade, o resgate cultural, melhor aceitação social destes e melhoria da qualidade de vida. Palavras-chave: educação em saúde, promoção da saúde, longevidade.

**OLDER WOMEN WIDOWS IN CONTEXT OF SOCIAL INTERACTION: EVALUATION OF DEPRESSIVE SYMPTOMS AND COGNITION** *Allan Gustavo Brigola; Vivian Ramos Melhado*

**ABSTRACT.** The phenomenon of aging population is observed worldwide and projections are announced some years ago by several researchers. Another phenomenon is associated with this process called Feminization of old age, which refers to the high presence of older women in population as well as the greater longevity of women compared with men. Consequently, they have more probability to experience such events in life as death of a spouse or partner. The purpose of this research was to investigate the association between husband‘s death, cognitive performance and depressive symptoms in old women. The research design is cross-sectional, quantitative character and descriptive-correlational. The criteria for choosing the target public were being 60 years and over, participating in an Universidade Aberta da Terceira Idade of an inner city in São Paulo, Brazil, whose partner‘s death occurred in less than 30 months. It was applied to the data collection the evaluation of depressive symptoms instrument Geriatric Depression Scale - version 30 - and Mini-Mental State Examination to the cognitive evaluation, besides a questionnaire about subjects. The data analyzes were performed using scatter plots, Spearman correlation coefficient and the Mann-Whitney test. This research was approved by Comitê de Ética/UFSCar. Characterizing the data's presentation, were interviewed seven old women widows with avareges: age=70.5 years; education=4.12 years, duration of marriage=48.8 years; widowhood time=17.7 months, frequency social interaction time=54.8 months; GDS score=6.8; and MMSE scores=24.7. By the results, none of the women had cognitive deficits or cognitive loss, showing only depressive symptoms. The closest relationships were education-MMSE scores and widowhood time-GDS score. They are discussed the benefits of social interaction to cognition and the association with the depressive symptoms in a sample of widows. Frequenting social environments before the loss of husband may be a protective factor for major depressive symptoms and poor cognitive performance. It is suggested that professionals are prepared to meet the demands of the widowed elderly and may be offered guidelines for training about finitude and death. The Bachelor in Gerontology is a model professional trained to understand and intervene in impacts caused by aging.

**OPERATIVE GROUP WITH INSTITUTIONALIZED ELDERLY CAREGIVERS: A LIGHT ON ORTHOSTATIC HYPOTENSION** *Pedro Henrique Ferreira Guimarães; Danilo Ferreira Maia; Almir Ribeiro Tavares Junior*

**ABSTRACT.** Orthostatic hypotension (OH) is clinically diagnosed as a reduction in systolic blood pressure of at least 20 mmHg or a reduction in diastolic blood pressure of at least 10 mmHg and it is measured within the first three minutes of standing. When symptomatic OH can cause syncope, presyncope, dizziness, angina or light-headedness in response to postural change, although asymptomatic or nonspecific clinical symptoms such as generalized weakness, fatigue and cognitive slowing are less easily recognized. The prevalence increases with age and institutionalization in long-term care facility (LTCF), reaching 60% in some series. The present report aims to describe the experience of an operative group composed of caregivers and medical students towards simple measures for HO prevention. Group-centered learning activities using Pitchon-Riviere's approach were performed in one LTCF in Belo Horizonte with at least three caregivers. Medical students offered a brief explanation about HO and subsequently caregivers were able to identify institutionalized patients with possible diagnosis of HO. During discussions, caregivers proposed non-pharmacological therapies and preventive physical maneuvers based on their experience such as arising slowing and counseling patients to maintain hydration. Others interventions were informed by the coordinator of the group, such as using of elastic stockings in lower extremities and physical activities. As institutionalized patients are under higher risk of orthostatic hypotension, clarification of the problem for caregivers is an important matter. Therefore we propose a simple method for which caregivers can recognize the interventions within their reach.

**OVERLOAD OF FAMILY CAREGIVER OF ELDERLY BY COGNITIVE DEVELOPMENT IN THE CITY OF RECIFE** *Jéssika Melo Leão Bezerra; Anna Carolina de Castro Araújo Lessa; Apoenna Mirelly de Lima Azevedo; Celivane Cavalcanti Barbosa; Alexandre de Mattos Gomes; Edvan Lima; Fábia Maria Lima*

**ABSTRACT.** **Objective:** To identify the overload of family caregivers of elderly according to cognitive performance in the city of Recife. **Methodology:** This is a cross-sectional study, observational with descriptive character. Sample was made up of 59 elderly patients with primary family caregiver of health district VI in Recife. The following instruments were used for characterization of the elderly: sociodemographic data, Mini Mental State Examination (MMSE) scale and Katz; for the characterization of the caregivers were used sociodemographic data and overload scale of Zarit. This study was approved by the Research Ethics committee of HUOC / Procape by CAAE n. 04286312.9.0000.5192. **Results:** In the present study were included a total of 59 elderly, 50 with being changed MMSE and 9 with Normal MMSE. Generally speaking, in the elderly there was a predominance of the age group of 70-79 (41%), female (76%), illiterates (41%), widowers (46%). In Katz, 86% were independent elderly. As for caregivers the prevalence was: age group 50-59 (27%), female (66%), 5-8 years of schooling (36%), single (44%), degree of relationship -child (69%). There were in caregivers of elderly with cognitive impairment, severe overload (4%). **Conclusion:** it was found that dependence on activities of daily living and cognitive deficit of the elderly cause the overload of the caregiver. Key words: Elderly; caregivers; family; overload.

**PERFIL FUNCIONAL DOS IDOSOS ATENDIDOS EM UM AMBULATÓRIO DE REFERÊNCIA EM GERIATRIA** *Cristiana Ceotto Deslandes ; Flávia Lanna de Moraes; Avelina Cristina Conceição; Edgar Nunes de Moraes*

**RESUMO. Introdução:** O rápido envelhecimento da população brasileira representa um grande desafio para o Sistema Único de Saúde, devido ao aparecimento de novas demandas associadas às doenças crônico-degenerativas e às incapacidades. A dependência funcional é frequente na população idosa e sua abordagem exige um remodelamento dos programas de atenção à saúde, tanto públicos quanto privados. **Objetivo:** Avaliar o perfil funcional dos idosos residentes na Regional Nordeste de Belo Horizonte, atendidos em ambulatório especializado em Geriatria e Gerontologia, no período de novembro de 2012 a maio de 2013. **Metodologia:** Foram coletados dados obtidos de prontuários dos pacientes residentes na região nordeste de Belo Horizonte, atendidos pelo serviço de Geriatria do Hospital das Clínicas da UFMG, no período de novembro de 2012 a maio de 2013. **Resultados:** Foram avaliados 124 prontuários. A média de idade foi de 78,5 anos. Houve predomínio do sexo feminino, com 74,2% dos pacientes. A escolaridade média foi de 3,9 anos. A dependência nas atividades de vida diária instrumentais (AVDI) foi de 65,3%, com dependência parcial e total em, respectivamente, 34% e 31,3%. Por sua vez, a prevalência de dependência nas atividades de vida diária básicas foi de 30,5%, com 5% dos idosos apresentando dependência completa. **Conclusão:** A população idosa atendida em ambulatório de referência de Belo Horizonte apresentou alta prevalência de declínio funcional, tanto nas AVD's instrumentais quanto nas básicas. O serviço é voltado para o atendimento de idosos frágeis e os critérios de encaminhamento são rigorosos. Percebe-se, assim, que os encaminhamentos realizados pela atenção básica são adequados, permitindo uma atenção diferenciada ao idoso mais frágil.

**POLIFARMÁCIA EM IDOSOS COM DEMÊNCIA AVANÇADA RESIDENTES EM UMA ILPI** *Renata Firpo R. Medeiros; Vania Ferreira de Sá Mayoral; Iracelia Munhoz Moreira; Mercia Gomes Rodrigues; Gisele Monaco Dias; Ana Lúcia Alves; Audrey Andrade Bertoloni; Geovanna Adriana Ayala da Silva*

**RESUMO. Introdução:** A prevalência de doenças crônicas tem contribuido para o aumento da utilização de medicamentos nas últimas décadas pelos idosos. A polifarmácia é definida como o uso de cinco ou mais medicamentos. Nos idosos com doenças neurodegenerativas avançadas a meta dos cuidados deve orientar a prática de prescrição de medicamentos. A polifarmácia gera aumento no risco de reações adversas aos medicamentos (RAM), sobrecarrega os custos de manutenção de tratamento, tanto nas Instituições quanto do ponto de vista individual e familiar. **Objetivo:** Identificar o número de medicamentos utilizados em idosos com demência avançada residentes em uma ILPi. **Metodologia:** Estudo de corte transversal para avaliar o número de medicamentos em moradores de uma ILP. Foi realizada a revisão de prontuários para coleta de dados epidemiológicos, diagnósticos e número de medicamentos de uso contínuo nos últimos 3 meses. Foram aplicados a escala Clinical Dementia Rating (CDR) e pelo Functional Assesment Staging (FAST), Índice de comorbidades de Charlson (ICC) e Mini Exame do Estado Mental (MEEM). **Resultados:** Foram avaliados 42 moradores com idade média de 86,31±8,30 anos, sendo 92,68% do sexo feminino e 7,32 do sexo masculino. No CDR 61,90% da amostra foi caracterizada com demência grave e 14,28% com demência moderada, com um CDR médio de 2,02±1,09. No estadiamento funcional (FAST), 66,66% dos indivíduos alcançaram escores que determinaram demência grave e 28,57% como demência moderada-grave. O ICC médio foi 1,5±0,9. A amostra obteve um MEEM de 8,14±9,83. O número médio de medicamentos administrados diariamente foi de 8,19±2,91 dia/residente. Residentes que usavam 5 ou mais medicamentos por dia foram 87,81%. Os antihipertensivos, antipsicóticos, protetores gástricos, procinéticos e broncodilatadores, foram os medicamentos mais prescritos. **Conclusão:** A prescrição de medicamentos é um fenômeno complexo que deve priorizar esforços para melhorar a qualidade dos cuidados farmacológicos em pacientes vulneráveis aos eventos adversos. Idosos com demência apresentam também multicomorbidades. Questionamento do benefício de medicamentos são comuns em demência avançada principalmente quando a morte se aproxima. O uso de medicamento em demência avançada deve ser adaptado para as metas de atendimento.

**POTENTIALLY INAPPROPRIATE MEDICATIONS BY THE CRITERIA OF BEERS, USED FOR ELDERS OF THE CITY OF RECIFE - PROJECT PERNAMBUCO** *Tatiana Cristina Nascimento Ramos de Souza; Fábia Maria de Lima; Marília Siqueira Campos; Alexandra Siqueira Campos; Bruna Alves Bispo Jardim; Laura Carolina Carvalho Fernandes; Shyrleide Daniella Cunha Bezerra; Yohana Veras de Oliveira; Thamires Tavares*

**ABSTRACT.** In Brazil, the elderly represent 8.6% of the population. Featuring about 20 million seniors. By 2025, that number should rise to 32 million people. Due to the increasing number of these individuals, the consumption of drugs by this population follows this trend. It is common to find in their prescriptions and dosages inappropriate indications, drug interactions, associations, and drugs without therapeutic value. These factors may cause adverse drug reactions, some severe and deadly. **Objectives:** This study aims to identify potentially inappropriate medications, according to the Beers criteria, the 2003 version, among the elderly in the city of Recife-PE. **Methodology:** We conducted a population-based study and cross-section, from database design Pernambuco held in the year 2012/2013. The study site was in the city of Recife in Pernambuco, with a sample of 67 individuals who participated in the analysis. The variables studied were: sex, age, marital status, schooling, those who live, income, physical exercise, smoking, alcohol, previous diseases and medications most prevalent. Drugs were classified according to the Beers Criteria. This study was approved by the Ethics Committee on Human Research of the University Hospital Oswaldo Cruz by and filed under number 04273412.9.0000.5192. **Results:** t was found that 27% of elderly study used potentially inappropriate medications (MPI) according to the Beers Criteria 2012 version. The age group ranged from 50 to 90 years of age, with a prevalence of 46% seniors 61-70 years. Women accounted for 78% of the sample. There was no significant difference between the means of those who use and those who do not use MPI. MPI nine were found in total (28%), among these, the most widely used was the AAS, totaling 21%. In the age group 50-60 years, the prevalence of MPI was 1.7%, significantly lower than the prevalence of 46% observed among seniors aged 61 to 70 years. The prevalence of the use of MPI among men was 40% higher than the reported 32% prevalence among women. **Conclusion:** There is a pattern of high drug use among people aged less than 60 years living in the city of Recife. The challenge for health professionals is to promote alternative measures to the overuse of drugs by the elderly population through dietary change, the physical exercise and social inclusion of the elderly, attitudes that can contribute so effectively on the use of drug.

**PREVALÊNCIA DE COMORBIDADES MÚLTIPLAS NOS IDOSOS ATENDIDOS PELA GERIATRIA EM VISITA DOMICILIAR** *Cristiana Ceotto Deslandes; Clarissa De Angelis Vieira dos Santos; Fabiana Silveira Duarte*

**RESUMO. Introdução:** Os idosos altamente dependentes, restritos ao leito ou ao lar, apresentam alta prevalência de declínio funcional e comorbidades múltiplas. O conceito adotado de comorbidades múltiplas inclui a presença de polipatologia ou polifarmácia ou história de internações recentes e/ou pós-alta hospitalar. O manejo clínico desses pacientes é complexo e exige a presença de uma equipe geriátrico-gerontológica especializada, com disponibilidade para atendimento domiciliar pela dificuldade de locomoção do paciente. O Programa Mais Vida em Casa (PMVC) tem o objetivo de avaliar e monitorar os idosos restritos leito ou ao lar, residentes na regional Nordeste de Belo Horizonte, em parceria com a equipe de saúde da família. **Objetivo:** Avaliar a prevalência de comorbidade múltiplas nos idosos atendidos pelo PMVC, bem como aplicação de indice de comorbidade de Charlson. **Métodos:** Estudo transversal abrangendo uma amostra de 555 pacientes atendidos no período 06/01/2011 a 09/05/13. A análise estatística foi realizada pelos pacotes estatísticos SPSS 17.0. **Resultados:** Os 555 pacientes apresentam média de idade de 81,33 (DP 9,48) anos, escolaridade de 2,8 (DP 2,66) anos e 67% do sexo feminino. A circunferência de panturrilha média foi de 29,93 cm (DP 5,16) e o número de medicamentos prescritos por dia foi de 5 (DP 2,55). Cerca de 27% dos pacientes apresentaram pelo menos uma internação após a visita do PMVC. O número médio de comorbidades foi de 3 diagnósticos por paciente. O índice de comorbidades de Charlson (ICC) médio foi de 6,04 (DP 2,08). Dentre as condições crônicas de saúde mais prevalentes, destacam-se: demência (62,5%), depressão (45,9%), dor crônica (38,6%), AVC/ AIT (33,2%), diabetes mellitus (21,8%) e insuficiência cardíaca (10,3%). Entre as demências, a mais prevalente é a doença de Alzheimer (46,7%), seguido da demência vascular (22%) e mista (19%). **Conclusão:** A prevalência comorbidades múltiplas foi frequente na população estudada. As doenças crônico-degenerativas foram altamente prevalentes, principalmente as síndromes demenciais, e, em particular, a doença de Alzheimer. Tais doenças estão associadas a grande comprometimento funcional e, consequentemente, restrição ao leito ou ao lar.

**PREVALÊNCIA DE COMORBIDADES MÚLTIPLAS NOS IDOSOS ATENDIDOS PELA GERIATRIA EM VISITA DOMICILIAR** *Cristiana Ceotto Deslandes; Clarissa De Angelis Vieira dos Santos; Fabiana Silveira Duarte; Erika de Oliveira Hansen; Flávia Lanna de Moraes; Edgar Nunes de Moraes*

**RESUMO. Introdução:** Os idosos altamente dependentes, restritos ao leito ou ao lar, apresentam alta prevalência de declínio funcional e comorbidades múltiplas. O conceito adotado de comorbidades múltiplas inclui a presença de polipatologia ou polifarmácia ou história de internações recentes e/ou pós-alta hospitalar. O manejo clínico desses pacientes é complexo e exige a presença de uma equipe geriátrico-gerontológica especializada, com disponibilidade para atendimento domiciliar pela dificuldade de locomoção do paciente. O Programa Mais Vida em Casa (PMVC) tem o objetivo de avaliar e monitorar os idosos restritos leito ou ao lar, residentes na regional Nordeste de Belo Horizonte, em parceria com a equipe de saúde da família. **Objetivo:** Avaliar a prevalência de comorbidade múltiplas nos idosos atendidos pelo PMVC, bem como aplicação de indice de comorbidade de Charlson. **Métodos:** Estudo transversal abrangendo uma amostra de 555 pacientes atendidos no período 06/01/2011 a 09/05/13. A análise estatística foi realizada pelos pacotes estatísticos SPSS 17.0. **Resultados:** Os 555 pacientes apresentam média de idade de 81,33 (DP 9,48) anos, escolaridade de 2,8 (DP 2,66) anos e 67% do sexo feminino. A circunferência de panturrilha média foi de 29,93 cm (DP 5,16) e o número de medicamentos prescritos por dia foi de 5 (DP 2,55). Cerca de 27% dos pacientes apresentaram pelo menos uma internação após a visita do PMVC. O número médio de comorbidades foi de 3 diagnósticos por paciente. O índice de comorbidades de Charlson (ICC) médio foi de 6,04 (DP 2,08). Dentre as condições crônicas de saúde mais prevalentes, destacam-se: demência (62,5%), depressão (45,9%), dor crônica (38,6%), AVC/ AIT (33,2%), diabetes mellitus (21,8%) e insuficiência cardíaca (10,3%). Entre as demências, a mais prevalente é a doença de Alzheimer (46,7%), seguido da demência vascular (22%) e mista (19%). **Conclusão:** A prevalência comorbidades múltiplas foi frequente na população estudada. As doenças crônico-degenerativas foram altamente prevalentes, principalmente as síndromes demenciais, e, em particular, a doença de Alzheimer. Tais doenças estão associadas a grande comprometimento funcional e, consequentemente, restrição ao leito ou ao lar.

**PREVALÊNCIA DE VITAMINA B12 NOS IDOSOS ATENDIDOS PELO CENTRO MAIS VIDA DE BELO HORIZONTE/MG** *Leonardo Vinícius de Andrade; Gabriella Polastri Stiilpen; Clarissa De Angelis Vieira dos Santos; Sandra Cristina Maciel de Lacerda; Deborah Mendonça Lima; Marcus Vinícius Tostes Ferreira; Marco Túlio Gualberto Cintra; Maria aparecida Camargos Bicalho*

**RESUMO. Introdução:** A deficiência de vitamina B12 ocorre com grande freqüência (10 a 24 por cento) em idosos. Geralmente apresenta sintomatologia sutil relacionados a distúrbios neurológicos, enquanto as alterações hematológicas manifestam-se tardiamente. É considerada uma causa de demência potencialmente reversível. **Objetivo:** Avaliar a prevalência da deficiência de B12 nos idosos avaliados no Centro Mais Vida de Belo Horizonte/ MG, bem como analisar a associação com demência. **Métodos:** Estudo transversal com 455 idosos atendidos no Centro Mais Vida de Belo Horizonte/MG no período de janeiro de 2011 a dezembro de 2012. Optou-se por dicotomizar a amostra e considerar deficiência de vitamina B12 níveis séricos abaixo de 350pg/ml, porque este ponto de corte tem sensibilidade de 90% de detectar a deficiência quando comparada à dosagem de ácido metilmalônico. Foram utilizados os testes Qui-quadrado e Fisher para variáveis categóricas e os testes t e Mann-Whitney para variáveis contínuas, além de técnica de regressão logística para análise multivariada. A análise estatística foi realizada pelo pacote estatístico SPSS 19.0. **Resultados:** A amostra é constituída de 73,8% de mulheres, média de idade de 76,57±8,47 anos e escolaridade de 3,82±3,07 anos. Dos 455 pacientes avaliados, 177 apresentaram baixos níveis da vitamina (38,9% da amostra). Constatou-se que

o sexo masculino está associado com a deficiência de B12 (OR: 1,62 e IC 95%: 1,04- 2,50; p=0,023). A deficiência de vitamina B12 é fator de risco para demência (OR: 1,72 e IC 95% 1,14- 2,59; p=0,006). As variáveis: idade (limiar estatístico; p=0,054), escolaridade, clearance de creatinina, IMC e circunferência de panturrilha não apresentaram associação com deficiência de vitamina B12. Na análise multivariada observou-se associação da deficiência de B12 com demência (p=0,004) e com dependência em AVD's básicas (p=0,020). **Conclusão:** Níveis baixos de B12 foram encontrados em um percentual significativo de nossa amostra e associados em análise multivariada com demência. A deficiência de B12 é uma das causas potencialmente reversível de demência, devendo ser avaliada todos os casos de suspeita de demência. Ressalta-se que mesmo em pacientes com quadros degenerativos de demência se pode observar déficits de vitamina B12 que podem contribuir para a sintomatologia cognitiva. O tratamento é eficaz e está disponível gratuitamente pelo Sistema Único de Saúde.

**PREVENTION OF COGNITIVE IMPAIRMENT THROUGH A COGNITIVE STIMULATION AND REHABILITATION PROGRAM MEDIATED BY COMPUTERS AND INTERNET, A INTERVENTION QUASI EXPERIMENTAL STUDY** *André Junqueira Xavier; Anderson Matte Ifontana; Eduardo Rosa De Oliveira; Layo Nikson Oliveira Lima Queiroz; Vinicius Carriero Lima; Vinicius Cenci Guarienti*

**ABSTRACT.** Background: Neurodegenerative diseases present high prevalence over 50 years and geometrical progression over 60 years. There is evidence that Mild Cognitive Impairment can be previous steps towards dementia and are also preventable by Cognitive Stimulation and Rehabilitation (CSR). “Remembrance Workshop” is a CSR program based on computers and internet use. **Objective:** To determine whether a CSR program based in computers and internet use can prevent cognitive impairment measured by MMSE. Material and **Methods:** Intervention controlled cohort study, quasi experimental, approved by Brazilian National Health Council. All participants were independent and autonomous with 60 or more years, community dwelling, had memory complaints and non demented. Analysis performed by logistic regression after univariate and bivariate analysis, p<

0.05. Outcome was MMSE<25 higher schholing and MMSE<20 low schooling, Veras (2006) after the second interview. Intervention (CSR): 20 biweekly workshops, 1.5 hours each, based in computers and internet use in computer laboratories (Remembrance Workshp). Intervention group was CRS program plus medical follow-up and control group was only medical follow-up. **Results:** Population 212, age 69,13±7,28, MMSE 27,85±1,79, female 173(86%). Intervention group 113, 109 (96,5) remained without cognitive impairment and 4 (3,5%) showed cognitive worsening. Control group, 99, 91 (80.0%) without cognitive impairment and 8 (20,0%) with cognitive worsening. Control variables: gender, age, education, marital status, social status, physical activity, obesity, depression, cardiovascular disease, cerebrovascular disease, hypothyroidism, polypharmacy, dyslipidemia, smoking, use of benzodiazepines, diabetes, functional capacity, time between the first and second interview, initial MMSE. In the final model, CSR was a significant protective factor of 4,67 (1,05-20,62 95%CI) p=0,042 together with study years protective factor of 1,21 (1,05-1,46 95%CI) p=0,034, NNT=22,2. **Conclusion:** Remembrance Workshop CRS program and educational level were was associated independently with prevention of cognitive impairment among older persons with memory complaints.

**QUEDAS E OS IMPACTOS PSICOSSOCIAIS NA VIDA DO IDOSO** *Ana Elizabeth dos Santos Lins; Séris Darlley Santos da Silva; Gracinda Maria Gomes Alves; Francelise Pivetta Roque*

**RESUMO. Introdução:** O envelhecimento é um processo de desenvolvimento normal, no qual a população idosa tem aumentado consideravelmente nos últimos anos. Uma boa qualidade de vida é almejada por todos e com o idoso não é diferente. Durante esse processo o idoso fica vulnerável a uma série de eventos, a queda é um deles, tornando-se um fator de risco para manutenção de uma boa qualidade de vida. **Método:** Estudo transversal. A amostra foi de 30 idosos, divididos em dois grupos de 15 idosos, um que sofreu quedas e o grupo que não sofreu. Utilizou-se o instrumento (SF-36) para medir qualidade de vida (QV) e o grupo que sofreu quedas respondeu um questionário sobre quedas. A pesquisa foi realizada no mês de outubro de 2012 com usuários da Unidade Básica de Saúde Dr. Hélvio Alto no município de Maceió-Alagoas. Para análise dos dados foi utilizado o software BioEstat 5.0 **Resultados:** A prevalência de quedas foi no sexo feminino (73%), a maioria casada (53%); apenas 40% fazia algum tipo de atividade física; 86% usava medicamentos prescritos por médicos, e 73% apresentaram alguma dificuldade de mobilidade. O questionário de QV (SF-36), o domínio capacidade funcional a média para o grupo que sofreu queda foi de 53.3, já para o outro grupo foi 72.3. A limitação por aspectos físicos, o grupo que sofreu quedas apresentou média igual a 30. O domínio dor, o grupo de idosos que sofreu quedas, a média foi de 55.2, do domínio do estado geral da saúde foi 54.7. Outros domínios estudados como vitalidade, aspectos sociais, limitação por aspectos emocionais e saúde mental,

o grupo que sofreu quedas continuou apresentando média inferior ao grupo que não sofreu quedas, sendo as médias 48.6, 47.3,

49.6 e 48.6. Constatou-se que houve uma diferença significante na média dos grupos, onde 100% dos domínios foram menores para o grupo que sofreu quedas. A queda teve maior incidência na própria residência e no banheiro. **Conclusão:** Observamos que

o evento quedas prejudica a vida do idoso, necessitando da intervenção da equipe de saúde, incluindo a Terapia Ocupacional com ações preventivas e de reabilitação, avaliação e adaptações no ambiente para ajudar no melhor desempenho nas Atividades de Vida Diária (AVDs), fazendo com que o idoso continue independente, autônomo e possa desfrutar de melhor qualidade de vida.

**RECOGNIZING BURDEN AND QUALITY OF LIFE OF FRAIL ELDERLY FAMILY CAREGIVERS** *Patrick Alexander Wachholz; Rosa Cristina Cervi Santos; Loreci Santos Pereira Wolf*

**ABSTRACT.** Tasks assigned to family caregivers may add strong physical and mental burden; these activities are often performed without proper orientation, without the support of other family members, and might change the whole routine of their previous life, occupying most of the day. Literature suggests that these detrimental effects of care could be primarily associated with patients' functional disabilities, cognitive or behavioral impairment, and by the intensity demand for care. However, caregivers are also affected by emotional experiences and psychological distress, and the effects of these interrelationships in the quality of life of caregivers are only partly known. **Objective:** This study aimed to analyze the correlation between the levels of functional dependence of elderly residents in the community, the burden related to care and the perception of quality of life in familiar caregivers. **Methods:** This is an observational, descriptive and analytical study, using non probabilistic sampling selected by convenience in the period from 12/2008 to 05/2009, from the urban area of Curitiba and Colombo-PR. Interviews were applied in the caregivers, using demographic inquiry, functional evaluation of the aged, burden interview (Zarit-Burden-Interview) and quality of life instrument (WHOQOL-Bref). Spearman, Mann-Whitney and Kruskal Wallis coefficients were used to analyze the correlations between the instruments and the socio-demographic variables. Bivariate analyses identified which variables correlate with burden, the most significant were included in a multiple linear regression. **Results:** Forty-five caregivers had been interviewed, with predominance of women (91.11%) with elevated scholarship attending dependent aged (66.77%). Moderate/severe burden was perceived in 75.55% of the sample. We found correlation between dependence, more severe burden in the caregiver (r= -0.281, p=0.013) and worse perception of quality of life. The multiple linear regression identified a strong association between burden related to care and psychological domain from WHO-QOL-bref and time as caregiver (R2=0.58,p<0.001). **Conclusion:** In a sample of familiar caregivers, we identified correlations between lower burden related to care and better quality of life perceptions, as well as higher disability and less satisfactory quality of life perceptions.

**RELATO DE CASO- LUPUS EM IDOSO: ABERTURA DO QUADRO CLÍNICO COM ALTERAÇÃO COGNITIVO-COMPORTAMENTAL** *Lidiane Cristina Nitshe; Cecília K. R. Feder; Stephanie F. Levy; Renato M. A. Fabbri; Milton Luiz Gorzoni; Sueli Luciano Pires; Camila B. Guanais; Andrea C. Brito*

**RESUMO. Introdução:** O Lúpus Eritematoso Sistêmico (LES) é uma enfermidade multissistêmica, de etiologia considerada idiopática, na qual células e tecidos são danificados por anticorpos patogênicos e complexos antígeno-anticorpos, cuja epidemiologia é caracterizada por acometimento feminino em idade reprodutiva, mas também pessoas a partir da 6ª década de vida. A principal dificuldade de se fazer um diagnóstico de LES no Idoso se dá ao quadro clinico mais frustro, sem tanta sintomatologia. Um fator complicador é a manifestação atípica da doença como perda de peso, alteração cognitiva ou dores musculares. **Objetivo:** relatar o caso de homem idoso que deu entrada no pronto socorro por alteração aguda cognitivo-comportamental cujo diagnóstico clínico foi de lúpus eritematoso sistêmico. **Método:** revisão de prontuário confrontando história clínica com exames laboratoriais e revisão da literatura. **Resultados:** Relato de caso de homem de 60 anos que deu entrada no pronto socorro por alteração cognitivo-comportamental sem outras queixas.Observou-se alteração hematológica com esplenomegalia e pancitopenia (Hemoglobina:9,3; leucócitos:2490; plaquetas:100000) e inicialmente pensou-se em processo neoplásio, todavia houve evolução para insuficiência renal (Cr:6,8; proteinúria 3g/dia; biópsia renal acusou glomerulonefrite lupica tipo IV), distúrbio imunológico (Anti-SM:55,7; Anti-DNA: 1/160), FAN nuclear positivo 1/640 e por fim serosite pleural. Sendo assim, diante de mais de 4 critérios para Lupus positivos, fez-se o diagnóstico de LES. **Conclusão:** O LES em Idoso representa de 10% a 20% da população com LES. Seu diagnóstico é freqüentemente mais demorado devido a suas manifestações clínicas serem insidiosas e mais brandas do que no jovem, mas nem por isso mais inocente, sendo observadas principalmente alterações oculares, musculoesqueléticas e cardiovasculares.

**RODA DE CONVERSA: A INSERÇÃO DO IDOSO NO *COTIDIANO*** *Regina Celia Marques de Mello; Maria Tércia Barroso Pereira Malta; Maria Lucia Carneiro dos Rios Ferreira; Marta Cristina Ayres Neves Porto; Helayne da Costa Coelho*

**RESUMO. Introdução:** O envelhecimento populacional tornou-se um fenômeno mundial. Já não é mais privilégio de poucas pessoas poder chegar a uma idade avançada. No Brasil, na última década, houve um aumento de 3 anos na expectativa de vida. Entretanto, os idosos passaram a ser vistos como vítimas da solidão e de uma sociedade que os marginaliza. Diversas pesquisas demonstram haver uma correlação negativa entre participação em atividades sociais e depressão; e isso reforça a importância de atividades e a inserção de idosos em grupos sociais. As grandes modificações por que passa a sociedade, as novas tecnologias e o excesso de informações, exige uma capacidade de adaptação que nem sempre o idoso possui, acarretando afastamento do convívio social. Baseado nessa realidade, foi implantada no Grupo Renascer, Programa desenvolvido no Hospital Universitário Gaffrée e Guinle/UNIRIO, a Roda de Conversa: conversação grupal em que os assuntos debatidos são escolhidos livremente pelos participantes, para que os idosos possam opinar sobre assuntos veiculados pelos meios de comunicação. **Objetivo:** Proporcionar aos idosos um espaço de discussão de temas atuais, como forma de inserção no cotidiano; proporcionar aos idosos espaço para falar sobre sua própria vida a partir dos temas propostos oferecendo um momento de sociabilidade e integração. **Método:** A Roda de Conversa ocorre uma vez por semana, moderada por uma psicóloga da equipe e com uma hora de duração. Não há limite de vagas e a freqüência é livre.O tema discutido é escolhido pelos idosos, sendo todos incentivados a dar sua opinião. Nas Rodas de Conversa os participantes são incentivados a discutir sobre um tema atual, divulgado pela mídia e expressando-se livremente, e ao escutar a si e aos outros, também estão estimulando sua memória. **Resultados:** A cada semana observa-se um aumento da freqüência na Roda de Conversa. Esse resultado demonstra a importância de se dar voz ao idoso, de proporcionar a ele a oportunidade de posicionamento frente ao que ocorre no mundo. **Conclusão:** A possibilidade de raciocinar, exercita sua fala, sua escuta e sua leitura em relação aos acontecimentos, exerce uma melhora na autoestima. O isolamento sentido na família e na sociedade de uma maneira geral, é minimizado pelo sentimento que têm ao ver que suas opiniões são aceitas e respeitadas. Observa-se o desejo de saber, de informar-se e colocar suas opiniões no grupo. Participar da Roda de Conversa permite que se sintam amparados e acolhidos.

**SIGNIFICADO DA CONVIVÊNCIA GRUPAL PARA O PROCESSO DE ENVELHECER NA PERCEPÇÃO DO IDOSO** *Eminy Winer Araujo; Maria Margarida de Vasconcelos Oliveira*

**RESUMO.** Uma das modificações demográficas mais significativas das últimas décadas do século XX em nível nacional e local tem se constituído em um rápido e acentuado crescimento da população idosa e da expectativa de vida, resultado da diminuição dos níveis de fecundidade e de mortalidade. Desta forma, impõe-se a necessidade de ampliar a consciência sobre o envelhecer e os recursos para manutenção da saúde do idoso, ao mesmo tempo fortalecendo e instrumentalizando a população em suas lutas por cidadania e justiça social. Nesse sentido, surgem as universidades abertas à terceira idade e os chamados Centros de Terceira Idade ou grupos de convivência. Neste estudo qualitativo, de caráter exploratório, buscamos compreender o significado da convivência grupal para os idosos do grupo de Convivência Esperança e identificar a influência da convivência grupal no processo de envelhecer dos idosos. Para coleta dos dados foram realizadas entrevistas por meio de três sessões de grupo focal com 20 idosos do grupo de convivência Esperança do Distrito de Maria Quitéria vinculado à Universidade Aberta à Terceira Idade (UATI), programa de extensão da Universidade Estadual de Feira de Santana, em Feira de Santana, Bahia, sendo dezoito do sexo feminino e dois do sexo masculino, com faixa etária entre 60 e 89 anos. Para análise dos dados utilizamos o método da análise de conteúdo. Após a leitura criteriosa dos dados foram apreendidas as categorias a seguir: significado de convivência grupal na percepção do idoso e a influência da convivência em grupo no processo de envelhecimento. Os resultados mostraram que a convivência grupal para os idosos consiste em fonte de conhecimento e aprendizagem, espaço de cultura e lazer e estabelecimento de vínculos sociais e afetivos e que o processo de envelhecimento pode ser influenciado positivamente pela convivência grupal, na medida em que esta se relaciona diretamente com a promoção do envelhecimento bem-sucedido, ressignificação da velhice para o idoso e sua família e sentido de vida. Consideramos, portanto, que a integração de idosos através da convivência grupal pode gerar inúmeros benefícios, na medida em pode e deve ser espaço de promoção de autonomia, independência e ajuda mútua, de educação em saúde, de ações terapêuticas e outras ações que possibilitem uma melhoria da qualidade de vida dos participantes. Palavras-chave: convivência grupal, envelhecimento, idosos

**STRESS LEVEL OF CAREGIVERS FOR THE ELDERLY IN A LONG-STAY INSTITUTION - ILPI** *Rosane Paula Nierotka; Samila Livinalli; Camila Malesza; Marilene Rodrigues Portella*

**ABSTRACT.** Families, traditional caretakers of its members, when not able to fulfill their role within the aged ones looking for a long-stay institutions for the elderly (LTCF) to meet such demand. Caring for the elderly in conditions of dependency and disability, even for professionals, brings exposure to physical and emotional overload, which can lead to high levels of stress. **Objective:** To determine the stress levels of professionals working in institutions for the elderly. **Method:** A descriptive exploratory, with the identification of forty-nine professionals from fourteen ILPI a municipality north of the state of Rio Grande do Sul, however only eighteen professionals from eight institutions agreed to participate. The project was approved by the Ethics Committee in Research of the University of Passo Fundo, Protocol No. 393/2011, an instrument was used self-report and the Inventory of Stress Symptom adult Lipp - ISSL and data were analyzed using the statistical package. **Results:** Of the eighteen professional, six were nurses and twelve nursing technicians, most with ten (55.6%) were female and single (a) variants aged between twenty-five and sixty, being the Most (66.6%) had high school or higher education incomplete. Most professionals have over five years of training and nine (50%) work in the institution in a short period of time, an average of six months to one year, seven (38.7%) carry a workload of 40 hours or more in the institution, nine (50%) are responsible for the care of ten or fewer seniors and fifteen (83.3%) have only this job. Concerning the level of stress, it was found that the three phases of LISS, was present only in resistance to impact five subjects (27.8%). **Conclusion:** It was found that the symptoms directly related to the stress of the professionals of the study were not significant and those who had symptoms present were limited to the resistance phase. Assume that this can be attributed to the fact that the team more autonomy and less pressure coming from the medical team and the family, which in this scenario are not permanently present. Thus, it is suggested that further studies with this evaluative framework in other contexts and with a broader sample.

**THE ELDERLY'S AGING PERCEPTION MARRIED AND WINDOWED** *Ralf Braga Barroso; Elaine Andrade Moura; Thamara Cunha Nascimento Amaral; Cláudia Helena Cerqueira Mármora*

**ABSTRACT.** 2010 Census data shows that the number of women is higher than men in almost all age groups above 60 years. The event of widowhood is considered a shattering moment in the person's life, being related to depressive symptomatology and anxiety. Even those that establish social ties seem not to get emotional compensatory benefits. The positive self-perception of the aging process is associated with a better functional health, over time, promoting a successful aging process. **Objective:** Therefore, this research looks to assess the perception of aging among widowed and married elderly who attend community centers. **Methods:** We apply a perception aging questionnaire with 28 questions, with answers' options raging from 1 - strongly disagree, to 6 - agree and the Mini-Mental State Examination to assessment the cognition. After, the participants were divided into two groups, the first consisting of 39 married elderly with a mean age 71.31 years (SD±7.49), and the second for 41 widowed elderly with a mean age 75.36 years (SD±8.27). The statistical analyze was done using SPSS 20.0. We used the Shapiro Wilk test in order to verify the behavior of distribution of variables and the Mann Whitney test for the analysis of the existence of statistically significant differences between groups (p<0.05). This research was realized at the Polo Cultural Enrichment for Third Age of the Federal University of Juiz de Fora (UFJF) and Community Center Dona Itália Franco, Juiz de Fora, Minas Gerais, Brazil. Every participant signed the consentient term and this study was approved by the Ethics Committee on Human Research of UFJF in the opinion 238,469/2013. **Results:** The variable perception of aging presented nonparametric distribution by Shapiro Wilke and the Mann-Whitney test rejected the null hypothesis that there is no difference between the two groups (p=0.04), resulting median values of 94 (group married) and 89 (group widows). **Conclusion:** We conclude that even the widow elderly who frequents community centers where various activity and social engagement are offered, presented a more negative aging perception than the married elderly. However, to assessment the real benefices of community centers in the life this elderly is necessary to compare them with widowed elderly who does not attend community centers.

**THE INFLUENCE OF FALLS IN SURVIVAL OF THE OLD CITY OF SÃO PAULO** *Gisele Patricia Duarte; Jair Lício Ferreira Santos*

**ABSTRACT.** With aging, changes occur in the biopsychosocial aspects of the elderly that can make them more fragile and generate instability and decreased ability to perform daily tasks (Duarte, 2007; Moriguti et al, 2003). A survey by the Estudo SABE pointed that fall is weakening factor for the elderly, but can also result from its fragile condition: loss of muscle strength, unsteady gait, decreased physical activity are risk factors for falls (Santos, Duarte, Lebrão & Duarte, 2010). Thus, the decline in the elderly may mark the beginning of frailty and death (Fabrício, Rodrigues & Costa JR, 2004). This study objective to investigate the impact of the fall in a given year, the survival of the elderly. We used data from the Estudo SABE (Health, Wellness and Aging), referring to elderly residents in the municipality of São Paulo in two collections, 2000 and 2006, and in 2143 obtained interviews in 2000 and 1115 in 2006 (Lebrão e Laurenti, 2005). Defined the variable “Fall” by its occurrence between 2000 and 2006 interviews. To perform the survival analysis, we used data of deaths by the end of 2006, giving a total of 649 deaths. To compare survival, we used Kaplan-Meier curves for the variables: age, sex and fall. It was observed that the absence of drop is sensitive to survival, that is, who shows no decline survives in greater proportion than the elderly who experienced falls. This relationship is even more evident for females over the age of 74 years and it crashed. This may be related to the role that women play in society, as do buy, pack house, factors that make it more vulnerable. The age variable is also sensitive to survival. Younger elderly live longer than older people, especially in males. Therefore, elderly women aged 60-74 years who reported no decrease in the course of one year have increased survival. Therefore, both age are sensitive to the fall survival and may be associated with increased intrinsic factors with increasing age, which can lead to falls and consequently death. This fact is even more pronounced for females. In conclusion, the importance of new studies and programs for preventions of falls in the elderly.

**THE RELATIONSHIP BETWEEN COGNITION, BODY IMAGE AND FUNCTIONALITY IN ELDERLY USERS FROM FAMILY HEALTH PRACTICES IN PORTO ALEGRE (BRAZIL)** *Raquel Rousselet Farias; Thais de Lima Rezende; Graziela Trindade Peña; Laura MBCRM da Rocha; Eduardo Lopes Nogueira; Irenio Gomes Filho; Vera Elisabeth Closs*

**ABSTRACT.** In the elderly, the functional capacity (FC) assessment can detect possible risks of dependency. In them, satisfaction with body image (BI) and its relationship with FC has not been investigated. This study, therefore, was designed to: examine cognitive function and BI perception, determine the performance on functional tests and investigate the association between cognition, BI and functionality in the elderly. This cross-sectional, analytical study was conducted on a random sample of 180 subjects from 10 Family Health Practices in Porto Alegre (RS, Brazil). Data were collected on demographic aspects (sex, age, marital status and education), cognition (Mini-Mental State Examination - MMSE), satisfaction with BI (Stunkard scale), lower (Sit/Stand - S/St) and upper limb strength (Hand Grip - HGS, Jamar^®^ dynamometer). The mean age was 68.1±6.3 years. Most participants were female (62.2%), with low education (4.5±3.4 years), dissatisfied with their BI (72.3%) and cognitively intact (85.6%). Notwithstanding, the prevalence of dementia was high (14.4%). The average HGS was 28.5 Kgf. The average score for the S/St was 8.9s. Gender, age, marital status proved independent of cognition and BI. Cognition was not associated with functionality or with BI. Seniors happy with their BI had significantly higher S/St and HGS results than the dissatisfied ones. In this sample, the prevalence of dementia was high, the lower limb strength was within the expected, but the upper limb strength was below. Most seniors were dissatisfied with their BI; those satisfied performed better at the functional tests. Cognition was not related to BI or functionality. Keywords: Aged, Aging, Body Image, Cognition, Dementia.

**TRAINING OF CAREGIVERS OF PATIENTS WITH ALZHEIMER'S FAMILY HEALTH STRATEGY** *Elaine Aparecida De Melo; Gylce Eloisa Panitz Cruz; Rejane Helena Nascimento Oliveira*

**ABSTRACT.** The aging process involves numerous cognitive and behavioral. Alzheimer's disease is the leading cause of dementia among family impact, mainly by ignorance. The training will enable greater accessibility of information to the caregiver about Alzheimer's disease thus contributing to better care and quality of life for both the caregiver and the elderly. **Objectives:** To train family caregivers of elders with Alzheimer's disease of Units of the Family Health Strategy in Divinópolis, Minas Gerais. Methodology and Description of Experience: The program will promote training of caregivers of elderly people with Alzheimer's. The training will be six modules, lasting six months will focus on several aspects of Alzheimer's disease. Program will be registered up to 50 caregivers of 18 units of the Family Health Strategy Divinópolis. In addition will be made home visits to assess by Barthel scale the degree of dependency of the elderly and relate it with the emotional burden of the caregiver, assessed by the scale Zariti. Make be a booklet with the modules covered in the training and distributed in healthcare facilities. **Results:** The program will promote training of caregivers of elderly es through deeducação health and development of educational material on the process of aging and Alzheimer's disease, symptoms, cognitive changes, evolution, improving the care provided by the caregiver to the elderly. Conclusion and Hypotheses: The program will provide a better quality of life for both the elderly Alzheimer carrier as to their caregiver. Relating emotional burden of the caregiver and the degree of dependence of the elderly.

**TROCAS INTERGERACIONAIS ENTRE CRIANÇAS E IDOSOS POR MEIO DE OFICINA CULINÁRIA EM INSTITUIÇÃO DE LONGA PERMANÊNCIA** *Ana Lucia Alves; Audrey Andrade Bertolini; Iracelia Munhoz; Mercia Gomes Rodrigues; Renata Firpo R. Medeiros; Gisele Monaco Dias; Roberto Dischinger Miranda*

**RESUMO. Introdução:** O relacionamento entre as gerações assumiu diferentes formas ao longo da história, sendo de fundamental importância na transmissão de valores e ensinamentos, promovendo assim, a coeducação e a preservação da memória cultural. **Objetivo:** Aproximar as gerações e estimular as trocas intergeracionais por meio de oficina culinária em Instituição de Longa Permanência para idosos (ILPI). **Métodos:** Estudo descritivo com abordagem qualitativa de dados. Incluímos as crianças do Centro Educacional Infantil da Liga Solidária no programa de atividade culinária realizada com residentes dependentes de uma ILPI. Ocorre em grupos de, no máximo, 8 idosos e 12 crianças de 3 a 4 anos, com frequência mensal e duração de 90 minutos; supervisionada por terapeutas ocupacionais, educadoras e nutricionista que escolhe receitas atrativas e nutritivas para a oficina. Os idosos realizam as receitas em conjunto com as crianças, ou seja, o idoso lê a receita e adiciona um item dos ingredientes e a criança outro, assim sucessivamente; efetuando o modo de preparo. Após a produção tem a oportunidade de saborearem o alimento. **Resultados:** Dos 53 idosos da ILPI, ocorreu a participação ativa e frequente de 16 residentes, com prevalência do sexo feminino, e 36 crianças. Durante a atividade as idosas se sentem de volta as suas experiências, demonstrando interesse e motivação nas etapas de realização e transferência de conhecimento para as crianças. As crianças ficam concentradas e atentas durante as oficinas, além de demonstrarem comportamento de respeito com as idosas. **Conclusão:** A integração das crianças na oficina culinária demonstrou ser uma boa forma de aproximação entre gerações, com a possibilidade de transmissão de valores, afeto e experiências.

**USO DE BENZODIAZEPÍNICOS E QUEDAS EM IDOSOS INSTITUCIONALIZADOS** *Danilo Ferreira Maia; Patricia de Abreu Lima; Victor Ma-noel Cintra; Almir Ribeiro Tavares Junior; Vítor Eugênio Ribeiro; Ana Luiza Figueiredo Campos; Marcus Renato Castro Ribeiro; Marina Buldrini Filogonio Seraidarian; André Lana Pimenta; Lalleinny Franth*

**RESUMO. Introdução:** Uma parte dos idosos brasileiros, sobretudo os que residem em casas para idosos, fazem uso de polifarmácia, associado aos inúmeros problemas de saúde que se acumularam ao longo da vida e àqueles que vieram com o envelhecimento. Os problemas de saúde fazem do idoso uma pessoa frágil e suscetível a enfermidades, que outrora não seriam problemas - gripes, diarreias e quedas, por exemplo. A suscetibilidade às quedas ainda aumenta quando alguns dos fármacos podem aumentar a fragilidade do idoso, como os benzodiazepínicos, largamente discutidos na literatura. **Objetivo:** Realizar uma análise comparativa entre os moradores do Lar de Idosos São José que fazem uso de benzodiazepínicos e aqueles que não utilizam esse medicamento, para verificar se a utilização desse fármaco resulta no aumento de quedas entre idosos. **Metodologia:** Os moradores do Lar de Idosos São José foram divididos em dois grupos: idosos que fazem uso de benzodiazepínicos e idosos que não fazem uso de benzodiazepínicos. Após a separação dos moradores em tais grupos, foram consultados os prontuários desses pacientes, para verificar a ocorrência de queda entre eles e comparar em qual dos grupos esse evento é mais frequente. **Resultados:** Dezesseis idosos fazem uso de benzodiazepínicos diariamente e, destes, oito apresentaram queda no último ano, uma porcentagem de 50%. Dentre os vinte e três idosos que não usam benzodiazepínicos diariamente, seis apresentaram quedas no último ano, correspondendo a 26,08% da população. **Discussão:** O uso de benzodiazepínicos podem gerar efeitos indesejados, os quais podem ocorrer mesmo com o uso de doses terapêuticas.

São eles: graus variados de tonteira, lassitude, tempo de reação aumentado, falta de coordenação motora, comprometimento das funções mental e motora, confusão, amnésia anterógrada, e alterações nos padrões de sono. Esses efeitos são os mais prejudiciais em idosos e constituem fator importante no aumento do risco de queda. **Conclusão:** O uso contínuo de benzodiazepínicos está associado ao aumento da frequência de quedas em idosos. Assim, é necessária a prescrição consciente dessa classe de fármacos aos idosos, a fim de evitar danos ao organismo, maiores gastos com a saúde e a invalidez ou asilamento precoces dos idosos.

**USO DO MONTREAL COGNITIVE ASSESSMENT TEST NO AMBULATÓRIO DA MEMÓRIA DA UNISUL EM RELAÇÃO A FATORES PREDITIVOS E DIAGNÓSTICO CLÍNICO** *André Junqueira Xavier; Carla Spido Marchioro; Luiza Salvador Schmid; Mateus Dressler De Espíndola; Rafael Coradin; Rafael Mazzini Baptista*

**RESUMO. Introdução:** O Montreal Cognitive Assessment (MoCA) é um teste rápido, prático e sensível para rastreio do Transtorno Cognitivo leve e demências. Ele aborda atenção e concentração, funções executivas, memória, linguagem, habilidades visuo-construtivas, conceituação, cálculo e orientação. A pontuação máxima é de 30 pontos. **Objetivo:** Determinar a variação do MoCA no Ambulatório da Memória, segundo variáveis sociodemográficas, de saúde e o diagnóstico clínico (Padrão ouro) de higidez, Transtorno Cognitivo Leve (TCL) monodomínio, TCL multidomínio e demência. **Métodos:** Estudo transversal, dados secundários, realizada análise bivariada por teste T de Student e análise multivarida por regressão linear (p<0,005). Desfecho pontuação bruta do MoCA. Censo da população em acompanhamento no Ambulatório da Memória (set 2012-jun 2013) no Ambulatório Médico de Especialidades (AME). Dados coletados por equipe de alunos do último ano de medicina, treinada e supervisionada, com escores validados no Brasil e diagnóstico clínico realizado após pelo menos duas consultas e após acesso a exames laboratoriais, de imagem e avaliação geriátrica ampla (AGA), revisado por médico especialista. Aprovado pelo CEP da UNISUL nº1663.07, recebeu recursos do CNPq, registro nº 563449/2010-1. Respeitados os princípios da Resolução 196/96 do CNS. Limitação: TCL dividido apenas em monodomínio e multidomínio*, subdivisão amnéstico ou não/amnéstico não analisado. **Resultados:** Foram avaliadas 139 pessoas, idade 68±9,3, MoCA 19,04±5,77. Análise bivariada: IMC>30 - 17,75±6,18 versus IMC≤30 19,88±5,3 (p=0,039); Sedentário (atividade física< 3 x sem.) -18,33±5,28 versus Não sedentário 20,30±5,94 (p=0,046); Escolaridade, 0-6 anos 17,88±5,84 versus 7-9 anos - 22,31±3,01 versus 10 anos ou mais

- 23,61±3,23 (p=0,000); Colesterol total ≥240mg/dl - 15,44±3,6 versus Colesterol total<240mg/dl 19,28±5,82 (p=0,014). Mode-lo multivariado: Escolaridade (Coef= -0,337, p=0,000), Colesterol total ≥240mg/dl (Coef= -0,147, p=0,069), limítrofe. Idade e gênero não significativos. A média do MoCA foi diretamente proporcional ao nível de gravidade do diagnóstico clínico: hígido - 25,74±2,32 versus TCL monodomínio* 21,63±2,51 versus TCL multidomínio* 16,6±3,09 versus Síndrome Demencial 10,70±3,29 (p=0,000). Idade e gênero não significativos. **Conclusões:** O moca teste se mostrou uma ferramenta adequada para apoiar a decisão médica no âmbito da população estudada.

### NEUROLOGIA

**BEHAVIORAL AND NEUROPSYCHIATRIC SYMPTOMS IN PATIENTS WITH DEMENTIA DUE TO ALZHEIMER'S DISEASE** *Fabricio Ferreira de Oliveira; Jose Roberto Wajman; Paulo Henrique Ferreira Bertolucci; Elizabeth Suchi Chen; Marilia de Arruda Cardoso Smith*

**ABSTRACT.** Behavioral symptoms are common in patients with dementia due to Alzheimer's disease (AD), and may impact independence. **Objective:** To quantify correlations between behavior and cognition in patients with AD. **Methods:** Patients with AD were evaluated for age of dementia onset, education, alcoholism, medications, mental state (MMSE, SMMSE), 10-item neuropsychiatric inventory (NPI), clinical dementia rating (CDR), 15-item clock drawing test (CDT), activities of daily living, caregiver burden, and APOE haplotypes (SNPs rs7412 and rs429358 assessed by way of Real-Time PCR reactions using TaqMan assays). Pear-son correlations were calculated for all variables. Mann-Whitney test was employed for group comparisons regarding APOE haplotypes, significance at p<0.05. **Results:** A sample of 217 patients was assembled. Mean age of dementia onset was 73.15±6.8 years-old, negatively correlated with quantity of medications, NPI, caregiver burden, alcoholism and CDT scores. Mean schooling was 4.21±3.7 years, negatively correlated with NPI, caregiver burden and alcoholism. Anxiety and apathy had the highest mean scores in the NPI, but irritability (r=0.66), aggression (r=0.59), disinhibition (r=0.58) and hallucinations (r=0.51) had the highest correlations to total scores. Apathy was negatively correlated to anxiety and delusions, while aggression had a strong correlation with irritability. The mean CDT score was 6.26±4.5, highly correlated with education, MMSE (r=0.68) and SMMSE (r=0.62) scores. All activities of daily living were strongly correlated to total scores, but bathing and dressing had the highest interrelation (r=0.72). The worst impairment in instrumental activities was for medications. Attention (r>0.7) and immediate recall (r=0.61) had the highest correlations to total MMSE scores. Caregivers were usually less embarrassed when patients asked for more help. Earlier onset of dementia was correlated with APOE4+ in patients with CDR=2.0 (p=0.019) and with the E4/E4 haplotype in patients with CDR=1.0 (p=0.007). APOE haplotypes had no significant association with behavior, but caregiver burden was higher for APOE4- patients with CDR=1.0 (p=0.002). **Conclusion:** This study is cross-sectional, with no power to establish causal relations. However, most instruments for cognitive assessment had worthy correlations with evolution of dementia and behavioral symptoms. APOE haplotypes are critical for age of dementia onset, but not for neuropsychiatric performance.

**ESCLEROSE MÚLTIPLA DE INICIO TARDIO: UM ESTUDO DE CORTE TRANSVERSAL** *Eduardo Cardoso; Graziella Aguiar; Soraya Lustosa; Gizelle Munduruca; Eugenia Paiva*

**RESUMO.** O início da esclerose múltipla (EM) após os 50 anos é pouco frequente e representa um desafio diagnóstico. O objetivo do presente estudo foi analisar a prevalência, apresentação e características clínicas do início tardio da Esclerose Múltipla. **Método:** Um estudo retrospectivo do tipo corte transversal. realizado no NAPEM/UFBA ( Núcleo de Apoio ao Paciente com Esclerose Múltipla da UFBA), centro de referencia no estado da Bahia para tratamento desta enfermidade. VARIÁVEIS: Diagnóstico de EM foi estabelecido de acordo com critérios de McDonald e confirmada por ressonância nuclear magnética (RNM), utilizando prontuários médicos da nossa unidade. De início tardio MS foi definida como a primeira apresentação dos sintomas clínicos após a idade de 50 anos. Para cada paciente, idade, gênero, forma clínica, o curso da doença, o envolvimento neurológico, duração da doença, incapacidade neurológica avaliada pelo EDSS foram analisadas. **Resultados:** Dos 420 pacientes com EM, 15 (3,5%) foram diagnosticados como sofrendo de início tardio da EM. A idade média de início foi de 54,5±3,1, faixa de 50 a 62 anos. Feminino em relação ao masculino era 1.76:1. A média de duração da doença foi de 6,5 anos, variando de 2 a 12 anos. Em 50% dos pacientes,

o curso da doença foi recorrente-remitente. Sintomas motores estavam quadro neurológico mais comum no início (63,3%). Após uma duração média da doença de 7,0 anos, houve um aumento significativo no envolvimento do esfíncter vesical e cerebelar. **Conclusões:** O início tardio da EM não é raro como se pensava. Embora a apresentação neurológica no início é semelhante à de adultos jovens, a progressão para a deficiência sugere ser mais rápida e um curso progressivo primária é mais prevalente.

**FUNCIONALIDADE E DESEMPENHO COGNITIVO NA DEMÊNCIA FRONTOTEMPORAL VARIANTE COMPORTAMENTAL** *Thais Bento Lima da Silva; Valéria Santoro Bahia; Viviane Amaral Carvalho; Henrique Cerqueira Guimarães; Paulo Caramelli; Márcio Balthazar; Benito Damasceno; Cássio Machado de Campos Bottino; Sônia Maria Dozzi Brucki; Ricardo Nitrini; Mônica Sanches Yassu*

**RESUMO.** Existem poucos estudos sobre alterações funcionais na Demência Frontotemporal variante comportamental (DFTvc). Objetivou-se no presente estudo: 1. Caracterizar o desempenho funcional e cognitivo de pacientes com diagnóstico prévio de DFTvc, atendidos em ambulatórios de Neurologia e Psiquiatria e compará-los a pacientes com Doença de Alzheimer (DA) e controles saudáveis; 2. Examinar a correlação entre o desempenho em escalas funcionais (DAFS-BR, DAD e PFAQ) e o desempenho cognitivo; 3. Avaliar a acurácia diagnóstica da DAFS-BR para a detecção da DFTvc e da DA. **Métodos:** 96 indivíduos com idade igual ou superior a 45 anos, com escolaridade formal acima de dois anos. Destes, 31 diagnosticados com DFTvc, 31 pacientes com DA e 34 eram adultos saudáveis pareados aos pacientes com DFTvc e DA para idade e escolaridade. Variáveis: sociodemográficas e clínicas; Escala de Depressão Geriátrica (Geriatric Depression Scale - GDS) de 15 itens, Addenbrooke Cognitive Examination-Revised (ACE -R), Mini Exame do Estado Mental (MEEM), Executive Interview (EXIT-25), Direct Assessment of Functional Status (DAFS-BR). O protocolo dos acompanhantes conteve o Pfeffer Functional Activities Questionnaire (PFAQ), Disability Assessment for Dementia (DAD), e a Escala de Avaliação Clínica da Demência (CDR). **Resultados:** Pôde-se observar que os grupos com DA e com DFTvc apresentaram desempenho equivalente na DAFS-BR, entretanto sendo inferior ao grupo controle. Em relação à DAD, o grupo com DFTvc teve pior desempenho nos domínios de Iniciação e Planejamento/Organização na DAD, comparado aos idosos com DA, sugerindo que a dependência na DFTvc é mais acentuada. A pontuação mais elevada na PFAQ, sugeriu que a dependência na DFTvc é mais acentuada. No grupo com DFTvc as correlações entre variáveis cognitivas e funcionais foram significativas e de grande magnitude. Os dados de acurácia para a DAFS-BR sugerem que a escala pode auxiliar na identificação das demências, apresentando limitações no diagnóstico diferencial entre os subtipos, a DAFS-BR tinha uma sensibilidade de 54,8% e especificidade de 64,5% para separar DA de DFTvc. Considerações finais: Os resultados apresentados sugerem que indivíduos com DFTvc apresentam maior prejuízo funcional, quando comparados com participantes com DA e adultos saudáveis. Destaca-se a importância da avaliação funcional de pacientes com suspeita de DFTvc, devido à relevância destas alterações para o diagnóstico e manejo clínico deste subtipo de demência.

**FUNCTIONAL DISCONNECTION OF INTRINSIC NETWORKS IN MILD ALZHEIMER'S DISEASE** *Marina Weiler; Aya Fukuda; Lilian H. P. Massabki; Tátila M. Lopes; Alexandre R. Franco; Benito P. Damasceno; Fernando Cendes; Marcio L. F. Balthazar*

**ABSTRACT.** Recent advances in the neuroimaging of AD have highlighted dysfunctions in functionally connected networks (FCNs), especially the default mode network (DMN). Therefore we aimed to identify different FCNs - Default Mode, Language, Executive Functions and Visuospatial networks - in mild AD patients, by means of resting-state functional connectivity MRI (rsfcMRI), and also to correlate them to cognitive scores. We evaluated 22 patients with mild AD (mean age: 73.4 years-old; 16 women) and 26 healthy controls (mean age: 70.9 years-old; 20 women) matched for gender, age and education. All subjects underwent a 10-minute task-free fMRI at 3.0T. Images were pre-processed by applying slice-time and motion corrections algorithms, removing linear trends, smoothing with a 6 mm FWHM Gaussian kernel, bandpass filtering (0.008 to 0.1 Hz) and normalization. Six parameters of head motion, cerebrospinal fluid and white matter time series were regressed as nuisance variables. We aimed to identify different FCNs: DMN, language, executive functions and visuospatial skills. Seed-based (radius=3mm) functional connectivity was calculated by placing a seed in DMN (posterior cingulate cortex ; 0,-51,15); Language networks: Broca's (-48,15,0) and Wernicke's (-48,-36,12) areas; Executive functions: Right (45,18,48) and Left (-45,18,48) dorsolateral prefrontal cortex (DLPFC); Visuospatial skills: R (36,-84,-3) and L (-36,-84,-3) associative visual cortex. Differences between controls and patients were estimated by nonparametric permutation tests (1000 permutations) and a simple regression analyses was done between individual connectivity maps for the different FCNs and the scores on cognitive tests. Mild AD patients have functional alterations in some resting-state networks: increased connectivity in Executive Function Network, and decreased connectivity in DM and Language Networks; Connectivity of Visuospatial Network does not differ between mild AD and controls; DMN connectivity correlates to episodic memory (delayed recall); Language Network connectivity (Wernicke's area) correlates to semantic verbal fluency scores. Alterations in FCNs in AD, even in early phase, are not restricted to DMN. Other FCNs related to language and executive functions may be hypo or hyperconnected (an alteration which possibly reflects a compensatory mechanism). We just found correlation between DMN connectivity and episodic memory performance, and Language Network and semantic verbal fluency.

**HIDROCEFALIA DE PRESSÃO NORMAL: COMO AVALIAR OBJETIVAMENTE A RESPOSTA AO TAP TEST?** *Helena Alessi; Fernando Vieira Pereira; Carlos Senne; Sandro Luiz De Andrade Matas*

**RESUMO. Introdução:** A indicação de pacientes com hidrocefalia de pressão normal - HPN, ou hidrocefalia crônica do adulto, para tratamento cirúrgico suscita dúvidas ainda hoje. O diagnóstico preciso e a ponderação entre riscos e benefícios associados à colocação de sistema de derivação precisam ser discutidos caso a caso, permitindo a indicação terapêutica adequada e decisão cirúrgica segura. O Tap Test - TT é utilizado mundialmente por ser um exame realizado em ambiente ambulatorial, sem a necessidade de equipamentos especializados e com poucos riscos para o paciente. Costuma ser citado na literatura como exame capaz de proporcionar uma situação momentânea similar à da derivação, com alto valor preditivo positivo (73 a 100%). No entanto, o TT apresenta baixo valor preditivo negativo (23 a 42%) indicando a necessidade de outros testes mais sensíveis caso a suspeita clínica seja consistente. **Objetivo:** Avaliar objetivamente a resposta de idosos com suspeita diagnóstica de HPN após TT. **Métodos:** Nosso protocolo de avaliação objetiva do TT foi idealizado para ser realizado em dois dias consecutivos, sem internação. A avaliação neuropsicológica consiste em testagem de diferentes domínios cognitivos, como velocidade de processamento, atenção e fluência. A avaliação cinemática da marcha, em um percurso de 10 metros, sem obstáculos, visa mensurar importantes parâmetros que podem responder positivamente ao TT. A reavaliação é realizada aproximadamente 2 horas após a punção de grandes quantidades de LCR. **Resultados:** Analisamos o resultado de 28 pacientes. A idade média dos indivíduos foi de 75,36 anos (mínimo de 63 e máximo de 87 anos). Na avaliação neuropsicológica, a atenção melhorou 7% (p=0,127); a velocidade de processamento melhorou em 10% (p=0,17); e a fluência melhorou em 2% (p=0,687). Na avaliação cinemática da marcha, a velocidade média da marcha aumentou em 13% (p=0.043); o tempo para o indivíduo percorrer o percurso diminuiu 12% (p=0.032);; o número de passos necessários para cumprir o percurso reduziu 35% (p=0.005); e o número de passo no retorno reduziu-se em 6% (p=0.120). **Conclusões:** A análise dos dados acima nos permite concluir que a marcha é mais susceptível à melhora após a punção liquórica. Velocidade de processamento e número de passos dados no percurso foram, respectivamente, as variáveis neuropsicológicas e cinemáticas da marcha que apresentaram alterações mais importantes.

**NONFLUENT PRIMARY PROGRESSIVE APHASIA WITH BIOMARKERS COMPATIBLE WITH THE ALZHEIMER DISEASE: CASE REPORT** *Ricardo Krause Martinez de Souza; Jonilson Antonio Pires; Orjana Scheila Gadotti; Ricardo Lehtonen Rodruigues Souza; Pedro André Kovacs; Daiane Priscila Simão Silva; Gleyse Freire Bono*

**ABSTRACT.** The Primary Progressive Aphasia (PPA) is a neurodegenerative disease wherein language remains the most impaired domain throughout the course of the illness and other cognitive domains are preserved in the first two years of the disease. Current classification of PPA is divided into three subtypes: non-fluent, semantic variant and logopenic variant. The diagnosis is given from the clinical, neurological and neuropsychological evaluation. The aim of this work was to describe a clinic case of PPA nonfluent with evidence of biomarkers compatible with the Alzheimer's disease (AD). In this case report, a 73 years old man had onset of symptoms in 2010 characterized by progressive alteration of exclusive language. Speech nonfluent with syntax and repeat errors, with the understanding preserved. The patient has no relevant pathological antecedent, but he has familiar history of dementia, father and two siblings. Blood tests to complementary clinical investigation of dementia were normal. Neuropsychological assessment showed no loss in any other cognitive function, except for the language. Magnetic resonance imaging had normal and positron emission tomography of the brain showed marked hypometabolism in the left temporal-parietal region, often found in nonfluent PPA. The level research of Tau-T, P-tau and βA-42 biomarkers in cerebrospinal fluid was consistent with AD. In conclusion, the βA-42 decrease and Tau-T, P-tau increase can have a high sensibility and specifity for the AD diagnosis when clinical findings suggest AD. Our case report draws attention to a typical clinical picture of varying nonfluent APP with the presence of positive biomarkers for the AD.

**PREDICTORS OF THE INTENSITY OF MEMORY COMPLAINTS AMONG NON-DEMENTED ELDERLY SUBJECTS** *Fábio Henrique de Gobbi Porto; Maira Okada de Oliveira; Lívia Spíndola; Patrícia Helena Figueiredo do vale Capucho; Ricardo Nitrini; Sonia Maria Dozzi Brucki*

**ABSTRACT.** Background: Memory complaints (MC) are very frequent among elderly population. Previous studies have shown a great association of MC with depressive and anxiety symptoms. The objective of the study was to evaluate what variables were more associated with the severity of MC in non-demented elderly subjects. **Methods:** We evaluated 106 subjects diagnosed as mild cognitive impairment (MCI) and subjective memory complainers (SMC). Diagnosis was based on a clinical interview and neuropsychological assessment. Patients with depression and dementia were excluded. We used the scores of the Mini Mental State Examination (MMSE), Brief Cognitive Battery (BCB), letter “P” fluency (LPF), late-delayed recall (LDR) of MMSE words, Geriatric Depression Scale (GDS) and Geriatric Anxiety Inventory (GAI) as predictor of the Memory Complaint Questionnaire (MAC-Q). Principal components analysis (PCA) was conducted to assess how the variables clustered creating factors. After that, the factors were used as independent variables of the MAC-Q scores in a multivariate regression. All data analyses were performed using SPSS version 17.0 (SPSS, Inc., Chicago IL). **Results:** We included 90 MCI and 16 SMC. PCA disclosed 3 factors, based on the eigenvalues over 1 criteria. The first factor loaded on memory scores, the second on general cognition/language/executive tests and the third on GAI and GDS. Linear regression showed that age and schooling did not significantly predict MAC-Q scores, F(2,96)=0.633, p=0.533. When the factors were added, it significantly improved the prediction, R2 change=0.21, F(3,93)=8.57, p=0.000. The entire groups of variables significantly predicted MAC-Q scores, F(5,93)=5.45, p=0.000, adjusted R2=0.08.With this combination of predictors, only factors 1 and 3 (memory and GDS/GAI) significantly contributed to the prediction, with beta values of -0.23 and 0.38, p=0.02 and 0.000, respectively. **Conclusion:** The intensity of MC measured by the MAC-Q is best predicted by factors loaded on memory tests and depression and anxiety scales scores.

**REVISÃO SISTEMÁTICA SOBRE USO DA TOMOGRAFIA POR EMISSÃO DE PÓSITRONS (PET-CT) NO DIAGNÓSTICO DIFERENCIAL DA DOENÇA DE ALZHEIMER (DA)** *Artur Francisco Schumacher Schuh; Jonas Alex Morales Saute; Cleusa P. Ferri; Tiago V. Pereira; Jeferson G. Fernandes*

**RESUMO. Introdução:** A PET-CT é uma técnica de imagem com potencial no diagnóstico da demência. No entanto, não são claras as evidências que justifiquem seu uso. **Métodos:** Revisão sistemática para avaliar evidências sobre a PET-CT no diagnóstico de doença de Alzheimer (DA) e outros tipos de demências. Foram realizadas pesquisas por artigos originais nas bases de dados Medline, Embase, Lilacs, Google Scholar, Cochrane Registry of Controlled Trials e ClinicalTrials.gov (até 01/09/2012). Selecionou-se estudos originais e comparativos cujos pacientes: 1) Apresentaram diagnóstico provável de DA segundo os critérios do NINCDS-ADRDA ou pelos critérios do DSM-IV; 2) Diagnóstico de demência leve a moderada; 3) Utilizaram como referência padrão o diagnóstico clínico com seguimento de longo prazo ou diagnóstico anatomopatológico. Foram contemplados quatro tipos principais de demência: doença de Alzheimer (DA), demência por corpos de Lewy (DCL), demência fronto-temporal (DFT) e demência vascular (DV). **Resultados:** Foram incluídos 7 estudos, todos utilizando PET [18F] fluorodesoxiglicose (FDG), do tipo caso-controle com padrão-referência o diagnóstico clínico, totalizando 707 indivíduos. Para o diagnóstico diferencial entre DA e DLB, dois estudos (n=267) estimaram sensibilidade entre 75% e 98% e especificidade entre 71% e 100%. Para DA e DV, dois estudos (n=134) apresentaram sensibilidade entre 70% e 100% e especificidade de 100%. Para DA e DFT, em dois estudos (n=332) a acurácia variou entre 80 e 97%. Sensibilidade e especificidade não foram relatadas. Por fim, a comparação entre DA e demências não-Alzheimer foi avaliada em três estudos (n=497) onde a sensibilidade variou entre 65% a 98%, e a especificidade variou entre 69% e 81%.Três estudos compararam a PET com outras modalidades de exame complementar. A RMN (dois estudos,n=130) e marcadores bioquímicos (um estudo, n=95) apresentaram a mesma acurácia que a PET. A combinação da PET e da RMN promoveu um incremento modesto na acurácia diagnóstica, entre 8% a 11.4%. Não foram relatados dados sobre segurança da tecnologia. **Conclusão:** Os sete estudos incluídos nesta revisão têm número limitado de pacientes, alto risco de viés, baixa validade externa e apresentam estimativas possivelmente superestimadas. A evidência disponível com relação ao papel da PET no diagnóstico diferencial das demências tem generalização limitada.

**TAP TEST COM RESPOSTA TARDIA EM PACIENTE COM HIDROCEFALIA DE PRESSÃO NORMAL - RELATO DE CASO** *Helena Alessi; Fernando Vieira Pereira; Paulo Henrique Ferreira Bertolucci; Orestes Paulo Lanzoni; Carlos Senne; Sandro Luiz De Andrade Matas*

**RESUMO. Introdução:** Hidrocefalia de Pressão Normal - HPN, também conhecida por Hidrocefalia Crônica do Adulto, é uma síndrome caracterizada por alterações de marcha, comprometimento cognitivo e incontinência urinária, sendo citada como uma das causas potencialmente tratáveis de demência. Testes funcionais, como o Tap Test - TT, são úteis na seleção de pacientes para a cirurgia de derivação. O valor preditivo positivo do TT varia de 73 a 100%. No entanto, não há um consenso na literatura sobre

o intervalo ideal para melhor identificar as alterações decorrentes da punção de grandes quantidades de LCR. A maioria dos estudos publicados sugere reavaliação poucas horas após o TT, em aproximadamente 2h após a punção. **Método:** Uma paciente sob investigação diagnóstica para HPN foi avaliada segundo o protocolo do grupo de HPN do Laboratório Senne Liquor. O protocolo inclui avaliação objetiva da marcha e de aspectos neuropsicológicos antes da punção lombar, com drenagem de 40ml de líquor, e após 2 horas da punção. Foram realizados dois TT ambulatoriais, a pedido do médico, com intervalo de sete semanas entre eles. No primeiro exame a reavaliação ocorreu 2h após a punção. No segundo TT os mesmos testes foram aplicados aproximadamente 48h após nova punção. A primeira avaliação (avaliação de base) foi utilizada como parâmetro para comparação entre os resultados pós-punção nas duas ocasiões. **Resultados:** Na avaliação neuropsicológica houve melhora de 33% na fluência verbal após 2h e 167% após 48hs. O desempenho nos testes de marcha após 2h da primeira punção foram idênticos aos obtidos na avaliação de base em relação ao número de passos, velocidade e tempo de percurso. A reavaliação após 48h apontou redução de 84% no tempo de percurso em relação à avaliação de base, 57% no número de passos e aumento de 125% na velocidade média da marcha. Outras melhoras qualitativas foram observadas no desempenho da marcha e da cognição. **Conclusão:** Apesar da maioria dos pacientes apresentar melhora nas primeiras 24h após a punção, um número pequeno deles apresenta melhora tardia. É importante a valorização de relatos de melhora tardia e avaliação objetiva do desempenho, mesmo após 24h do procedimento.

**TARDIVE DYSKINESIA IN THE ELDERLY, THE USE OF ANTIPSYCHOTICS: A CASE STUDY** *Kamilly Farah Cardoso Martins*

**ABSTRACT.** M.M.N, woman, 71, reported long history of depression and memory deficits. Using risperidone two years ago. There are about nine months developed involuntary movements of the mouth, difficulty in swallowing. Neurological examination reveals rapid movements, repetitive and stereotyped, involving oral, buccal and lingual areas. **Objective:** Is to highlight the importance of early diagnosis of tardive dyskinesia and its proper management. **Method:** A case report of a geriatric outpatient clinic. **Results:** After the gradual withdrawal of risperidone, the symptoms have reduced considerably over a period of two months. **Conclusion:** Tardive dyskinesia, in the case cited, orobucolingual dyskinesia, is a iatrogenic syndrome of persistent abnormal involuntary movements that occur as a complication of drugs that block dopamine receptors. The diagnosis of tardive dyskinesia is based on the patient's exposure to a dopamine receptor blocker in the six months prior to the beginning of the movement and persistence of the movement for a month after the patient stops taking the causative agent. The first step is remove the offending drug. If it is necessary to treat the symptoms may be required depleting drugs of dopamine receptors, such as reserpine. The knowledge of this complication deserves attention due to the frequent use of anti-psychotics in geriatric clinics for the management of behavioral disturbances associated with dementia.

### OUTROS

**A DIFICULDADE ENFRENTA PARA O REQUERIMENTO DE CURATELA DOS PACIENTES COM DEMÊNCIA ATENDIDOS EM DOMICILIO** *Aliciana B. R. Oliveira; Letícia Andrade; Lilian A. G. Maria*

**RESUMO. Introdução:** Composta por uma equipe multiprofissional, que atua de forma interdisciplinar, o NADI, através de vários profissionais como médicos, enfermeiros, fisioterapeuta, nutricionistas, psicólogos e assistentes sociais, busca atender os pacientes e familiares de forma integral e efetiva, afim de, promover, restaurar e manter o conforto, a função e a saúde dos pacientes. **Objetivo:** Verificar se todos os pacientes atendidos pelo NADI acometidos por doenças que os tornam incapazes de exercer atos da sua vida cível, já possuem a curatela, uma vez que, torna-se necessário e obrigatório a instituição de um representante legal, que possa realizar a gestão de seus bens e benefícios. Material e **Métodos:** Dados extraídos através de levantamento de prontuários dos pacientes atendidos pelo Núcleo de Atendimento Domiciliar Interdisciplinar. **Resultados:** O levantamento mostrou que, cerca de 29,70% dos pacientes apresentam o diagnóstico de demência (Alzheimer e Parkisionismo), e 22,77% apresentam diagnóstico de Acidente Vascular Cerebral (com comprometimentos severos de ordens neurológicas). Sendo assim, a equipe de Serviço Social, observou que embora mais de 52,47% dos pacientes atendidos, se apresentam incapacitados para exercer atos da sua vida cível, apenas 12,87% possuem a Curatela. As Assistentes Sociais verificaram que parte das famílias destes pacientes deixa de dar entrada ao Processo de Curatela, devido ao seu alto custo. **Conclusão:** O levantamento realizado pelo Serviço Social elucidou que cerca de 39,60% dos pacientes atendidos atualmente pela equipe interdisciplinar do NADI, não possuem Curatela por razões financeiras. Sendo assim, é imprescindível a atuação do profissional do Serviço Social, uma vez que o mesmo possui condições de articular através de suas práticas e ações, meios que possa viabilizar o aces-so desta demanda ao documento em questão.

**ARTE E MEDIAÇÃO: PERSPECTIVAS DO SERVIÇO SOCIAL** *Vera Núbia Santos.; Maria Lidiane Mendonça de Jesus; Maria Naislaine de Jesus Santos; Taiane Almeida do Nascimento*

**RESUMO.** Este estudo tem como objeto de análise a aproximação do Serviço Social ao debate sobre arte e trabalho profissional, tendo por base os trabalhos publicados em eventos nacionais da área - Congresso Brasileiro de Assistentes Sociais e Encontro Nacional de Pesquisadores em Serviço Social - no período de 2000 a 2010. Observou-se, por meio de levantamento bibliográfico, que a arte configura-se uma atividade emancipadora do ser social e está inscrita no campo das mediações do trabalho profissional. Para tanto, fez-se necessário conhecer os eixos temáticos, para compreender o “lugar” da arte nesses eventos, bem como mapear os trabalhos publicados no sentido de conhecer sua importância para a profissão. Os resultados obtidos delineiam que a apropriação da arte é um caminho na intervenção e na pesquisa no Serviço Social, como tema inerente à categoria mediação. PALAVRASCHAVE: Arte, Serviço Social, Mediação. ABSTRACT This work has as its analysis object the approach of the social work to the debate about art and professional work, supporting its bases on published papers in national events in the area such as the Congresso Brasileiro de Assistentes Sociais and Encontro Nacional de Pesquisadores em Serviço Social both between the period of 2000 and 2010. To develop the research it was necessary to know the guiding themes in order to understand the place of the art in these events and also to be able to map the development of the work by region. The results obtained depict the art appropriation as a way in the intervention and in the investigation as theme in the mediation category. KEY-WORDS: Art, Social Service, Mediation.

**CUIDADOS FÚTEIS: POSIÇÃO DOS FAMILIARES ORIENTADOS X RESISTÊNCIA DAS EQUIPES. RELATOS A SEREM OBSERVADOS** *Letícia Andrade; Naína Mendes Sanches; Angélica Massako Yamaguchi; Keila Tomoko Higa-Taniguchi; Ivone Bianchini de Oliveira*

**RESUMO. Introdução:** Tratamento fútil ou futilidade terapêutica são termos utilizados em cuidados paliativos e se referem às ações desnecessárias ou não indicadas para pacientes em estágios avançados de doenças. Estas ações não apresentam benefícios e representam, quase sempre, malefícios para os pacientes. **Objetivo:** Descrever, na perspectiva do familiar cuidador, a resistência oferecida pelas equipes de CP quanto a não indicação de cuidados fúteis no final da vida. Eixo da Pesquisa População idosa e cuidados paliativos. **Metodologia:** Necrópsia verbal: entrevista com cuidadores de idosos com diagnósticos na declaração de óbito (DO) que sugerem a necessidade de CP; informações das DO de idosos falecidos na capital, período de 01/01 a 29/03 de 2010, (Secretaria Municipal de Saúde de São Paulo). Realizadas 125 entrevistas; dados qualitativos, destes 15,2% (19) apresentavam doenças neurodegenerativas (demências por Alzheimer, Vascular e Corpos de Lewy; doença de Parkinson) e 16.8% (21) AVE. **Resultados:** Sobre os 19 pacientes com demência em fase final, cuja faixa etária variou de 81 a 102 anos, 09 familiares apontam para insistência das equipes de saúde por tratamentos fúteis, apesar do posicionamento das famílias pela sua não realização, destacando-se: entubação ou “ressuscitação”/reanimação cardíaca no final da vida, hemodiálise em pacientes não contatctuantes, informações inadequadas ou insuficientes por parte das equipes e insistência para a institucionalização mesmo a família tendo ciência de que o cuidado no final da vida podia ser realizado na residência. [trechos dos relatos serão apresentados no poster]. **Conclusão:** Tais dados sugerem que nem sempre as equipes estão devidamente preparadas para perceber e respeitar o processo de finalização da vida em pacientes com demência avançada, o que leva invariavelmente à futilidade terapêutica acarretando em maior sofrimento para o paciente e família.

**EXERCÍCIOS FÍSICOS VERSUS ATIVIDADES FÍSICAS: RESPOSTAS DE PARÂMETROS ANTROPOMÉTRICOS, CAPACIDADE FÍSICA E CAPACIDADE FUNCIONAL DE MULHERES COM IDADE SUPERIOR A 55 ANOS**

Clodoaldo Antônio De Sá; Vanessa da Silva Corralo; Fernanda Gollo

**RESUMO.** Atualmente, a noção de que o envelhecimento cronológico está inevitavelmente associado a perdas cognitivas e funcionais está bastante modificada. Nesse sentido, os estudos, bem como a avaliação de processos, técnicas e métodos voltados para recuperação, manutenção e promoção da autonomia do idoso são fundamentais para o planejamento de políticas e ações de intervenção. Dessa forma, o presente estudo teve por objetivo comparar a capacidade cognitiva, capacidade funcional e parâmetros antropométricos entre mulheres praticantes de exercícios físicos e praticantes de atividades físicas não sistematizadas. Foram estudadas 17 mulheres participantes do grupo de idosas da comunidade Santa Augusta no Município de Erechim-RS, sendo oito participantes de um programa de exercícios físicos (EF) duas vezes por semana (Idade=61,25±5,04 anos; Massa corporal=67,00±9,86 kg) e nove participantes de um programa de atividades físicas (AF) não sistematizadas (Idade=67,22±7,76 anos; Massa corporal=74,78±9,60 kg) com igual frequência semanal. Todos os sujeitos da amostra frequentam as respectivas atividades em seus grupos a um período não inferior a cinco anos. A análise dos dados não evidenciou nenhuma diferença (p >0,05) entre os grupos de EF e AF para as variáveis antropométricas: massa corporal, índice de massa corporal, somatório de dobras cutâneas, circunferência de abdômen e quadril e relação cintura/quadril. No entanto, a maior parte das praticantes de AF foi classificada na categoria sobrepeso (em função do IMC) em relação as praticantes de EF (67 e 25%, respectivamente). Tanto os sujeitos do EF quanto do AF, atingiram escores máximos na Escala de Barthel e foram classificados como completamente independentes. A avaliação da capacidade cognitiva através do Mini Exame do Estado Mental não evidenciou diferenças significantes (p >0,05) entre as praticantes de EF e AF (MEEM=17,64±2,07 e 15,89±3,26, respectivamente). Pode-se concluir que em longo prazo a prática de exercícios ou atividades físicas duas vezes por semana produzem respostas semelhantes em relação à capacidade cognitiva e funcional e aos parâmetros antropométricos avaliados no presente estudo.

**POLIFARMÁCIA E USO DE PSICOFÁRMACOS EM IDOSOS** *Karine Schwaab Brustolin; Vanessa da Silva Corralo; Clodoaldo Antônio De Sá; Clenise Liliane Schmidt; Marina Winckler*

**RESUMO.** A polifarmácia (polimedicação) constitui hoje uma epidemia entre os idosos, devido ao aumento exponencial da prevalência de doenças crônicas e as sequelas que acompanham

o avançar da idade. Essa população é mais suscetível aos efeitos colaterais dos medicamentos, que podem inclusive incrementar patologias já existentes, sendo que o risco de reações adversas aumenta proporcionalmente ao número de fármacos prescritos. Este estudo teve como objetivo avaliar a polimedicação e o uso de psicofármacos em idosos de ambos os sexos residentes na Região Extremo-Oeste de Santa Catarina. Foram entrevistados um total de 543 indivíduos, sendo 190 do sexo masculino (idade: 68,56±7,39 anos) e 353 do sexo feminino (idade: 68,15±7,18 anos), residentes na Região Extremo-Oeste de Santa Catarina. Todos os idosos trouxeram, no dia da avaliação, os medicamentos que faziam uso. Os dados obtidos no estudo demonstraram que 64,7% dos homens utilizam dois ou mais medicamentos associados, comparados a 57,5% das mulheres. Embora o consumo de medicamentos seja maior entre as mulheres, o uso de cinco ou mais fármacos foi maior no sexo masculino (12,6%) que no feminino (9,9%). Com relação ao uso de psicofármacos não houve diferença significativa na prevalência entre idosos do sexo masculino e feminino (±13%), entretanto a maioria destes faz uso concomitante com fármacos de outras classes farmacêuticas, deixando-os susceptíveis às interações medicamentosas. Os dados obtidos sugerem que apesar do uso da polimedicação na população em estudo, os valores foram menores quando comparados aos encontrados em outras regiões do país. Entretanto, o risco de efeitos adversos e interações medicamentosas associadas à polimedicação e uso combinado com psicofármacos representa um alvo importante na área da geriatria.

**PREVALÊNCIA DE DOENÇAS CRÔNICAS EM IDOSOS RESIDENTES NA REGIÃO EXTREMO-OESTE DE SANTA CATARINA** *Clenise Liliane Schmidt; Vanessa da Silva Corralo; Clodoaldo Antônio De Sá; Karine Schwaab Brustolin*

**RESUMO.** O perfil brasileiro de morbimortalidade vem sendo alterado pelo rápido envelhecimento populacional, onde o cenário caracterizado por uma população jovem, com maior incidência de doenças infecciosas, transformou-se em uma população mais envelhecida, onde predominam doenças crônico-degenerativas. O propósito deste trabalho foi avaliar as doenças crônicas que mais acometem idosos de ambos os sexos residentes na Região Extremo-Oeste de Santa Catarina. Para isso, foram avaliados um total de 543 indivíduos, sendo 35% do sexo masculino (idade: 68,56±7,39 anos) e 65% do sexo feminino (idade: 68,15±7,18 anos), residentes na Região Extremo-Oeste de Santa Catarina, através da aplicação de um questionário semi-estruturado do qual um dos objetivos foi a avaliação das doenças diagnosticadas e referidas pelos sujeitos. A análise dos dados evidenciou que 100% dos idosos entrevistados relataram ter ao menos uma doença crônica. No sexo masculino as doenças que apresentaram maior prevalência foram: hipertensão arterial (50,53%), seguida por problemas osteoarticulares (32,63%), cardiopatias (18,42%), tumores malignos de próstata (17,89%) e diabetes (13,62%). Para

o sexo feminino verificou-se que a doença com maior prevalência foi hipertensão arterial (75,07%), seguida por problemas osteoarticulares (39,94%), depressão (24,93%), cardiopatias (23,23%) e diabetes (22,66%). Com base nos dados obtidos, verificamos a alta prevalência de doenças crônicas na população idosa, principalmente a hipertensão arterial e a depressão em mulheres. Espera-se que os resultados obtidos nesse estudo possam contribuir para a adoção de medidas preventivas que visem diminuir os índices de doenças crônicas nessa população.

**QUEIXA DOS IDOSOS: UM OLHAR CRITERIOSO DA ACUPUNTURA** *Rosângela Lodi Queiroz*

**RESUMO.** A acupuntura é um método de intervenção terapêutica que visa o equilíbrio do organismo por meio de várias técnicas. Com o aumento da expectativa de vida, agrega-se a possibilidade do aumento de doenças crônicas. A acupuntura aliada aos tratamentos da medicina ocidental favorece a diminuição de sintomas relacionados as doenças crônicas e auxilia na prevenção de possíveis agravos a saúde dos idosos. Sendo assim, levantou-se

o perfil de idosos atendidos em um ambulatório de acupuntura, situado na região central de São Paulo, para verificar quais as queixas principais que levaram esses idosos a buscar esse método de tratamento. Foram revisados 540 prontuários, sendo que destes apenas 21% eram de idosos, com idade média de 72 anos, 86% são do gênero feminino, sendo 34% solteiras, 36% casadas, 20% divorciadas, 31% viúvas. Os homens somam 14%, dos quais 81% são casados, 6% divorciados e 13% viúvos. Essa população utiliza em média 3,3 medicações, com maior prevalência na população feminina. Quanto as queixas observa-se tanto as mulheres (48%) quanto os homens (44%) estão relacionadas a patologias osteo-articulares. A segunda maior queixa das idosas são emocionais 25%; nos homens cardio-vasculares e emocionais (13%). Verificou-se também que 46% das idosas e 69% dos idosos continuam trabalhando. As mulheres 46% participam de atividades de lazer; 50% realizam alguma atividade física e 30% não realizam nenhum tipo de atividade. Os homens somam-se 31%, 50% e 55%, respectivamente. Conclui-se que esse método é utilizado por uma prevalência feminina; as queixas álgicas são igualmente relatadas em ambos os gêneros, mas são evidenciadas as questões emocionais na população feminina. O diagnóstico na Medicina Tradicional Chinesa faz-se por meio da observação criteriosa da queixa do indivíduo, a alimentação, relatos emocionais, relacionais e ambientais, somente assim é possível verificar os desequilíbrios e desarmonias que venham ocasionar os sinais e sintomas. A acupuntura apesar de ser um recurso acessível, que favorece o bem-estar, a auto-estima, uma melhor compreensão dos hábitos de vida dos indivíduos, bem como a readequação dos mesmos e diminuição dos sintomas relacionados as doenças crônicas, sendo assim um excelente auxiliar para a manutenção da qualidade de vida dos idosos ainda é pouco utilizada por essa população, principalmente a masculina.

### PSICOLOGIA/NEUROPSICOLOGIA

**A ASSOCIAÇÃO DE SINTOMAS DEPRESSIVOS E DOENÇA DE PARKINSON ATRAVÉS DE UM ESTUDO NEUROPSICOLÓGICO** *Liliane Cristina de Além-Mar e Silva; Vicente de Paulo Higino; Heitor Dias Antunes Pereira*

**RESUMO.** Demência e depressão são síndromes que podem agravar e trazer irreversíveis consequências na evolução do processo da Doença de Parkinson (DP). Diminuindo o potencial cognitivo da pessoa, elas têm influência sobre a qualidade de vida, aumento de despesas de tratamento e sobrecarga do cuidador. A depressão antecede os sintomas motores em cerca de 25% dos parkinsonianos, e há uma associação positiva entre depressão e subseqüente risco de DP. Fator de risco, ou quadro precursor, tal comorbidade indica notória rapidez evolutiva ao olhar neuropsicológico. **Objetivo:** Demonstrar, de forma concreta, a progressão degenerativa de habilidades cognitivas em um caso de pessoa com doença de Parkinson associada a sintomas depressivos. **Metodologia:** São apresentados resultados de três instrumentos utilizados em duas avaliações neuropsicológicas, com diagnóstico de doença de Parkinson, com intervalo de dois anos entre si. Avaliada, inicialmente, aos 63 anos, e posteriormente aos 65 anos de idade, a paciente passou pela aplicação da Bateria Cognitiva (CERAD), Escala MATTIS para avaliação de demência, e Escala Geriátrica de Depressão (GDS). **Resultados:** Em todos os instrumentos, a paciente indicou diminuição de escores. Na Bateria CERAD, os percentuais de perdas foram maiores em praxia construtiva (100%), memória de lista de palavras (100%), evocação das praxias (100%), reconhecimento de lista de palavras (66,6%) e fluência verbal (41,6%). A nomeação se manteve intacta, e a paciente não evocou, tardiamente, nenhuma informação, em ambas as avaliações. Na Escala MATTIS, as maiores perdas seguiram-se nos domínios Construção (100%), Iniciativa e Perseveração (76,2%), Memória (61,2%), Atenção (32,4%) e Conceituação (6,9%). Os sintomas depressivos aumentaram em cerca de 36,4%, segundo aplicação da GDS. **Discussão:** Afetando com maior rapidez, e em maiores proporções, as habilidades motoras da pessoa, a doença de Parkinson mostrou, neste caso estudado, importante degeneração de funcionamento executivo, e, até por consequência, de aspectos mnemônicos. Os sintomas depressivos aumentados segundo descrição de familiares são quantitativamente percebidos, e possivelmente, influem na diminuição das habilidades cognitivas. **Conclusão:** As degenerações motoras e cognitivas da pessoa com Parkinson são, possivelmente, potencializadas pela evolução dos seus sintomas depressivos. O diagnóstico preciso e precoce destes parece ser importante ferramenta de promoção de qualidade de vida do portador de DP.

**ASSOCIATION BETWEEN SELF-PERCEPTION OF HEALTH AND COGNITIVE PERFORMANCE IN THE ELDERLY** *Camila Rosa de Oliveira; Cristiane Silva Esteves; Amanda Fernandes; Valéria Gonzatti; Luciane Scheufler; Juliana Colomby Ortiz; Irenio Gomes Filho; Irani Iracema de Lima Argimon; Tatiana Quarti Irigaray*

**ABSTRACT.** Assessment of self-perception of health contributes to characterize health conditions of the elderly. However, self-perception of health can be influenced by several factors such as sociodemographic characteristics, presence of physical problems and functional capacity. The aim of this study was to investigate the association between self-perception of health and cognitive abilities of elderly. Study participants were 358 older people from the southern region of the country, aged 60 and 95 years (M=68.28, SD=6.63) and different levels of education (M=4.42 years of formal schooling, SD=2.86). The elderly responded to a sociodemographic questionnaire, the Mini Mental State Examination (MMSE), the Geriatric Depression Scale of 15 points (GDS-15), the subtests Word List, Copying of Figures and Verbal Naming of the Consortium to Establish a Registry for Alzheimer's Disease (CERAD), tasks of verbal fluency (FAS and Animals) and the Logical Memory subtest of the Wechsler Memory Scale (WMS). The self-perception of health was evaluated using a likert scale ranging from 1 to 5 points, with higher scores refer to positive self-perceptions of health. We used descriptive analysis and Pearson‘s correlation, considering results significant at p≤0.05. The statistical package was SPSS 17. There were significant positive and weak associations between self-perception of health and MMSE, Word List (immediate recall), Logical Memory (immediate recall), Copy Graphics, Verbal Naming and Verbal Fluency (FAS) scores. There were also significant negative and moderate correlations between GDS-15 score and self-perception of health. According to the results we found an association between self-perceived of health and cognitive abilities. A positive self-perception of health is associated with higher scores on verbal memory immediate recall, verbal naming, visuoconstructional skills and executive functions. As a continuation of the study, it is suggested to investigate the protective and risk effects of self-perception of health for development of cognitive decline in the elderly.

**ATUAÇÃO NA NEUROPSICOLOGIA NO AMBULATÓRIO DE NEUROLOGIA ADULTO** *Bruna Carraro Burkot; Carla Regina Boldrini; Ana Paula Sabatini de Mello Braga*

**RESUMO. Introdução:** A atuação da Neuropsicologia tem se tornado fundamental na rotina de um Hospital Geral, devido a importância da avaliação neuropsicológica (ANP) no diagnóstico, intervenção e tratamento de diferentes patologias. Na Irmandade da Santa Casa de Misericórdia de São Paulo (ISCMSP) o setor de Neuropsicologia foi criado no ano de 2004 e atualmente conta com três neuropsicólogas no Hospital Central que atuam nos seguintes ambulatórios da Neurologia Adulto: Distúrbios do Comportamento, Distúrbios do Movimento, Distúrbios do Sono, Epilepsia, Neuroinfectologia, Neuroimunologia, Neuromuscular e Neurovascular. **Objetivo:** Apresentar a atuação do Setor de Neuropsicologia com pacientes neurológicos adultos na ISCMSP. **Métodos:** Descrição da atuação da neuropsicologia através de ANP de pacientes com patologias neurológicas atendidos no Ambulatório de Neurologia. **Resultados:** São recebidos em média de 60 pedidos/mês e a maioria destas solicitações (cerca de 80%) é para diagnóstico diferencial, o restante são para auxiliar a eficácia do tratamento e evolução da doença. Em média, a ANP ocorre em quatro sessões, sendo que no primeiro atendimento é realizada anamnese detalhada com o paciente e acompanhante (se houver). O processo avaliativo consiste baterias semi-flexíveis, com testes que avaliam as seguintes funções: recursos intelectuais, atenção, memória, linguagem, praxia, gnosia, funções executivas e aspectos emocionais e comportamentais. A última sessão é a devolutiva, na qual o relatório da ANP é detalhadamente explicado e também são dadas orientações/encaminhamentos, visando maior funcionalidade e qualidade de vida. A atuação no neuropsicólogo no Hospital Geral incide fundamentalmente no processo da ANP, porém também inclui a discussão com equipe multidisciplinar, condução de palestras e grupos. **Conclusão:** A atuação do neuropsicólogo nos Ambulatórios da Neurologia Adulto auxilia a equipe médica a nortear o diagnóstico, acompanhamento e tratamento. Caracteriza o único modo de avaliar quantitativa e qualitativamente as potencialidades e dificuldades cognitivas dos pacientes. Também é relevante para identificar demandas, orientar para uma melhor qualidade de vida, encaminhar para tratamentos necessários.

**AVALIAÇÃO COGNITIVA BREVE DE IDOSOS ATENDIDOS EM AMBULATÓRIO GERONTOLÓGICO GERAL** *Luciana Cassimiro; Bruna Cristina da Silva Manso*

**RESUMO. Introdução:** A avaliação das funções cognitivas é habitual na avaliação dos processos demenciais, porém ainda não faz parte dos protocolos gerais de atendimento da maioria dos ambulatórios gerontológicos. **Objetivo:** Avaliar um grupo de indivíduos atendidos em ambulatório gerontológico, para analisar a relevância da realização de exames sistemáticos das funções cognitivas em indivíduos sem queixas desta natureza. **Métodos:** Foram incluídos neste estudo, 45 idosos voluntários, atendidos no Instituto Paulista de Geriatria e Gerontologia - José Ermínio de Moraes (IPGG-JEM) no período de Fevereiro a Maio de 2013, sem queixas de alterações cognitivas. Os pacientes foram submetidos aos seguintes testes cognitivos: MEEM, extensão de dígitos, testes de memória de figuras, fluência verbal, teste do desenho do relógio. **Resultados:** Aproximadamente 1/3 dos pacientes apresentaram desempenho comprometido em pelo menos um dos testes aplicados. O MEEM mostrou-se alterado em 12% amostra, o teste de extensão de dígitos demonstrou alteração em 15% dos pacientes. A evocação tardia de figuras mostrou alteração em 11,5% dos casos, a fluência verbal apresentou alteração em 10% dos idosos e o desenho do relógio em 30%. **Conclusão:** os dados obtidos comprovam a necessidade da inclusão da avaliação cognitiva breve como importante instrumento no atendimento cotidiano de um ambulatório gerontológico, para melhoria na qualidade do atendimento e tratamento destes idosos.

**COGNITIVE EVOLUTION OF ELDERLY WITH ATTENTION DEFICIT HYPERACTIVITY DISORDER (ADHD) - 2-YEARS FOLLOW-UP OF THREE CASES** *Margarete Klein; Maria Aparecida da Silva; Mário Rodrigues Louzã Neto*

**ABSTRACT.** Difficulties associated with executive functions, attention and memory are common in young adults with ADHD; these are the same functions involved in the cognitive decline of aging. To our knowledge there are no studies about cognitive functioning in the elderly population with ADHD. **Objective:** To analyze the cognitive evolution in two years of 3 subjects aged over 60 years, diagnosed with ADHD. **Methods:** Three subjects aged 63(Case 1), 65(Case 2) and 79 years (Case 3) with ADHD according to DSM-IV criteria. Three neuropsychological assessments were conducted with intervals of 1 year. They were evaluated without psychostimulants. Instruments: Digits-WAIS (short-term memory/working), Trail Making Test -TMT (attentional functions), Stroop Test (inhibitory control), Rey Auditory Verbal Learning Test-RAVLT (episodic memory); Brief Visual Memory Test-BVMT(episodic memory), Boston Naming (language), Verbal Fluency, Wisconsin (cognitive flexibility),Cubes-WAIS (visuospatial) and Questionnaire on Cognitive Decline in the Elderly ( IQCODE - BR/short version). **Results:** Results with more than 1.5 standard deviation below norm were: Case 1: Stroop I (baseline: -1,8/ one year: -14,0/ two years: -2,1), Stroop II (-5,1/-2,8/-2,0), Stroop III (-2,2/-1,7/0,5). Case 2: TMT A (-1,8/-2,0/-3,2), TMT B (-1,6/-1,0/-0,2); Stroop I (-1,8/-5,5/-5,5), Stroop II (-0,2/-2,8/-2,8), BVMT immediate recall ( -1,0/-2,8/-1,4). Case 3: TMT A (-3,4/-0,9/-0,5), TMT B (-1,3/-1,0/-9,5), Stroop I (-0,9/-0,9/-3,9), Stroop III (-3,8/-2,3/0,1), RAVLT late recall (-5,8/-5,8/-5,8), BVMT immediate recall (-2,8/-2,1/-1,1), Wisconsin completed categories (-1,6/-1,6/-1,0). Language, verbal fluency, working memory, visuospatial, verbal and visual recognition were within normal limits in three cases. IQCODE (cutoff:4.0/4.1) Case 1: 2.81/Case 2: 3.81/Case 3: 3.35 **Conclusion:** The subjects show cognitive deficits consistent with ADHD. Their performances along time show fluctuations sometimes with improvement from baseline to follow up. No clear cognitive decline during these 2 years was observed, so that their impairments seem to be related only to ADHD.

**CONSCIÊNCIA DA DOENÇA NA DEMÊNCIA: DIFERENÇAS ENTRE DOMÍNIOS** *Marcia Dourado; Maria Fernanda Barroso de Sousa; Daniel Mograbi; Raquel Luiza Santos; Tatiana Belfort; Raquel Dias; Bianca Torres Mendonça de Melo*

**RESUMO. Introdução:** Consciência da doença é a capacidade de reconhecimento de sintomas e alterações causados pela demência. Estudos sugerem que a consciência da doença é um fenômeno constituído por domínios que podem ser considerados independentes como a consciência do déficit cognitivo e o reconhecimento das alterações funcionais. **Objetivo:** Avaliar a presença de diferentes domínios da consciência da doença em pessoas com Doença de Alzheimer. **Método:** Foram avaliados a consciência da doença, estado cognitivo, funcional, estadiamento clínico da doença, depressão, sobrecarga e qualidade de vida de 208 pessoas com doença de Alzheimer leve e moderada e seus cuidadores, recrutados no Centro para Doença de Alzheimer do Instituto de Psiquiatria da UFRJ. **Resultados:** Idade (75.6 (7.3) / 58-93), gênero (f=130 / m=71), MEEM (20.3 (3.8) / 12-27), Pfeffer

(16.4 (8.5) / 0-30), Cornell (4.4 (3.3) / 0-19), AIPDD (7.5 (5.7) / 0-25), QDV (34.1 (6.3) / 15-52). A análise sugere a presença de um domínio predominante - consciência do déficit cognitivo (eigenvalue=1.99, responsável por 49.8% da variância) e 3 outros domínios menores - consciência do déficit funcional, próprio estado emocional e alterações relacionais (eigenvalues =.83, .68 e .49, respectivamente). **Conclusão:** A consciência da doença é um fenômeno multidimensional, pois o fato da capacidade de reconhecimento estar comprometida em um domínio, não implica em comprometimento em outro domínio diferente.

**EMOTIONAL IMPACT ON THE ELDERLY PSYCHIATRIC PATIENT BEFORE A FALL: A CASE REPORT** *Eliana de Souza Cardoso; Denise Regina Piva; Isabel Alonso Leite; Mariana Haron Brandão; Natália Cristina Moraes*

**ABSTRACT.** According to the World Health Organization (WHO, 2010), approximately 28% to 35% of people over age 65 suffer falls, increasing with age and level of frailty. Fall is an event of unexpected nature and depending on its severity can trigger a number of limitations, such as loss of independence, social limitation, emotional disorders, insecurity and fear of falling again. From the standpoint of psychological / emotional, one can understand the fall as a traumatic event that can generate disruptions on account of disability, depending on the impact, reflecting the life of the person **Objective:** This objective study is describe a clinical case treated by the Division of Psychology of the Reference Center for the Elderly of the Northern Zone of São Paulo (CRI-North), outpatient secondary care the Unified Health System (SUS), trying to demonstrate the emotional impact that the decline may represent the life of the elderly. **Methods:** We carried out about ten calls based on brief psychotherapy individual with psychoanalytic theoretical framework, objective with woman patient, 62 years old, married, a daily laborer and caretaker, who kept active life after the fall in which fractured fists, was referred for psychological treatment because of changes humor and anxiety and depression symptoms. **Results:** During treatment the patient presented aspects of life history and position as a family constitution to be caring for a large part of life, such reports were presented in a confusing and decoupled. Did not recognize the place it can be taken care of, with difficulties to realize their own limitations and body perception. It can be seen that the fall was also a triggering event for the patient to get in touch with aging, in this way experiencing the relationship between caring and being cared for. **Conclusion:** One can understand the fall as a trauma and loss of control of your own body and the functions performed by it, generating psychic disorganization. Psychotherapy use some question to formulate such questions, it is possible that the patient was in contact with the suffering caused by this trauma thereby promoting insight in order to be cared for and live in a more peaceful this other way of being in the world. Such issues were possible to be treated as they realized the subjectivity experienced by her and their particular experiences. Keywords: elderly, falls, psychotherapy, psychiatric

**MAUS-TRATOS E COMPROMETIMENTO COGNITIVO EM IDOSOS** *Cristiane Silva Esteves; Luisa Steiger Pires de Oliveira; Tatiana Quarti Irigaray; Camila Rosa de Oliveira; Irani Iracema de Lima Argimon*

**RESUMO.** Idosos que possuem declínio cognitivo são mais susceptíveis a desenvolver demência, o que os torna mais vulneráveis à exposição a maus-tratos. É sabido que a violência contra o idoso afeta sua qualidade de vida, ocasionando diversos transtornos. O presente estudo objetivou identificar associação entre maustratos e declínio cognitivo em idosos atendidos pela Estratégia Saúde da Família (ESF) do município de Porto Alegre. O delineamento foi de um estudo transversal, descritivo, coletado de forma prospectiva em uma amostra composta por 251 idosos de 60 anos ou mais, divididos em grupo que sofreu maus tratos (N=138) e grupo controle (N=113). Esta pesquisa foi realizada a partir de um questionário composto por 10 questões, elaborado a partir da definição de Minayo, que classifica os tipos de maus-tratos em abuso físico, psicológico, sexual e financeiro e em negligência. Após assinatura do Termo de Consentimento, os idosos responderam a uma ficha de dados sociodemográficos, à Escala de Depressão Geriátrica de 15 pontos (GDS-15), ao Mini Exame do Estado Mental (MEEM), ao subteste Lista de Palavras do Consortium to Establish a Registry for Alzheimer's Diseasea (CERAD), Teste Boston versão reduzida, tarefas de fluência verbal (FAS e Animais) e ao subteste Memória Lógica da Escala Wechsler de Memória (WMS-L). A análise de dados ocorreu através do Teste t de Student, considerando-se resultados significativos quando p≤0,05. Os grupos não apresentaram diferenças estatisticamente significativas em relação à idade e à escolaridade. O grupo com histórico de maus-tratos foi composto por 97 (70%) mulheres e 41 (30%) homens, e no grupo controle, foi de 61 (54%) e 46 (46%), respectivamente. O grupo com maus-tratos obteve pior resultado em todos os instrumentos, entretanto, os grupos tiveram diferenças significativas nos resultados dos seguintes testes: MEEM, Boston, FAS e WMS-L (imediata). De acordo com os resultados, os idosos que sofreram maus-tratos, em comparação aos idosos do grupo controle, apresentaram um pior desempenho no funcionamento cognitivo geral e um maior número de sintomas depressivos. Ainda, obtiveram menores escores em nomeação verbal, em fluência verbal e em memória verbal episódico-semântica de evocação imediata. A identificação precoce de idosos com declínio cognitivo pode favorecer a implementação de ações de prevenção a maus-tratos contra idosos.

**MOTIVATIONAL ASPECTS THAT LEAD TO ADHERENCE TO WORKSHOPS OF COGNITIVE STIMULATION** *Cristiane Nogueira*

**ABSTRACT.** Cognitive Stimulation Workshops are a theme in expansion in Brazil, but little is said about Workshops for people not enrolled and what motivational aspects leads them to engage in the project. Goals: To analyze the main motivational aspects that drive normal elderly to seek the services of stimulation as well as their expectations for service. **Methods:** This is a quantiqualitative social research, using the method of action research. The sample consisted of 15 elderly patients age over 60, with 1 to 5 years of schooling who had attended two classes on health (including topics such as Alzheimer's, Vascular Dementia and Depression) and attended assiduously cognitive stimulation group for one year. The classes were held in a citizen club in a city in the state of São Paulo. These participants underwent interviews and informal conversations that point to the understanding of the subject in its action (DESLANDES, 1994). This enabled the capture of repressed or not easily articulated psychological data such as attitudes, motives or assumptions, which needs the participation of the researcher to bring forth the reality of the subject. **Results:** The elderly studied were on average 70.5 years-old, 99% female, currently housewife, married or widowed, retired, and average income of two minimum wages. After done the research and observation of the group for a period of one year, which was verified their initial motivation was the concern with neurodegenerative diseases, since most had a family history, but over the course of the activities, the initial motivation became pleasure of learning, by moments of entertainment with positive thoughts in relation to cognitive, attributing cognitive improvement in the participation of the project. During this period was also noticeable awareness of a new lifestyle, trying to live what OMS defines health as: a state of complete well-being-physical, mental and social wellbeing and not merely absence of disease and illnesses. **Conclusion:** There are many noticeable gains compared to regular participation in the project, so that was surpassed initial expectations regarding the method, then, what was to answer a priori cognitive demand, such as improving memory, language, attention, concentration, spatial skills, among other functions, started to benefit other aspects such as psychological and social.

**PERCEPÇÃO DA SOBRECARGA EM CUIDADORES INFORMAIS DE IDOSOS DEPENDENTES COM DEMÊNCIA** *Iracelia Munhoz Moreira; Renata Firpo R. Medeiros; Mércia Gomes Rodrigues; Audrey Andrade Bertolini; Ana Lúcia Alves; Gisele Monaco Dias*

**RESUMO.** A sobrecarga do cuidador é definida como o conjunto de problemas físicos, psicológicos, emocionais, sociais e financeiros experimentados por aqueles que cuidam de pacientes com algum tipo de comprometimento. Normalmente, o cuidador deve responsabilizar-se pela rede de cuidados necessários ao sujeito. No entanto, é comum o desconhecimento sobre como lidar adequadamente com o idoso com demência, surgindo a necessidade de orientação e suporte. Portanto, o cuidador também se torna foco de cuidado, recebendo cada vez mais atenção dos profissionais e serviços de saúde. **Objetivo:** Analisar a percepção da sobrecarga em cuidadores de idosos dependentes com demência residentes em ILPI. **Métodos:** Trata-se de um estudo transversal, foi aplicado questionário contendo questões relacionadas de como o cuidador se sente em relação ao idoso cuidado, respondendo: nunca, raramente, algumas vezes, frequentemente e sempre. Os questionários foram aplicadoanonimamente. **Resultados:** A amostra foi constituída de 33 cuidadores de idosos informais, do sexo feminino, que prestam seus serviços em uma ILPI. Quando questionados se sentem que o idoso pede mais ajuda do que necessita, 39,39% responderam algumas vezes e 21,90% sempre; se não tem tempo suficiente para si mesmo 30,30% raramente, 24,24% algumas vezes; se sentem estressados 15,15% raramente e 18,18% algumas vezes; se sente envergonhado pelo comportamento do idoso, 15,15% raramente; se sente irritado com o idoso 93,3% referiram nunca, incapaz de cuidar do idosos por muito mais tempo 87,77% responderam nunca; se gostaria que outra pessoa cuidasse do idoso 36,36% algumas vezes; se sente dúvida como cuidar do idoso 39,39% algumas vezes; sente que poderia fazer mais 24,24% algumas vezes e 18,18% sempre; se poderia cuidar melhor do idoso 24,24% sempre; se sente sobrecarregado 39,39% frequentemente e sempre; sente medo de envelhecer e ficar dependente 45,45% algumas vezes e 18,18% sempre. **Conclusão:** Estes resultados demonstram a necessidade dos cuidadores, também serem cuidados, já que constituem um componente fundamental nos cuidados de saúde ao idoso dependente. Sem a atenção e o apoio necessário e adequado, há o risco dos cuidadores se tornarem também pacientes. Aliás, quando são prestados serviços de apoio formal adequados às necessidades dos cuidadores informais, estes persistem como os parceiros-chave no sistema de apoio ao idoso dependente.

**PERFIL DE VISITAS E PERIODICIDADE AOS IDOSOS DEMENCIADOS RESIDENTES EM ILPI** *Iracelia Munhoz Moreira; Mércia gomes Rodrigues; Gisele Monaco Dias; Renata Firpo R. Medeiros; Audrey Andrade Bertolini; Ana Lúcia Alves*

**RESUMO. Introdução:** A família é parte integrante no processo de envelhecimento, visto que pode desenvolver e manter o equilíbrio físico e afetivo do idoso. Podemos afirmar que a expectativa da instituição, ainda que não sejam expressas tão claramente nem implementadas com a necessária rapidezvisam estratégias que avancem neste sentido. Por outro lado, é preciso lembrar que a família, é quem busca a instituição de longa permanência como parceira nas demandas de cuidado. Ao acoplar-se à instituição, a família busca a extensão de si mesma para cuidar adequadamente seu familiar. Há diversificada relação entre o idoso e a família tan-to entre aqueles que mantêm (ou retomam) relacionamentos significativos com seus familiares, baseados nos fortes laços familiares, como naqueles que, por circunstâncias diversas, romperam os vínculos, ou mantêm uma comunicação ruidosa.O afastamento prolongado da família ocasiona, depressão, angustia e solidão no idoso, que se sente abandonado. A instituição, deve proporcionar e motivar a integração da família com o idoso dentro da instituição, mostrar para a família a importância das visitas periódicas aos idosos; sua participação em eventos, como festas temáticas, aniversários, atividades de lazer; sendo importantes para o bem estar dos idosos, e resgatar os vínculos familiares. **Objetivo:** Avaliar

o grau de relacionamento do visitador e aperiodicidade das visitasaos idosos com grande dependência de uma Instituição de Longa Permanência (ILP). **Métodos:** Os dados foram coletados dosregistros de visitas aos idosos na portaria da ILP durante o período de fevereiro de 2011 a fevereiro de 2012. **Resultados:** Foram avaliadas as visitas recebidas por 42 idosos com idade média de 86,33±8,34 anos. Sendoque: 52,39% semanalmente, 28,57% quinzenalmente, 11,90% mensalmente, 2,38% somente em eventos festivos e 4,76% não receberam nehuma visita no periodo de um ano. Visita de filhos foram 66,66%, seguida por sobrinhos 19,04%, netos 16,66%, amigos 11,90%, cônjuge e irmãos 4,76% emãe 2,38%. **Conclusão:** O estudo mostrou que os filhos representam um vínculo importante com o idoso institucionalizado, corroborando com os dados de literaturaspesquisadas. AILP não pode substituir a família mas, motivar e manter os vínculos familiares na vida do idoso institucionalizado.

**QUALIDADE DE VIDA NA DEMÊNCIA: O PAPEL DOS FATORES NÃO COGNITIVOS NAS DIFERENÇAS ENTRE A PERCEPÇÃO DO PACIENTE DE SEU CUIDADOR** *Marcia Cristina Nascimento Dourado; Maria Fernanda Barro de Sousa; Raquel Luiza Santos; José Pedro Simões; Tatiana Belfort; Bianca Torres Mendonça de Melo; Rachel Dias*

**RESUMO. Introdução:** A validade do relato do paciente com demência sobre sua própria qualidade de vida (QdV) é alvo de controvérsias. **Objetivo:** Avaliar os fatores não cognitivos relacionados à percepção do paciente sobre sua QdV e a percepção do cuidador sobre a qualidade de vida do paciente. **Método:** Em um estudo transversaI, 41 pacientes com Doença de Alzheimer leve e seus cuidadores foram recrutados, consecutivamente. Foram avaliadas a qualidade de vida (paciente e cuidador), consciência da doença, cognição, presença de sintomas depressivos, funcionalidade, estadiamento clínico da demência e a sobrecarga do cuidador. **Resultados:** Foram observadas diferenças significativas entre os escores de pacientes e cuidadores sobre a QDV dos pacientes (t=3.292, p<0.01, d=0.727). A regressão linear indicou que o comprometimento da consciência da doença estava relacionado à percepção positiva do paciente sobre sua QDV (p=0,001). O grau de escolaridade do cuidador (p=0,000) e a presença de sintomas depressivos dos pacientes (p=0,000) foram os fatores relacionados à percepção negativa dos cuidadores sobre a qualidade de vida dos pacientes. **Conclusão:** O comprometimento cognitivo não foi o principal fator relacionado à diferença entre os relatos do paciente do paciente e seu cuidador sobre a QdV do paciente. Nossos achados sugerem que os fatores não cognitivos como consciência da doença e presença de sintomas depressivos estão relacionados às diferenças de percepção no estágio inicial da demência do paciente e seu cuidador sobre a QdV do paciente. Ao considerar somente fatores cognitivos ou funcionais, pode-se negligenciar importantes fatores que influenciam a qualidade de vida de pacientes e seus cuidadores.

**REVITALIZAR - VIDA E TRABALHO, INTEGRAÇÃO PARA MELHOR CUIDADO NO IDOSO DEMENCIADO** *Iracelia Munhoz Moreira; Ana Lúcia Alves; Audrey Andrade Bertolini; Gisele Monaco Dias; Renata Firpo R. Medeiros; Mércia gomes Rodrigues*

**RESUMO.** O movimento na direção de construir, conceitualmente e na prática concreta de serviços, o trabalho em equipe, tem sido um esforço visando o maior e melhor cuidar na saúde. O trabalho em equipe pode se desdobrar em valorações hierárquicas e desigualdades sociais. Entendemos que o cuidar do idoso vai além de um trabalho técnico hierarquizado, para um trabalho com interação social entre os trabalhadores, com maior horizontalidade e flexibilidade, possibilitando maior autonomia e criatividade visando maior integração da equipe. Se está integração não ocorrer corremos o risco de repetir o modelo de atenção desumanizado e fragmentado. **Objetivo:** Implantação de um programa de integração entre os funcionários de uma ILPI, estimulando o relacionamento interpessoal, o trabalho em equipe e o respeito mútuo, visando maior envolvimento e cuidado com o idoso. **Métodos:** Os funcionários foram convidados a participar do projeto de forma voluntária, foram divididos em dois grupos para melhor acomodação, levados a um sítio, fora do ambiente de trabalho. Foram aplicadas atividades atividadesque envolviam os funcionários fisicamente, emocionalmente e socialmente; demonstrando através dessas a importância do seu companheiro e que quando um colabora o trabalho fica mais fácil e valorizado. Todas as atividades propostas respeitavam os limites e diferenças de cada um. O evento foi supervisionado por fisioterapeuta e psicóloga. **Resultados/Conclusão:** Participaram do projeto 54 funcionárias, do sexo feminino, de todos os setores de uma ILPI, enfermagem, limpeza, copa, cozinha e lavandeira. Através de atividades que envolviam todos os membros da equipe, podemos demonstrar que a competência de cada profissional, isoladamente não dá conta da complexidade do atendimento e das necessidades do idoso, portanto é necessário flexibilidade nos limites das competências, arguir a desigualdade na valoração dos distintos trabalhos para proporcionar um cuidar integral. Podemos notar também maior entrosamento pessoal, motivação e responsabilidade nas suas atividades laborativas.

**THE IMPORTANCE OF THE NEUROPSYCHOLOGICAL DIAGNOSIS OF ALZHEIMER'S DISEASE** *Eliana de Souza Cardoso; Denise Regina Piva; Isabel Alonso Leite; Mariana Haron Brandão; Natália Cristina Moraes*

**ABSTRACT.** Alzheimer's disease (AD) is the most common neurodegenerative disease associated with aging, cognitive and neuropsychiatric manifestations of which result in progressive disease and disability. Affects approximately 10% of individuals aged 65 years and 40% over 80 years. The initial symptom of the disease is characterized by progressive loss of recent memory, with the evolution of the disease, other changes occur in memory and cognition as language and visuospatial functions. These symptoms are often accompanied by behavioral problems, including aggression, depression and hallucinations. The Neuropsychological Assessment is a useful tool in the overall assessment of elderly patients, since it gives subsidies of cognitive areas that are affected by battery of specific tests. **Objective:** To describe the neuropsychological assessment service held at the Department of Psychology Reference Center for the Elderly of the Northern Zone of São Paulo (CRI-North), outpatient secondary care the Unified Health System (SUS), seeking to demonstrate the importance of evaluation Neuropsychological and emotional impact that insanity represents the life of the elderly and caregivers. **Methods:** Using neuropsychological instruments for comprehensive evaluation of elderly patients with specific batteries and sensitive assessment of cognitive functions such as attention, memory, language, executive function. Also includes detailed history, collects complaints provided by the patient and / or family, tests and devolution of specific data. **Results:** Behavioral and psychological symptoms (insomnia, psychomotor agitation and hyper sexuality) are observed at different stages of AD. Early onset of AD may be confounded with psychiatric disorders. The impact on the lives of the patient and family is intense and treatment in the early stages is of paramount importance to reduce the burden on caregivers and patients. **Conclusion:** The cognitive and neuropsychiatric changes observed provoke an emotional impact on the lives of the elderly and their careers, and can interfere with quality of life to the extent that such symptoms are not treated. Neuropsychology has much to contribute to the treatment and diagnostic use of dementia syndromes, as it assists the subsequent definition of therapeutics and medicines. This requires the extension and further validation of tests for elderly public. Keywords: Elderly, Neuropsychological Assessment of Alzheimer's Disease

### PSIQUIATRIA

**COGNITIVE-BEHAVIORAL THERAPY IN DEMENTIA: LITERATURE REVIEW** *Gilson de Oliveira Carol Júnior; Fabia Maria de Lima; Edvan Lima; Kátia Cristina Lima de Petribú*

**ABSTRACT.** The growth of the elderly population in Brazil has resulted in a significant increase in the number of people with dementia. The true prevalence of this disease needs to be investigated in order to establish appropriate measures to improve the quality of life of those affected. **Objective:** The aim of this study was to prepare a literature review focusing on dementia and cognitive-behavioral therapy, based on the production of scientific papers. **Methods:** Performed research in the database for articles, consulted as sources of references was obtained from the literature of library materials indexed in the Virtual Health in Brazil, using specific keywords DESC - CBT (cognitive therapy, behavioral therapy and cognitive behavioral therapy) and dementia (vascular, Alzheimer, Parkinson, bodies of Lewis). We sought to employ a time frame for the last twelve years, from 2000 to 2012. **Results and Discussion:** A total of 9 articles were identified through the literature search and the majority of materials indexed in English. Of this total, only 7 met the inclusion criterial descriptors and two were excluded two. All somehow found favorable results with CBT elderly with dementia. However it is observed that CBT was more useful for elderly patients with diagnosis of mild dementia associated with anxiety or depression. **Conclusion:** Despite the lack of studies and methodological limitations observed that there was a positive response to treatment with CBT when dementia is associated with symptoms of anxiety or depression. Although randomized trials are needed.

**CREATIVITY AS A THERAPEUTIC RESOURCE IN AGING** *Maria Cristina Reis Amendoeira*

**ABSTRACT.** Approach through the images produced by the Museum of Images of the Unconscious that maintained and improved its power of creation through old age, developing aspects relating to aging and death in artistic expression. This communication originates from a doctoral research on the ability of expression through images in people with mental disorder. One of the stages of research, developed at the Federal University of Rio de Janeiro and Museum of Images of the Unconscious - Nise da Silveira, discusses the permanence of the creative ability in schizophrenia patients of the paint Studio of the Museum of Images of the Unconscious, after 60 years, through a pictorial sample of their production after more than 40 years of psychiatric hospitalization. The passage of time for these patients brings changes in communication skills that accompany aging and mental illness and also commit the autonomy, the self-esteem, the identity and quality of life of them. The expressive activity can be a creative outlet in aging. The pictures, in their expressive values are process source of affection and meanings. In particular the artistic image has an inventiveness clearly higher than any other image: it remains in the sphere of invention and of the discovery. The field of art is the ground of the sensitivity and proves to be more democratic in terms of possibilities. The limitations can open new poetic possibilities. This is the field where the limitation can be overcome or subverted. Key words: Creativity and Ageing; Museum of Images of Unconscious and Ageing; Art and Ageing.

**PREVALENCE OF GENERALIZED ANXIETY DISORDER AND POST TRAUMATIC STRESS DISORDER IN THE ELDERLY** *Elisa Fasolin Mello; Caroline Picoli Menta; Kenia Fogaca Silveira; Alfredo Cataldo Neto; Irenio Gomes da Silva Filho; Eduardo Lopes Nogueira; Francisco Pascoal Jr.*

**ABSTRACT.** Due to demographic shifts and inadequate management, geriatric anxiety disorders will become and increasing human and economic burden. Anxiety disorders are common in older adults and cause considerable distress and functional impairment. Generalized Anxiety Disorder (GAD) is a common mental disorder that typically has an early age of onset, a chronic course, and a high degree of comorbidity with other anxiety and mood disorders. The post traumatic stress disorder (PTSD) is a disease that is increasing its incidence, because the steady growth of violence today, notes that its development and its association with other diseases, leads to increased morbidity and mortality, as the association with depression and suicide risk, which also becomes a factor in the severity of health. Aims: This study will describe the prevalence of generalized anxiety disorder and post traumatic stress disorder in a Porto Alegre's community- dwelling population aged 65 years and older. Also, to correlate association between GAD and PTSD with demographic description. **Methods:** A prospective cross-sectional study was conducted. The sample consisted of 587 randomly selected older people from 27 Family Health Teams in the city of Porto Alegre (FHT/POA) drawn in a stratified manner by the “Health Districs”. Diagnosis was made by psychiatrists experienced in evaluating the elderly and using the Brazilian version of the Mini International Neuropsychiatric Interview (M.I.N.I 5.0.0 plus). **Results:** Of the 587 individual 376 (64%) were women and 211 (36%) men, being 341 (58%) aged 60 to 69 years, 160 (27%) aged 70-79 years and 48 (8%) aged 80 years or older. Of these 52 elderly (17%) had GAD and 20 older adults (6%) PTSD. And 9 elderly (17.3%) had associated the two disorders. Analyzing the data, it was observed that both the GAD as PTSD are more prevalent in women, and the elderly GAD found in 40 (11%) and 12 older adults (6%) (p 0.04) and PTSD in 15 elderly (4 %) and 5 older (2%) (p 0, 29). It was observed that the higher incidence of these disorders occurs in the age group of 60-69 years 37 elderly (11%) and GAD 16 elderly (5%) in PTSD. **Conclusion:** Compared with previous studies of prevalence, these disorders is increasing its incidence in the elderly population, which consequently leads to a poor quality of life and a greater risk of triggering other psychiatric disorders such as major depressive disorder.

**THE PSYCHOANALYTIC TREATMENT: POSSIBILITIES AND CONSTRUCTION OF SUBJECTIVITY IN AGING** *Maria Cristina Reis Amendoeira*

**ABSTRACT.** The elderly patient communications in psychoanalytic treatment indicate the possibility of developing psychic conflicts in old age. The subjective experience of aging is addressed from Freud through his correspondence and text and also from the intellectual and personal experience of some contemporary authors as Norbeto Bobbio, Norbert Elias, in addition to the communications of the patients of advanced age. The subjective aspects that interfere with the normal or pathological aging are valued in the understanding and treatment of old age in the interdisciplinary field of mental health. The clinical psychoanalytic treatment reaches, the rescue of subjectivity and the use of creativity as a therapeutic resource are some of the themes developed by contemporary psychoanalytic authors. The technical aspects such as maintenance or changes in the setting, the quality of listening as well as manifestations of the transference and of the counter transference overflowing in relationship with the elderly are current topics of the psychoanalytic clinic. The stigma of death and dying act are taboos in our society and contain in itself the basic contradiction of our time: the emotional distance between people has become larger, although the need of another and of their affection remains intense and urgent. In aging there is suffering from real and illusory losses of all existence. The predominance of loneliness and feelings of not having reason to keep alive correlate it selves with suicide - the relations between age and suicidal behavior are strong. The psychoanalytic treatment assists the patient in the construction of subjectivity to better understand the impact of the experience of the passage of time and the closeness of the death. Key words: Psychoanalytic treatment and ageing; Subjectivity and Ageing.

**USO DE ÁLCOOL EM IDOSOS. ESTUDO DE BASE POPULACIONAL NO MUNICÍPIO DE FLORIANÓPOLIS** *Marcos Antonio Lopes; André Junqueira Xavier; Eleonora D‘Orsi*

**RESUMO.** Introdução. O uso de álcool em idosos é muito pouco estudado no Brasil e no mundo. Os dados existentes apontam uma prevalência de uso problemático comparável à dos adultos. Objetivo. O presente estudo tem o objetivo de investigar o uso de álcool em uma amostra de idosos da comunidade, no município de Florianópolis. Este estudo pertence ao levantamento epidemiológico da saúde do idoso, denominado EPIFLORIPA. Métodos. A população examinada foi um amostra representativa dos idosos com idade maior ou igual a 60 anos. Os instrumentos utilizados foram o Alcohol Use Disorders Identification Test (AUDIT; as três questões referentes a quantidade e frequência de uso de álcool), o Mini Mental State Examination (MMSE), para avaliação cognitiva, o Brazilian Older Americans Resources and Services Multidimensional Functional Assessment Questionnaire (B-OARS-MFAQ), para avaliação funcional, e um questionário clínico. Resultados. A amostra consistiu de 1.705 idosos, com uma média de idade de 70,6 anos (60-104; DP: 8.0) e predominantemente casada; 63,9% eram do sexo feminino e 55,6% tinham pelo menos 5 anos de escolaridade. Uso de álcool esteve presente em 35% dos idosos (N=596) e foi associado a idade mais jovem, sexo masculino e níveis mais altos de escolaridade e renda (p >0,001). Uso muito frequente (4 ou mais vezes por semana) e uso de risco (7 ou mais doses por semana) esteve presente em 7,2% (N=123) e 6,5% (N=111) dos idosos, respectivamente. Uso de baixo risco e uso pouco frequente foram associados a menores taxas de comprometimento cognitivo e funcional, quando comparados aos abstinentes (ajustados para idade; p >0,05). Conclusões. O uso de álcool em idosos na amostra estudada foi semelhante ao da população adulta brasileira. O uso com potencial para causar problemas foi bastante prevalente e deveria demandar atenção por parte das ações de saúde entre os idosos.

**VARIABLES ASSOCIATED TO PROFESSIONAL CAREGIVERS ADHESION TO A PSYCHOEDUCATIONAL TRAINING FOR MANAGEMENT OF BEHAVIORAL AND PSYCHOLOGICAL SYMPTOMS OF DEMENTIA IN NURSING HOME RESIDENTS** *Débora Dalpai; Ramon Castro Reis; Marianne Le Bourlegat; Analuiza Camozzato*

**ABSTRACT.** Behavioral and psychological symptoms of dementia (BPSD) are very frequent in nursing home residents and more effective treatment for these symptoms are necessary. The psychoeducational training of the staff from nursing homes seems to be a promising way to reducing BPSD. The Staff Training for Assisted Living Residences (STAR) originally developed by Tery and colleagues, showed effectiveness to reduce these symptoms. There is an ongoing Brazilian multicentre trial aiming to evaluate the effectiveness of this training in Brazilian nursing homes. The STAR procedures were translated to Portuguese and this training has 12 modules of workshops plus 4 sessions of individualized training for staff. The aim of the present study is to evaluate the variables associated to professional caregivers adhesion to the training in the nursing homes from Porto Alegre. Until now, the training was implemented to twenty staff members from two nursing homes already. We categorized them, for the purpose of the present study, in staff member with full adhesion (staff members who participated of all training sessions) (N=9) and partial adhesion (staff members with 50 to 75% of frequency in the program) (N=11). Age, educational level, socioeconomic status, years working as a professional caregiver in nursing homes, years as employer in the current institution, Beck Depression Inventory, Beck Anxiety Inventory, Zarit Burden Interview and Dementia Management Strategies Scale (DMSS) scores were evaluated as independent variables for the outcome training adhesion in bivariate analyses with Student t test. The groups with full and partial adhesion only were significantly different in the Active Management DMSS factor (t=2,934 p=0,018). Active Management DMSS scale includes activities to safeguard, assist, engage, stimulate, and monitor, and associated behaviors primarily directed toward modifying the environment or daily routine. Staff members with partial adhesion to training presented higher active management scores as dementia management strategy (36,75±4,575) (mean±SD) than those staff members who had full adhesion to training (30,20±4,025) (mean±SD of active managament DMSS factor score). These preliminary results suggest that staff members who rated themselves as professionals with active management to deal with nursing home residents with dementia may feel that they already have sufficient expertise to deal with these patients, presenting lower adhesion to an structured psychoeducational training. This study is in progress and we presented preliminary results that can change with larger samples.

### TERAPIA OCUPACIONAL

**DEPRESSÃO E CAPACIDADE FUNCIONAL: ESTUDO EM IDOSOS RESIDENTES EM UMA COMUNIDADE** *Ana Elizabeth dos Santos Lins; Saulo Emmanuel da Silva Toledo; Gracinda Maria Gomes Alves; Amanda Kelly Loureiro de Lima; Stéphany Conceição Correia Alves Guedes*

**RESUMO. Introdução:** O envelhecimento da população é um fenômeno mundial, sendo este um processo dinâmico e progressivo, caracterizado por mudanças morfológicas, bioquímicas, psicológicas e funcionais. Durante esse processo as pessoas idosas podem estar sujeitas ao aparecimento de doenças crônicas, entre elas a depressão. A depressão é problema de saúde pública, no qual o indivíduo acometido, principalmente o idoso tem a tendência de se privar do convívio da família e dos amigos, podendo ter comprometimento funcional, limitando suas atividades cotidianas, contribuindo com a perda de papéis sociais, levando ao isolamento social, que no decorrer do tempo provoca perda prejudica sua qualidade de vida. **Objetivo:** Identificar a ocorrência de depressão entre os idosos residentes em uma comunidade do município de Maceió-AL, que já apresentavam dificuldades em realizar três ou mais Atividades de Vida Diária. **Métodos:** Estudo transversal descritivo e analítico, aprovado pelo CEP/UNCISAL no 1921/2012. Foram avaliados 50 idosos residentes na comunidade Riachuelo, no município de Maceió- Alagoas, cadastrados na Unidade Básica de Saúde Dr. Hélvio Auto. Os sujeitos foram entrevistados nos domicílios. Utilizou-se um questionário sóciodemográfico, Escala de Depressão Geriátrica (GDS-15) e o Mini Exame do Estado Mental (MEEM). **Resultados:** A prevalência de sintomatologia depressiva foi de 44%, sendo 30% de depressão leve e 14% de depressão moderada a grave, encontrada em 68,2% no sexo feminino, tendo escolaridade entre 1 a 8 anos de estudo (63,7%) e 63,6% eram viúvas. **Conclusão:** Os idosos dessa comunidade, além de já apresentarem comprometimento funcional ou seja dificuldade em realizar três ou mais atividades cotidianas, apresentaram presença de sintomas depressivos, podendo se tornarem cada vez mais dependentes de outrem, diminuindo sua autonomia. Esse estudo mostra a necessidade de acompanhamento desses idosos por uma equipe interdisciplinar, incluindo o terapeuta ocupacional com ações de reabilitação funcional (treinamento das atividades de vida diária -AVDs), orientação as famílias e avaliação e adaptação ambiental para que esses idosos possam ser ajudados a melhorar a capacidade funcional e a qualidade de vida. Descritores: Idoso; Atividade cotidiana; Depressão; Terapia Ocupacional.

**DIFFICULTIES IN DAILY ACTIVITIES REFERRED BY PEOPLE GOING THROUGH THE AGING PROCESS WITH OR WITHOUT SUSPICION OF DEPRESSION AND MILD COGNITIVE IMPAIRMENT** *Marina Picazzio Perez Batista; Rosé Colon Toldrá; Ana Cristina Fagundes Souto; Renata Guimarães Cordone; Andrea Toshye Sato; Maria Helena Morgani de Almeida*

**ABSTRACT.** Guided by the primary healthcare policies for elderly, occupational therapy contributes to maintain and improve the independence of this population in daily activities. **Objective:** To compare data collected by Occupation Therapy among people with or without suspicion of depression and mild cognitive impairment (MCI), that participated in the research “Envelhecer Mantendo Funções”. **Methods:** Descriptive and analytical study, with application of the CICAc Instrument: classification of the aged concerning self-care ability, through interviews with individuals aged fifty years old or older, between 2010 and 2012, at the Universidade de São Paulo University Hospital. It was decided to exclude from the data collection questions related to the activities of daily living, except communication ones, once functional changes for advanced daily activities allied to low performance in tests are precursor of dementia, even before those changes could be reflected in activities of daily living. The Pearson‘s chi-squared or Fischer‘s exact tests were used to comparative analysis at a 5% level of significance. **Results:** It was compared data of 200 participants: 177 subjects without suspicion of depression and MCI (G1) and 23 with suspicion of depression and MCI (G2). The groups did not differ in age (p=0,447), filiation (p=0,744), cohabitation (p=0,342), having caregiver (p=0,389) and owned housing (p=0,179). However, the G2 presented a higher proportion of women (p=0,008), subjects that do not rely on spouse (p=0,002), with four year or less of education (p=0,032), with economic resource limitation (p=0,007). None of the groups differ in difficulties related to instrumental activities (p=0,344) and paid working (p=0,690). Nevertheless, the G2 presented a greater occurrence of difficulties in communication and leisure (p=0,006 and p=0,041) and made higher reference to cognitive complaint to these activities (p=0,032). **Discussion:** These results correspond to data obtained in studies that associate depressive symptoms with difficulties in social activities (Klumb, 2001), cognitive decline with difficulties to leisure (Nilsson et al, 2006). **Conclusion:** The current study points to associations concerning suspicion of emotional and cognitive disorders, social and economic vulnerability and functional difficulties. Such associations could found interprofessional interventions designed for health promotion and quality of life of people going through the aging process.

**GROUP (RE) DISCOVERY** *Fabiana Maria Rampazo Mancin; Gisele Patricia Duarte*

**ABSTRACT.** Through clinical interventions conducted by occupational therapists was observed demand for male patients who had social isolation, impoverishment of interpersonal relationships, and difficulties due to a stroke and the decline of the aging process causing depressive symptoms and decreased daily activities. In clinical discussions was assessed that such characteristics of these patients interfered with the course of treatment, therefore, was conceived as a strategy, the intervention group format The purpose of this paper is to describe the actions proposed in the group of elderly men titled Group Re (Discovery) coordinated by the Occupational Therapy of a public secondary health. The group shut with six participants, weekly meetings (once a week) 1 hour and 30 minutes for six months. Participants are evaluated by the following protocols: - Mini Mental State Examination; - Geriatric Depression Scale 15; - MIF - Scale of Activities of Daily Living Lawton and Brody. In each of the meetings are used stretching techniques, relaxation, expressive activities, games, dynamic wheel, chat and lectures on disease prevention. Inclusion criteria: aged over 60 years, male and have chronic disease. Exclusion criteria: moderate to severe cognitive impairment. Through the reports of patients can be observed improvements related to emotional aspects, such as self-esteem and rediscovery of pleasurable activities. There were also improvements in the reduction of pain complaints and health problems, increase in adherence to treatment, and to adopt healthy behaviors. In some cases it was also possible to observe increased autonomy in the face of personal and family decisions. Thus, it is concluded that the interaction in the elderly group can share information and knowledge, as well as assess and devise strategies to overcome adversity daily. Furthermore, it becomes possible to approach the active aging at the expense of pathological.

**OFICINA DE PINTURA COMO ESTRATÉGIA DE HABILIDADES E COMPETÊNCIAS DO IDOSO EM INSTITUIÇÃO DE LONGA PERMANÊNCIA** *Gisele Monaco Dias; Ana Lucia Alves; Mercia Gomes Rodrigues; Renata Firpo R. Medeiros; Iracelia Munhoz; Audrey Andrade Bertolini; Roberto Dischinger Miranda*

**RESUMO. Introdução:** Através das atividades ocupacionais em uma Instituição de Longa Permanência (ILPI) é possível demonstrar a expressão de valores, a redescoberta de competências e habilidades, o compromisso, podendo envolver ainda convívio social. **Objetivo:** Avaliar a atividade de pintura como um meio de intervenção terapêutica em uma ILPI. **Métodos:** Estudo descritivo com análise qualitativa dos dados. A Oficina de Pintura é realizada uma vez por semana com duração de noventa minutos, sob a supervisão da Terapeuta Ocupacional. A Terapeuta Ocupacional auxilia os idosos que necessitam de ajuda individualmente, por isso limitamos para cinco o número de participantes por oficina. Os idosos são orientados em relação ao que irão pintar no dia e materiais que serão utilizados tais como: pincéis, tintas, telas, tecido e etc. São observados, o manejo do pincel, destreza, coordenação motora, bem como a autonomia em escolher as cores. A maioria dos idosos foi espontaneamente para a oficina, alguns precisavam ser convidados e estimulados pela terapeuta. **Resultados:** Participaram da Oficina um total de 10 idosos em uma ILPI, com dependência moderada à severa e idade média de 88,2 (±6,07) anos, sendo na sua maioria do sexo feminino. Na atividade de pintura pode-se observar um bom envolvimento entre os idosos, sendo que a pintura foi capaz de motivar discussões das mais variadas, relembrar vivências, além do compartilhar, ouvir, aprender, bem como um estímulo na interação social dos participantes em outros momentos do dia. **Conclusão:** A Oficina de pintura como instrumento terapêutico para os idosos se mostrou um recurso para as trocas de experiências e socialização. Serviu como um estímulo ao convívio social e iniciativa durante e após a execução das atividades.

**TRADUÇÃO E VALIDAÇÃO DO “SELF TEST II” - TESTE AUTOAPLICATIVO PARA RASTREAMENTO DE DEMÊNCIA** *Karol Casagrande Crepaldi; Francine Nunes Ferreira; Mateja de Leonni Stanonik; André Luiz Crepaldi; Tânia Corrêa de Toledo Ferraz Alves*

**RESUMO. Introdução:** O número de idosos que sofrem de doenças crônicas, incluindo déficits cognitivos, deverá aumentar nos próximos anos. Embora não exista cura definitiva para a demência, a melhor resposta ao tratamento é observada quando as intervenções são iniciadas nas primeiras fases da doença. No entanto, a demência geralmente não é detectada nos serviços de saúde, mesmo em estágios avançados. Neste contexto, os instrumentos de rastreamento têm um papel fundamental na identificação de alterações cognitivas e assim estabelecer, o mais rapidamente possível, o tratamento. Contudo, os testes disponíveis são de demorada aplicação e exigem supervisão especializada, o que limita sua utilização em ambientes clínicos com alta demanda. O Self Test II (STII) é um teste de rastreio para a demência que é auto -administrado e que, portanto, pode ser aplicado com supervisão mínima. **Objetivo:** Adaptar o ST II para o português brasileiro (ST II-Br) e compará-lo com a versão original em inglês; e avaliar a validade de construção da adaptação brasileira do STII, utilizando-a para avaliar indivíduos com diagnóstico de déficit cognitivo e, em seguida, comparar os resultados com os voluntários saudáveis. **Métodos:** Foram realizados quatro procedimentos (tradução, retrotradução, adaptação e pré-teste), a fim de realizar uma versão traduzida e adaptada para o português brasileiro do Self Test II. Nós também selecionamos 157 idosos com idade de 60 anos ou mais e com quatro ou mais anos de educação para participar do estudo de validação. O declínio cognitivo foi avaliado por meio do MEEM. Curva ROC e análises de correlação foram realizadas para o MEEM e o STII-Br. **Resultados:** Cento e cinco (66,9%) participantes eram do sexo feminino. A idade média dos entrevistados foi de 73 anos (60-98), e a escolaridade média foi de 7 anos (421). Cinquenta sujeitos (31,8%) apresentavam prejuízo cognitivo e 107 (68,2%) não apresentavam declínio cognitivo. A área sob a curva ROC foi de 0,84 (IC 95% 0,77-0,91). O ponto de corte do STII-Br que apresentou o melhor balanço entre sensibilidade e especificidade foi de 24/25 pontos (sensibilidade=82%, especificidade=76% e correta classificação dos casos=78%). **Conclusão:** O STII-Br mostrou-se um instrumento culturalmente apropriado e válido no rastreamento de declínio cognitivo em idosos com pelo menos quatro anos de escolaridade. O STII-Br pode ser incorporado como uma ferramenta útil nos serviços de saúde com alta demanda de atendimento.

